# PDK1-SGK1 Signaling Sustains AKT-Independent mTORC1 Activation and Confers Resistance to PI3Kα Inhibition

**DOI:** 10.1016/j.ccell.2016.06.004

**Published:** 2016-08-08

**Authors:** Pau Castel, Haley Ellis, Ruzica Bago, Eneda Toska, Pedram Razavi, F. Javier Carmona, Srinivasaraghavan Kannan, Chandra S. Verma, Maura Dickler, Sarat Chandarlapaty, Edi Brogi, Dario R. Alessi, José Baselga, Maurizio Scaltriti

**Affiliations:** 1 Human Oncology & Pathogenesis Program (HOPP), Memorial Sloan Kettering Cancer Center, 1275 York Avenue, Box 20, New York, NY 10065, USA; 2 MRC Protein Phosphorylation and Ubiquitylation Unit, College of Life Sciences, University of Dundee, Dow Street, Dundee DD1 5EH, Scotland; 3 Department of Medicine, Memorial Sloan Kettering Cancer Center, 1275 York Avenue, Box 20, Suite M2015, New York, NY 10065, USA; 4 Bioinformatics Institute (A^∗^STAR), 30 Biopolis Street, #07-01 Matrix, Singapore 138671, Singapore; 5 School of Biological Sciences, Nanyang Technological University, 60 Nanyang Drive, Singapore 637551, Singapore; 6 Department of Biological Sciences, National University of Singapore, 14 Science Drive 4, Singapore 117543, Singapore; 7 Department of Pathology, Memorial Sloan Kettering Cancer Center, 1275 York Avenue, Box 20, New York, NY 10065, USA

## Abstract

*PIK3CA*, which encodes the p110α subunit of PI3K, is frequently mutated and oncogenic in breast cancer. PI3Kα inhibitors are in clinical development and despite promising early clinical activity, intrinsic resistance is frequent among patients. We have previously reported that residual downstream mTORC1 activity upon treatment with PI3Kα inhibitors drives resistance to these agents. However, the mechanism underlying this phenotype is not fully understood. Here we show that in cancer cells resistant to PI3Kα inhibition, PDK1 blockade restores sensitivity to these therapies. SGK1, which is activated by PDK1, contributes to the maintenance of residual mTORC1 activity through direct phosphorylation and inhibition of TSC2. Targeting either PDK1 or SGK1 prevents mTORC1 activation, restoring the antitumoral effects of PI3Kα inhibition in resistant cells.

## Highlights

• The PDK1-SGK1 axis can sustain mTORC1 activity upon PI3K/AKT suppression
• SGK1 directly phosphorylates TSC2, resulting in mTORC1 activation
• Targeting PDK1 sensitizes *PIK3CA*-mutant cells to PI3Kα inhibition

## Graphical Abstract

**Figure:**
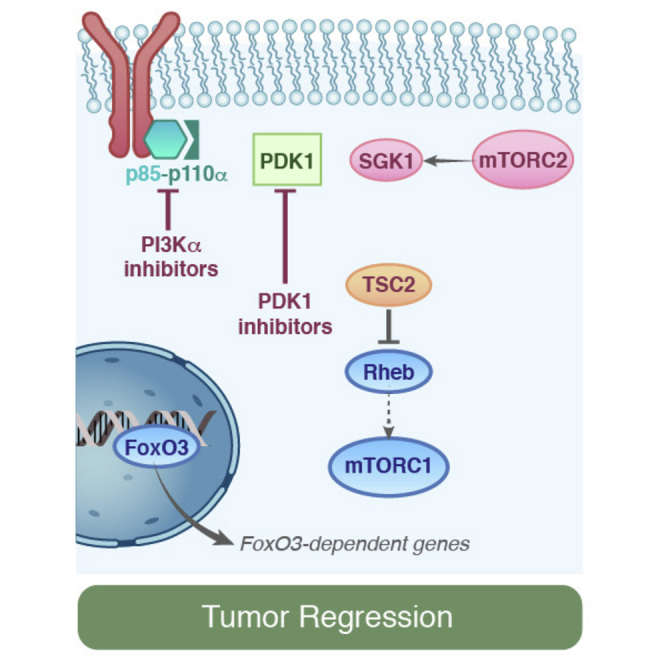

## Teaser

Castel et al. show that PDK1 activates SGK1 to phosphorylate and inhibit TSC2, contributing to maintenance of mTORC1 activity and resistance of PIK3CA-mutant cancer cells to PI3Kα inhibition. Targeting either PDK1 or SGK1 restores the sensitivity of these resistant cancer cells to PI3Kα inhibition.

## Significance

**Promising clinical activity has been reported in breast tumors bearing activating mutations in *PIK3CA*, the gene that encodes for the α isoform of the p110 catalytic subunit of PI3K. However, despite these encouraging results some tumors are resistant to these agents. In this work we show that, upon PI3Kα inhibition, the PDK1-SGK1 axis can overcome AKT inhibition by activating mTORC1 via direct phosphorylation of TSC2. Therefore, both AKT and PDK1 must be suppressed to achieve full mTORC1 inhibition and antitumor activity in these tumors resistant to PI3Kα inhibition. This study uncovers a mechanism of cell survival under pharmacological pressure that can be exploited to improve the therapeutic options of patients with breast cancer.**

## Introduction

The phosphoinositide 3-kinase (PI3K) pathway integrates many extracellular stimuli and triggers the phosphorylation of key downstream effectors such as AKT and the mammalian Target of Rapamycin Complex 1 and 2 (mTORC1 and 2). This signaling cascade is essential for regulating cell size, proliferation, survival, and metabolism (Engelman, 2009, Thorpe et al., 2015). Activation of PI3K results in increased phosphatidylinositol-(3,4,5)-triphosphate (PIP_3_) at the plasma membrane, promoting the recruitment of the pleckstrin homology (PH) domain-containing proteins PDK1 and AKT (Currie et al., 1999, Pearce et al., 2010), where the constitutively active kinase PDK1 phosphorylates AKT at the activation loop (T308) (Alessi et al., 1997) and mTORC2 in the hydrophobic motif (S473) (Sarbassov et al., 2005). Once active, AKT phosphorylates a variety of antiapoptotic and cell-cycle-related proteins as well as transcription factors (Manning and Cantley, 2007). Moreover, AKT activates downstream mTORC1 through the phosphorylation of the negative regulators TSC2 and PRAS40 (Inoki et al., 2002, Manning et al., 2002, Potter et al., 2002, Sancak et al., 2007).

Activating mutations in *PIK3CA*, which encodes the α isoform of the p110 catalytic subunit of PI3K, results in hyperactivation of the PI3K/AKT/mTOR pathway (Engelman, 2009). These mutations are common in breast cancer and provide the rationale for the development of inhibitors targeting the different nodes of the PI3K pathway (Fruman and Rommel, 2014).

PI3Kα-specific inhibitors are showing promising results in patients with tumors bearing activating *PIK3CA* mutations, but not all such tumors are sensitive (Juric et al., 2012, Juric et al., 2013). Understanding the molecular mechanisms by which tumors bypass the pharmacological inactivation of PI3Kα is crucial for the identification of patients more likely to respond to these inhibitors and for devising therapeutic strategies to improve the clinical benefit.

We previously reported that the activation status of mTORC1 upon PI3Kα blockade is a determinant of drug sensitivity in *PIK3CA*-mutant tumors. Despite full inhibition of PI3K/AKT, the presence of residual mTORC1 activity is sufficient to weaken the antitumor activity of PI3Kα inhibition. Resistant tumors are sensitized by co-treatment with the mTORC1 allosteric inhibitor everolimus, underscoring the causative role of mTORC1 in limiting the effects of PI3Kα blockade (Elkabets et al., 2013). In this work, we elucidate the molecular pathway that allows mTORC1 to retain activity in the presence of PI3K and AKT inactivation.

## Results

### PDK1 Inhibition Sensitizes Resistant Cells to BYL719

Aiming to identify possible kinases or phosphatases responsible for the AKT-independent sustained mTORC1 activity in cells resistant to PI3Kα inhibition, we performed a small interfering RNA (siRNA) screen using a library targeting 710 kinases and 298 phosphatases encoded in the human genome. We measured cell viability and S6 ribosomal protein (S6) phosphorylation, a bona fide readout of mTORC1 activity, in the presence of BY719, a PI3Kα-specific inhibitor.

The screen design is shown in Figure S1A. Three different siRNAs targeting each member of the kinome/phosphatome were transfected in JIMT1 and HCC1954 cell lines, both of which are *PIK3CA* mutant and insensitive to BYL719. After treatment over 6 days, we found that knockdown of 37 enzymes in HCC1954 and 35 enzymes in JIMT1 sensitized cells to PI3Kα inhibition (Figure S1B and Table S1). Among these, five were found to be shared in both cell lines: *MTOR*, *PDPK1*, *PIK3CA*, *PPP1R12A*, and *PAPL* (Figure S1B). These findings were validated with a second targeted screening using the two most active siRNAs against each of these five targets, interrogating for both cell viability and phosphorylation of S6. With this more stringent approach, we found that only knockdowns of mTOR and PDK1 were capable of reducing S6 phosphorylation (S240/4) in the presence of PI3Kα inhibition (Figure S1C). While the finding of mTOR confirmed our previous data (Elkabets et al., 2013), the contribution of PDK1 in maintaining the resistant phenotype was an original finding.

**Figure 1:**
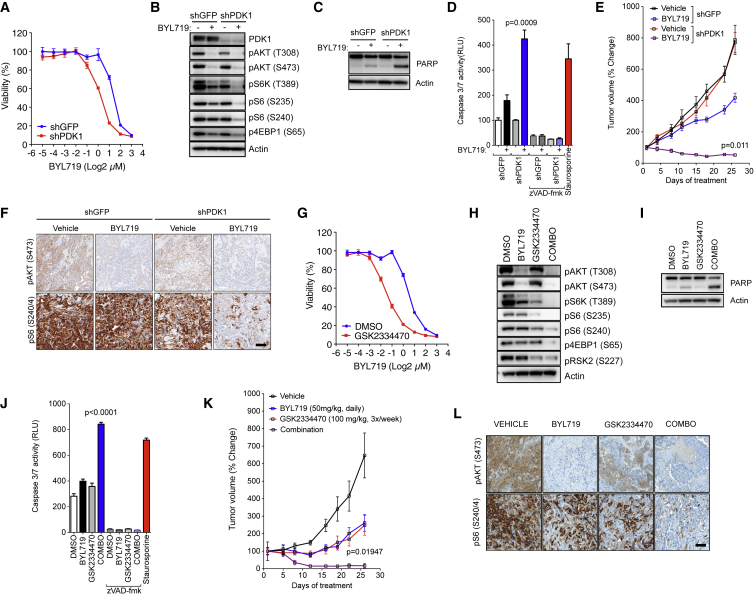
PDK1 Inhibition Sensitizes Resistant Cells to BYL719 (A) Dose-response curves from HCC1954 cells transduced with shGFP and shPDK1 and treated with BYL719 for 6 days. (B) Western blot comparing cells from (A) treated with BYL719 (1 μM) for 4 hr. (C) PARP western blot in cells transduced with shGFP and shPDK1 and treated with BYL719 (1 μM) for 24 hr. (D) Caspase 3/7 DEVDase activity of HCC1954 shGFP and shPDK1 cells treated with BYL719 (1 μM) for 12 hr in the presence or absence of caspase inhibitor zVAD-fmk (20 μM). Staurosporine was used as a positive control (1 μM; 4 hr). (E) HCC1954 shGFP and shPDK1 xenografts treated with vehicle or BYL719 (n = 10/arm). (F) IHC analysis of tumors from (E) collected at the end of the experiment after 4 hr of the last treatment. Scale bar, 100 μm. (G) Dose-response curves from HCC1954 cells treated with BYL719 in the presence or absence of GSK2334470 (1 μM) over 6 days. (H) Western blot comparing HCC1954 cells treated with BYL719 (1 μM), GSK2334470 (1 μM), or the combination of both agents for 4 hr. (I) Western blot of PARP in cells treated for 24 hr. (J) Caspase 3/7 DEVDase activity of lysates from HCC1954 cells treated with BYL719 (1 μM), GSK2334470 (1 μM), or the combination of both agents for 12 hr in the presence or absence of caspase inhibitor zVAD-fmk (20 μM). Staurosporine was used as a positive control (1 μM, 4 hr). (K) HCC1954 xenografts treated with vehicle, BYL719 (25 mg kg^−1^), GSK2334470 (100 mg kg^−1^), or the combination of both agents (n = 10/arm). (L) IHC analysis of tumors from (K) collected at the end of the experiment after 4 hr of the last treatment. Scale bar, 100 μm. p Values were calculated using Student's t test. Error bars denote ±SEM. See also Figures S1 and S2.

PDK1 is a kinase that belongs to the Containing PKA, PKG, and PKC (AGC) kinase family that includes AKT, PKC, RSK, SGK, and S6K (Pearce et al., 2010). To confirm that PDK1 limits the sensitivity to PI3Kα inhibition by maintaining mTORC1 activity upon PI3Kα inhibition, we generated HCC1954 and JIMT1 cell lines stably expressing a PDK1 short hairpin RNA (shRNA). We observed that PDK1 knockdown is sufficient to decrease cell viability upon BYL719 treatment (Figures 1A and S2A). As previously described, treatment with BYL719 alone reduced AKT phosphorylation (S473 and T308) but not downstream mTORC1 targets (Elkabets et al., 2013). In contrast, the combination of PDK1 knockdown with BYL719 decreased the phosphorylation of the mTORC1 downstream targets p70 S6 kinase (S6K) and translation initiation factor 4E-binding protein (4EBP1), as well as phosphorylated S6 at both S240/4 and S235/6 sites (Figures 1B and S2B). As a result, the combination of BYL719 and PDK1 knockdown decreased cap-dependent translation (Figure S2C), a cellular process directly regulated by mTORC1 (Silvera et al., 2010). In PDK1 knockdown cells, inhibition of PI3Kα induced an increased binding of 4EBP1 to the cap m^7^GpppN mRNA analog m^7^GTP, to a similar extent as the mTOR kinase inhibitor AZD8055. On the contrary, we observed a reduction of the eukaryotic initiation factors (eIF) eIF4G and eIF4A, components of the eIF4F cap-initiation complex. As expected, eIF4E remained unchanged. In long-term treatments, the combination of BYL719 and PDK1 knockdown induced poly(ADP-ribose)polymerase (PARP) cleavage (Figure 1C) and increased caspase 3/7 activity (Figure 1D), surrogate markers of apoptotic activity.

Pharmacological inhibition of PI3Kα resulted in a modest delay in tumor growth in shGFP control xenografts but was sufficient to induce durable tumor shrinkage in tumors with ablated PDK1 (Figure 1E). Analysis of the tumors showed that BYL719 treatment effectively suppressed AKT phosphorylation (S473) in both shGFP and shPDK1 tumors, whereas S6 and 4EBP1 phosphorylation was inhibited only in shPDK1 xenografts (Figures 1F and S2D).

Next, we tested the activity of BYL719 in combination with GSK2334470, a highly selective PDK1 inhibitor (Najafov et al., 2011). We determined the appropriate dose of GSK2334470 to be used in combination with PI3Kα inhibition by analyzing both phosphorylation of the PDK1 target RSK2 (S227) and cell viability upon incubation with increasing concentrations of the PDK1 inhibitor. At 1 μM, pRSK2 (S227) was appreciably reduced (Figure S2E) with no significant changes in cell viability (Figure S2F). Despite the minimal effect on cell viability when used as a single agent, treatment with GSK2334470 was sufficient to sensitize both HCC1954 and JIMT1 cells and the triple-negative BYL719-resistant breast cancer cell line BT20 to PI3Kα inhibition (Figures 1G and S2G–S2H). Again, we observed that only the combination of BYL719 and GSK2334470 resulted in the inhibition of AKT and mTORC1 (Figures 1H, S2I, and S2J). Some residual pS6 was observed in BT20 cells, which might be attributed to the heterogeneity of the cell line or additional mechanisms that regulate S6 phosphorylation.

PDK1 inhibition did not decrease the phosphorylation of AKT at the activation loop (T308) as a result of a compensatory mechanism involving PIP3 and mTORC2, an observation in line with previous reports (Najafov et al., 2012). Analysis of cap-dependent translation complex formation revealed an increase in 4EBP1 and a decrease in eIF4G and eIF4A in m^7^GTP pulldowns when both drugs were combined, consistent with mTORC1 inhibition (Figure S2K). Consistent with the knockdown experiments, the combination of BYL719 and GSK2334470 induced apoptosis in HCC1954 cells when measured by PARP cleavage (Figure 1I) and caspase 3/7 activity (Figure 1J).

We then expanded our results in vivo. Although some antitumor activity was observed with treatment with BYL719 or GSK2334470, only the combination of both compounds induced durable tumor shrinkage (Figure 1K) and inhibition of pAKT (S473), pS6 (S240/4), and p4EBP1 (T37/46) (Figures 1L and S2L–S2O).

Taken together, these results indicate that PDK1 inhibition sensitizes cells to PI3Kα blockade via suppression of mTORC1.

### The PIF-Binding Pocket of PDK1 Is Required for Sustained mTORC1 Activation upon PI3Kα Inhibition

The activation of AGC kinases requires phosphorylation at two highly conserved regulatory motifs termed the hydrophobic motif (HM) and the activation loop. Several kinases prime AGC kinases for activation through phosphorylation at the HM. PDK1, which acts as a master regulator of this family of kinases, scaffolds at the phosphorylated HM using the PIF (PDK1-interacting fragment) binding pocket. This interaction enables phosphorylation of the activation loop, thereby fully activating their activity. However, AKT does not require the PIF-binding pocket of PDK1 but instead needs its PH domain to interact with PDK1 at the plasma membrane in a PIP_3_-dependent manner (Alessi et al., 1997, Arencibia et al., 2013, Biondi et al., 2001, Collins et al., 2003, McManus et al., 2004). To explore the PDK1 regulatory mechanism required to sustain mTORC1 activity upon PI3Kα inhibition, we used the HCT116 parental and *PDPK1*-null (*PDPK1*^−/−^) isogenic model (Ericson et al., 2010). HCT116 cells harbor the H1047R *PIK3CA*-activating mutation, and the addition of BYL719 decreased AKT phosphorylation but did not decrease mTORC1 signaling, mimicking the phenotype observed in BYL719-resistant breast cancer cell lines. However, in *PDPK1*^−/−^ cells, the addition of BYL719 inhibited mTORC1 (Figure S2P).

We reconstituted HCT116 *PDPK1*^−/−^ cells with the wild-type (WT), kinase-inactive K111N (KD), PIP_3_-binding deficient K546E (KE), and PIF-pocket-deficient L155E (LE) mutants (Figure S2Q) to test the contribution of each regulatory mechanism of PDK1 to mTORC1 activation. Reconstitution of PDK1 WT, but not the mutant KD, restored mTORC1 activation in the presence of BYL719. The mutant KE was also able to restore the phenotype. On the other hand, the mutant LE was unable to rescue mTORC1 signaling (Figure S2R). This set of experiments suggests that the maintenance of mTORC1 activity requires both kinase activity and the PIF-binding pocket of PDK1 but is PIP_3_- and, consequently, AKT-independent.

### Combined Suppression of PI3Kα and PDK1 Activates FOXO-Dependent Transcription

**Figure 2:**
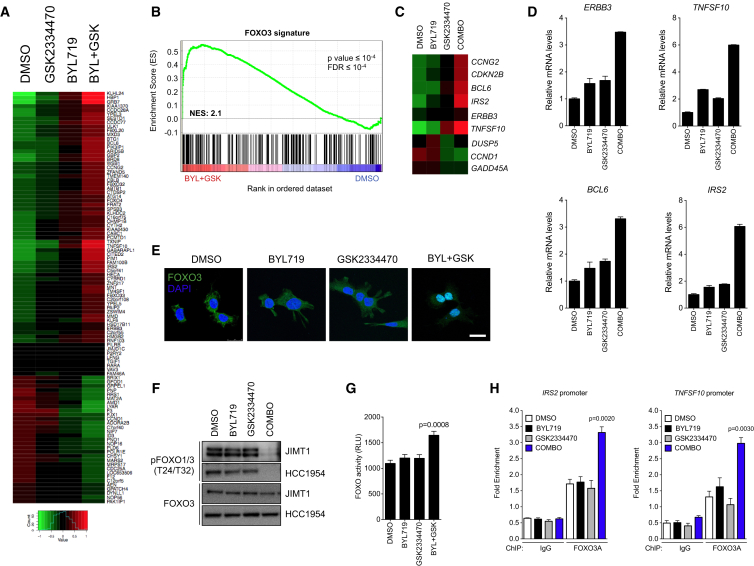
FOXO Activation upon PDK1 and PI3Kα Inhibition (A) Changes in the top 200 differentially expressed genes in HCC1954 and JIMT1 cells treated with DMSO, BYL719 (1 μM), GSK2334470 (1 μM), or the combination of both agents for 4 hr. Gene upregulation is in red and gene downregulation is in green. (B) Enrichment plot for the FOXO3 signature in HCC1954 cells. NES, normalized enrichment score. (C) Heatmap showing changes in expression of FOXO3 targets in HCC1954 and JIMT1 cells. (D) mRNA expression in HCC1954 cells treated with DMSO, BYL719 (1 μM), GSK2334470 (1 μM), or the combination of both agents for 4 hr. (E) Representative images of FOXO3 immunofluorescence (green) in HCC1954 cells treated with DMSO, BYL719 (1 μM), GSK2334470 (1 μM), or the combination of both agents for 4 hr. Nuclei are shown in blue (DAPI). Scale bar, 25 μm. (F) Western blot analysis of FOXO1/3 phosphorylation (T24/T32) in HCC1954 and JIMT1 cells treated with DMSO, BYL719 (1 μM), GSK2334470 (1 μM), or the combination of both agents for 4 hr. (G) Luciferase reporter assay in HCC1954 cells stably transduced with the FOXO consensus motif reporter construct treated as indicated for 12 hr. RLU, relative light units. (H) ChIP-qPCR assay of FOXO3A binding at *TNFSF10A* and *IRS2* promoters in HCC1954 cells treated as indicated in (F). p Values were calculated using Student's t test. Error bars denote ±SEM. See also Figure S3.

We next performed gene-expression analysis to investigate whether mTORC1 suppression is accompanied by specific transcriptional changes. While the differences in gene expression upon BYL719 or GSK2334470 treatment were modest, the combination of both induced marked changes when compared with the DMSO-treated control cells (Figures 2A and S3A). Gene set enrichment analysis of these data showed enrichment of FOXO3 transcription factor targets in both cell lines (Figures 2B and S3B). Individual genes described to be positively (*CCNG2*, *BCL6*, *IRS2*) or negatively (*CCND1*) regulated by FOXO3 (Webb and Brunet, 2014) were confirmed to be induced or repressed, respectively, upon dual PI3Kα and PDK1 blockade (Figure 2C). These results were further validated by performing qRT-PCR of four well-described FOXO3 targets, *ERBB3*, *TNFSF10*, *BCL6*, and *IRS2* (Webb and Brunet, 2014), following different treatments (Figures 2D and S3C).

Upon growth factor stimulation, FOXO transcription factors are phosphorylated at several residues and retained in the cytosol by the 14-3-3 proteins (Webb and Brunet, 2014). Inhibition of these mitogenic signals induces a rapid dephosphorylation and nuclear translocation of FOXOs that allows expression of downstream target genes involved in apoptosis and/or cell-cycle arrest (Webb and Brunet, 2014). In our cells, we found that treatment with both BYL719 and GSK2334470, but not either single agent, resulted in strong nuclear localization of FOXO3 (Figures 2E and S3D). This was consistent with a decreased phosphorylation of this transcription factor at residue T32 (Figure 2F). Moreover, we observed that only the combination stimulated endogenous FOXO transcriptional activity (Figure 2G) and increased occupancy of FOXO3A at the promoters of two well-known FOXO targets, *IRS2* and *TNFSF10* (Figures 2H and S3E). These results suggest that dual PI3Kα and PDK1 inhibition induces a FOXO-dependent transcriptional activity in BYL719-resistant cells.

### SGK1 Is Upregulated in BYL719-Resistant Cell Lines and in Tumors from Patients Refractory to PI3Kα Inhibition

AKT has been shown to phosphorylate FOXO1 and FOXO3 at T24 and T32 residues, respectively (Brunet et al., 1999). However, we observed that despite full inhibition of AKT by PI3Kα inhibition, FOXO3 was not efficiently primed to migrate to the nucleus and exert its transcriptional activity in cells resistant to BYL719 (Figures 2 and S3). Since PDK1 requires downstream AGC kinases as molecular effectors (Pearce et al., 2010), we reasoned that in BYL719-resistant cells a downstream AGC kinase dependent on the PDK1 catalytic activity and docking with the PIF-binding pocket (Figure S2R) regulates both FOXO1/3 phosphorylation and mTORC1 activity, independently of AKT.

Serum and glucocorticoid-induced kinase (SGK) is a family of AGC serine/threonine kinases that comprises three members (SGK1–3) highly homologous to AKT (Kobayashi and Cohen, 1999). SGK1 activation is mediated by mTORC2-dependent phosphorylation at the HM (S422) and subsequent PDK1 phosphorylation at the activation loop (T256) in a PIF-binding pocket-dependent manner (Garcia-Martinez and Alessi, 2008, Pearce et al., 2010). Earlier reports have demonstrated that SGK1 is able to directly phosphorylate FOXO1 at residues T32 and S315 (Brunet et al., 2001) and has been correlated with resistance to AKT inhibition (Sommer et al., 2013). Therefore, we hypothesized that SGK1 plays a critical role downstream of PDK1 in sustaining mTORC1 activity and inducing resistance to PI3Kα inhibition.

**Figure 3:**
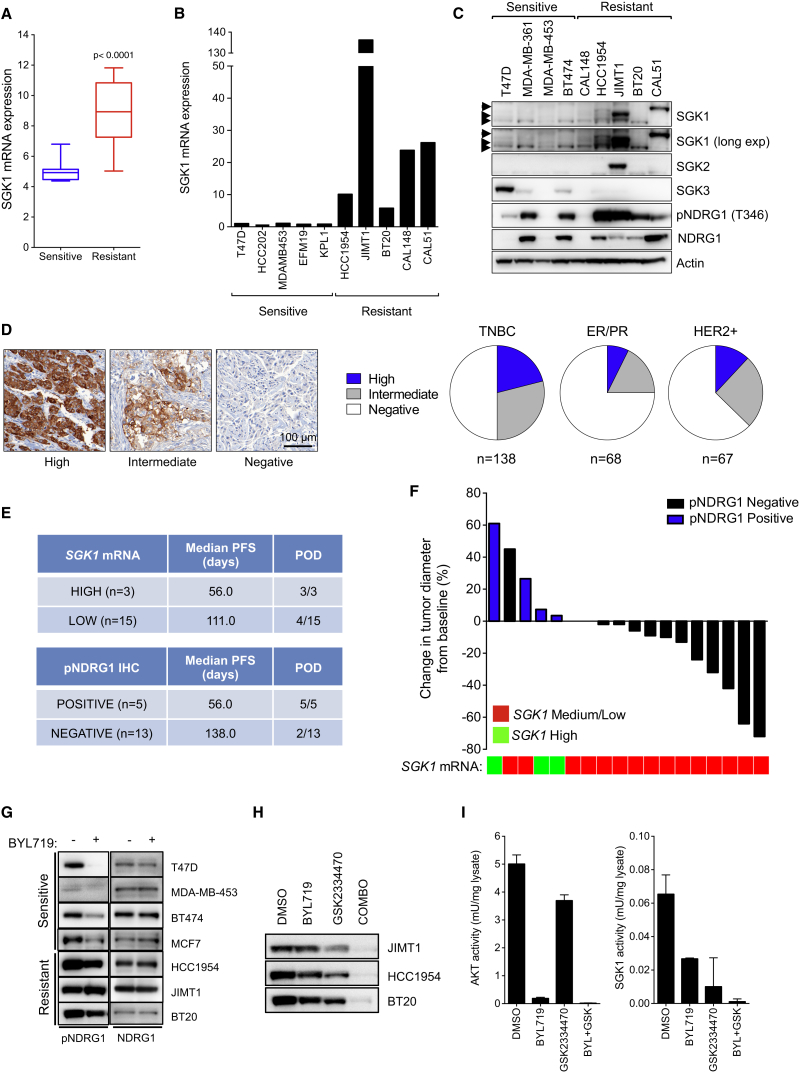
SGK1 Upregulation in BYL719-Resistant Cell Lines (A) *SGK1* mRNA levels in breast cancer cell lines sensitive or resistant to BYL719 (n = 27). Box indicates the median and the interquartile range, and whiskers represent minimum and maximum. (B) *SGK1* mRNA levels in a panel of *PIK3CA*-mutant breast cancer cell lines sensitive or resistant to BYL719. (C) Western blot analysis of SGK1, SGK2, SGK3, and phosphorylated NDRG1 in a panel of *PIK3CA*-mutant breast cancer cell lines. Arrowheads indicate the SGK1 isoforms. (D) Representative images of phosphorylated NDRG1 (T346) IHC in breast cancer tumors and quantification of the stainings observed in a cohort of 273 breast cancer cases. (E) Summary of the median number of days of progression-free survival (PFS) and the number of patients experiencing progression of disease (POD) as best response according to RECIST criteria in association with *SGK1* mRNA levels and positivity to pNDRG1 staining by IHC. (F) Waterfall plot showing changes in tumor size of the patients included in the study. Heatmap represents the *SGK1* mRNA levels for each tumor sample. (G) Western blot for NDRG1 and phosphorylated NDRG1 (T346) in BYL719-sensitive and -resistant breast cancer cell lines treated with BYL719 (1 μM) for 4 hr. (H) Western blot of phosphorylated NDRG1 (T346) in resistant cells treated with DMSO, BYL719 (1 μM), GSK2334470 (1 μM), or the combination of both agents for 4 hr. (I) Endogenous kinase assay for SGK1 and AKT in HCC1954 cells treated with DMSO, BYL719 (1 μM), GSK2334470 (1 μM), or the combination of both agents for 4 hr. p Values were calculated using Student's t test. Error bars denote ±SEM. See also Figure S4.

We analyzed the basal mRNA expression of 27 breast cancer cell lines, previously characterized as sensitive or resistant to BYL719 (Elkabets et al., 2013), and found that resistant cell lines had significantly higher levels of *SGK1* mRNA compared with sensitive cells (Figures 3A and S4A). This held true when only breast cancer cells harboring *PIK3CA*-activating mutations, which are known to be sensitive to PI3Kα inhibition (Elkabets et al., 2013), were considered in the analysis (Figures 3B and 3C). The mRNA levels of *SGK2* and *SGK3* were similar between sensitive and resistant cell lines (Figure S4B), although JIMT1 cells also express high levels of SGK2. The ratio of phosphorylated N-Myc Downstream Regulated 1 (NDRG1) (T346), a substrate of SGK1 (Murray et al., 2004), versus total NDRG1 was also higher in BYL719-resistant cells (Figures 3C and S4C). Both CAL-148 and CAL-51 cells carry mutations in *PTEN* (Cerami et al., 2012), and their resistance to BYL719 may be due to insufficient inhibition of the PI3K/AKT pathway as a consequence of PI3Kβ activity (Juric et al., 2015). However, BYL719, but not the PI3Kβ inhibitor AZD6482, fully decreases pAKT levels in both CAL-148 and CAL-51 cells (Figure S4D).

Given the lack of reliable results obtained with commercially available antibodies against SGK1, we analyzed the expression of pNDRG1 (T346) in 273 breast invasive carcinomas, comprising 138 triple-negative breast cancer (TNBC), 68 estrogen-/progesterone-receptor-positive, and 67 human epidermal growth factor 2 (HER2)-positive breast cancer patients. High pNDRG1 staining was found in TNBC (21%) and HER2-positive tumors (12%) (Figure 3D), a finding in line with the percentage of breast cancer samples expressing high levels of *SGK1* in the Cancer Genome Atlas cohort (Ciriello et al., 2015).

We then explored whether SGK1 and pNDRG1 expression correlate with clinical outcome to PI3Kα inhibition by analyzing *PIK3CA*-mutant breast cancer samples from 18 patients treated with BYL719 in combination with an aromatase inhibitor (NCT01870505). Three of these tumors expressed high levels of *SGK1* mRNA while the remaining 15 had medium to low levels of *SGK1* mRNA. The three patients with tumors exhibiting high SGK1 expression, which also stained positive for pNDRG1, did not respond to therapy (Figures 3E and 3F). Two patients with tumors expressing medium to low levels of SGK1 stained positive for pNDRG1 and rapidly progressed. On the contrary, in the group of patients with pNDRG1-negative tumors, three had partial responses and eight had stable disease by RECIST (response evaluation in solid tumors) criteria (Therasse et al., 2000). This was in agreement with the longer time to disease progression of this subset of patients when compared with the SGK1-high/pNDRG1-positive cohort (Figures 3E and 3F). Although suggestive of a role of SGK1 in mediating intrinsic resistance to PI3Kα inhibitors, these results should be validated in larger cohorts of patients.

We then sought to investigate the mechanism underlying this variability in SGK1 expression. We analyzed the promoter of *SGK1* and realized that in the region between −56 bp and +391 bp of the transcription start site there are 12 CpG sites that are susceptible for DNA methylation. Using bisulfite sequencing we found that three of these CpG sites were differentially methylated between sensitive and resistant cell lines (Figure S4E). We confirmed our results quantitatively using direct pyrosequencing in 11 cell lines (8 sensitive and 3 resistant to PI3Kα inhibition). Sensitive cell lines exhibited high levels of *SGK1* promoter methylation (mean CpG_1_ = 65%, CpG_2_ = 67%, and CpG_3_ = 40%), while resistant cell lines displayed low levels of *SGK1* promoter methylation (mean CpG_1_ = 11%, CpG_2_ = 13%, and CpG_3_ = 16%) (Figure S4F). The degree of promoter DNA methylation inversely correlated with *SGK1* mRNA levels in these cells (Figure S4G). By chromatin immunoprecipitation (ChIP)-qPCR assays, we found high occupancy of RNA polymerase II (Pol II), an enzyme essential for transcription, and phosphorylated (S5) Pol II in both HCC1954 and JIMT1 cells, indicating that *SGK1* transcription is active in these resistant cell lines (Figure S4H). On the contrary, in the sensitive cell lines MDA-MB-453 and T47D we found low occupancy of both Pol II and phosphorylated Pol II (S5) in the *SGK1* promoter (Figure S4H). Treatment with the DNA demethylating agent 5-Aza-2′-deoxycytidine and the histone deacetylase inhibitor panobinostat reduced *SGK1* promoter DNA methylation (data not shown) and increased mRNA levels of *SGK1* in the four sensitive cell lines tested (Figure S4I). Our results indicate that the differential expression of *SGK1* is mediated, at least in part, by epigenetic regulation.

Although pNDRG1 and SGK1 expression correlates in vivo (Murray et al., 2004), AKT can also phosphorylate NDRG1 in the absence of SGK1 in cultured cell lines (Sommer et al., 2013). In support of these observations, cancer cells sensitive to BYL719 displayed decreased NDRG1 phosphorylation at T346 when treated with BYL719 (Figure 3G). In contrast, resistant cell lines treated with BYL719 maintain NDRG1 phosphorylation, underscoring the role of SGK1 in this setting. Central to our work, only the combination of BYL719 and GSK2334470 was able to decrease the phosphorylation of NDRG1 in BYL719-resistant cell lines, confirming that the combination of both drugs is required to effectively inhibit both SGK1 and AKT activity (Figure 3H).

Next, we immunoprecipitated endogenous SGK1 and found that BYL719 treatment was not sufficient to completely abolish the kinase activity of the enzyme, in contrast to GSK2334470 (Figure 3I). On the other hand, immunoprecipitation of endogenous AKT revealed that while BYL719 treatment completely abrogated AKT kinase activity, this is not the case when cells are treated with GSK2334470, as previously observed (Najafov et al., 2012). This is indicative of a signaling compensation between AKT and SGK1 and that only the combination of PI3Kα and PDK1 inhibitors can simultaneously block the activity of the endogenous enzymes in resistant cells. While mTORC2-mediated phosphorylation at the HM is indispensable for SGK1 kinase activity (Kobayashi and Cohen, 1999), several reports indicate that AKT remains active in the absence of HM phosphorylation, as phosphorylation at the activation loop (T308) is sufficient to partially activate the kinase (Guertin et al., 2006, Jacinto et al., 2006, Rodrik-Outmezguine et al., 2011). Treatment of HCC1954 cells with the mTOR catalytic inhibitor AZD8055, which targets both mTORC1 and mTORC2 and completely inhibits SGK1 but not AKT activity, did not reduce the levels of the substrates pFOXO3 (T32) and pNDRG1 (T346), confirming that mTORC2 inhibition is not sufficient to abolish AKT activity in these cells (Figure S4J).

Consistent with previous results, GSK2334470 alone is not capable of inhibiting AKT activity (Figures S4J and 3I). Therefore, in the presence of mTOR inhibition the phosphorylation at the HM is abrogated and this mechanism of AKT activation is no longer supported. Addition of GSK2334470 to resistant cells treated with AZD8055 resulted in a marked decrease in the phosphorylation of both FOXO3 and NDRG1. This translated in decreased cell viability in both HCC1954 and JIMT1 cells, phenocopying the effects observed by dual PDK1 and PI3Kα inhibition (Figure S4K). Similar results were found when RICTOR, a key mTORC2 component, was knocked down in the presence of PDK1 inhibition (Figure S4L).

These results demonstrate that both PI3K and PDK1 activities have to be suppressed to inhibit downstream AKT and SGK1 phosphorylation and activity in our resistant models.

### SGK1 Mediates Resistance to BYL719

**Figure 4:**
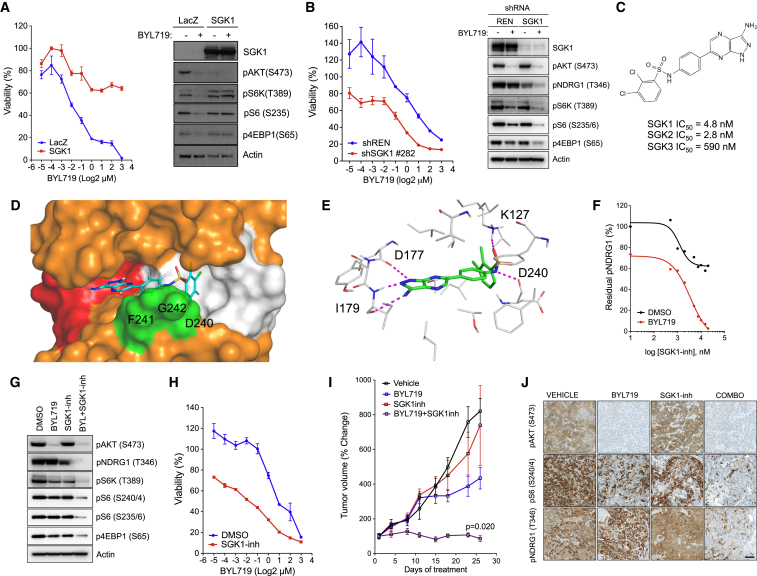
SGK1 Inhibitor Sensitizes Resistant Cells to BYL719 (A) (Left) Dose-response curves from MDA-MB-361 cells transduced with pLenti7.3-LacZ or pLenti7.3-SGK1 (Δ60, S422D) constructs and treated with increasing concentrations of BYL719 for 6 days. (Right) Western blot analysis of LacZ and SGK1 transduced MDA-MB-361 cells treated with BYL719 (1 μM) for 4 hr. (B) (Left) Growth curves of HCC1954 cells stably expressing doxycycline-inducible control (REN) or SGK1 knockdown treated with increasing concentrations of BYL719 for 6 days. (Right) Western blot analysis of GFP-sorted control (REN) and SGK1 shRNA cells treated with BYL719 (1 μM) for 4 hr. (C) Chemical structure of SGK1-inh and in vitro SGK1 kinase activity assay in the presence of increasing concentrations of SGK1-inh. IC_50_ for SGK1, SGK2, and SGK3 are indicated. (D) Docking overview of SGK1-inh in the DFG-out conformation of SGK1. The hinge region is colored in red, the DFG motif in green, the “allosteric” hydrophobic cavity that results from the DFG flip in grey, and the rest of the kinase in orange. The DFG motif amino acids are indicated (D240, F241, and G242). (E) Detailed residues that mediate the interaction between SGK1-inh and the inactive conformation of SGK1. Hydrogen bonds are shown as purple dotted lines. (F) Western blot quantification of NDRG1 phosphorylation (T346) in HCC1954 cells treated with increasing concentrations of SGK1-inh for 4 hr in the absence or presence of BYL719 (1 μM). (G) Western blot analysis of HCC1954 cells treated with BYL719 (1 μM), SGK1-inh (10 μM), or both agents for 4 hr. (H) Dose-response curves from HCC1954 cells treated with BYL719 for 6 days in the absence or presence of SGK1-inh. (I) HCC1954 xenograft treated with vehicle, BYL719 (25 mg kg^−1^), SGK1-inh (50 mg kg^−1^), or the combination of both agents (n = 10/arm). (J) IHC analysis of tumors from (K) collected at the end of the experiment 4 hr after the last dosage. Scale bar, 100 μm. p Values were calculated using Student's t test. Error bars denote ±SEM. See also Figure S5.

We next assessed the contribution of SGK1 in mediating resistance to PI3Kα inhibition. The overexpression of a constitutively active form of SGK1 in MDA-MB-361 cells, which are sensitive to PI3Kα inhibition, was sufficient to increase cell viability in the presence of BYL719 (Figure 4A). In parental cells, PI3Kα inhibition decreased both AKT phosphorylation and mTORC1 signaling, while cells overexpressing SGK1 maintained mTORC1 signaling in the presence of BYL719. Given that genetic inactivation of SGK1 is toxic (Sommer et al., 2013), we generated doxycycline-inducible shRNA targeting SGK1. Upon SGK1 knockdown we observed a decrease in cell viability that was enhanced in the presence of BYL719 (Figure 4B). Accordingly, SGK1 knockdown decreased pNDRG1 and mTORC1 target levels only when combined with PI3Kα inhibition.

Next, we studied the effects of pharmacological inhibition of SGK1 in our models. The few SGK inhibitors currently available have low activity in cellular models. To overcome this problem, we characterized a recently described SGK inhibitor (SGK1-inh) (Halland et al., 2015). SGK1-inh exhibited an IC_50_ of 4.8 nM at 10 μM ATP using a recombinant SGK1 kinase assay, with appreciable activity also toward SGK2 and SGK3 (IC_50_ of 2.8 nM and 590 nM, respectively) (Figure 4C). The specificity of this compound was tested in vitro at a concentration of 1 μM (200-fold higher than the SGK1 inhibitory dose) against a panel of 140 human kinases showing remarkable selectivity toward SGK1 (Figure S5A). Despite detecting no activity against AKT1, PDK1, PKC, or RSK, we found that at this high concentration S6K was also inhibited, probably due to the high similarity of their catalytic sites. Because S6K is a key downstream substrate of mTORC1, we aimed to further characterize the activity of SGK1-inh toward S6K. Recombinant in vitro kinase assay of S6K demonstrated an IC_50_ of 33 nM, seven times higher than the IC_50_ for SGK1 (Figure S5B). At the cellular level, we performed an S6K kinase assay in 293T cells overexpressing constitutively active S6K (ΔCT T389E) treated with increasing concentrations of SGK1-inh, and found an IC_50_ of ∼20 μM (Figure S5C). Next, using two fibroblast cell lines that lack TSC2 (derived from TSC2 knockout mice and a lymphangioleiomyomatosis patient, respectively), we observed that increasing concentrations of SGK1-inh up to 30 μM were not able to reduce S6K signaling in these cellular models, as assessed by the downstream S6K targets pS6 (S235/6), pS6 (S240/4), and pmTOR (S2448) (Figure S5D). This suggests that SGK1-inh does not have activity toward S6K at concentrations below 20–30 μM. We also excluded any potential inhibition of mTORC1 by SGK1-inh by testing this compound against mTOR in a recombinant kinase assay (IC_50_ > 5,000 nM; Figure S5E).

Our computational analyses and preliminary characterization of SGK1-inh suggested that this compound acts as a type II kinase ATP-competitive inhibitor binding preferentially to the inactive conformation of the kinase (Figure 4D). By performing an ATP-competition assay we confirmed that addition of increasing concentrations of ATP decreased the potency of SGK1-inh in a dose-dependent manner (Figure S5F). Docking models using the active conformation of SGK1 show that the sulfonamide moiety with the terminal hydrophobic ring points out from the pocket toward the solvent (Figure S5G), rendering the bound state unstable. In contrast, in the inactive conformation several hydrophobic residues mediate interactions with SGK1-inh within the allosteric DFG-out pocket (mainly V149, L159, V154, and V160; Figure 4E). The pyrazolo(3,4-b)pyrazine head portion of SGK1-inh interacts with the key residues D177 and I179, similar to the interactions with ATP (Figures 4E and S5H). The energetics of SGK1-SGK1-inh binding are more favorable than SGK1-ATP, as assessed by binding free energy calculations (Figure S5I). The electrostatic components of these interactions are similar between ATP and SGK1-inh, and the majority of the binding energy arises from more favorable packing (van der Waals) interactions made between SGK1-inh and the kinase (Figure S5I). Most of the favorable interactions that take place between SGK1 and SGK1-inh are with amino acids found within the SGK1 active site (Figure S5J, upper panel). In silico alanine scanning of the key residues resulted in loss of binding free energies, confirming the importance of these amino acids in the protein-ligand interactions (Figure S5J, lower panel).

Based on the ability to inhibit NDRG1 phosphorylation in the presence of BYL719 in our models (Figure 4F), we estimated that the appropriate concentration of SGK1-inh to fully inhibit endogenous SGK1 is 10 μM. This relatively high concentration (still lower than the concentration needed to affect S6K activity) may be explained by the fact that these sulfonamide derivatives exhibit poor permeability (133 × 10^−7^ cm s^−1^ in Caco2 cell permeability assays) (Halland et al., 2015). Treatment of HCC1954 and JIMT1 cells with the combination of BYL719 and SGK1-inh not only abrogated pNDRG1 (T346) but also mTORC1 signaling (Figures 4G and S5K). Using m^7^GTP pulldowns we also found that combined PI3Kα and SGK1 inhibition induces a decreased cap-dependent translation, as seen by the increased 4EBP1 and decreased eIF4A and eIF4G binding to the m^7^GTP beads (Figure S5L). This translated to superior inhibition of cell viability of BYL719-resistant cell lines treated with the combination of BYL719 and SGK1-inh (Figures 4H, S5M, and S5N). We then assessed the potential antitumor activity of SGK1-inh in xenografts. We observed that only the combination of BYL719 and SGK1-inh reduced tumor burden (Figure 4I) and phosphorylation of S6, 4EBP1, and NDRG1 (Figures 4J and S5O).

These results show that targeting SGK1 pharmacologically is feasible, and demonstrate that dual inhibition of AKT and SGK1 is required to achieve full suppression of mTORC1 and proliferation.

### SGK1 Interacts with and Phosphorylates TSC2

**Figure 5:**
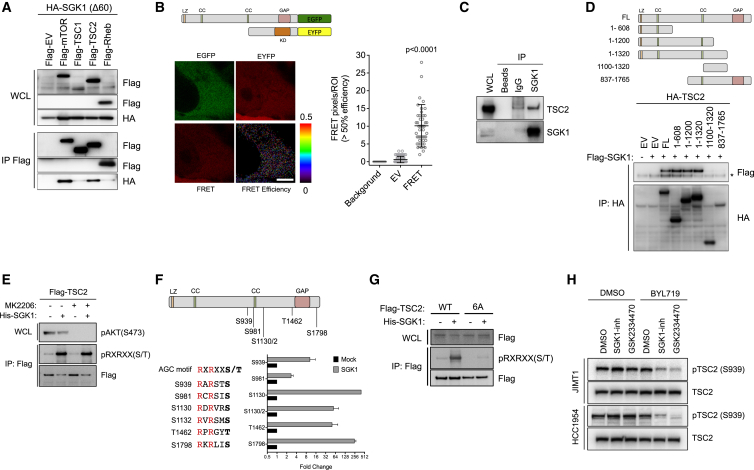
SGK1 Interacts with and Phosphorylates TSC2 (A) Co-immunoprecipitation assay in 293T cells ectopically express the indicated proteins. (B) Representative efficiency images from the FRET experiment performed in HeLa cells, with the constructs indicated above. Scale bar, 5 μm. Quantification of FRET efficiency dots is shown on the right. ROI, region of interest. (C) Co-immunoprecipitation (IP) of endogenous SGK1 and TSC2 in JIMT1 cells. IgG, immunoglobulin G. (D) Co-immunoprecipitation assay in 293T cells between FLAG-SGK1 and truncation mutants of HA-TSC2. Asterisk indicates IgG. Truncation TSC2 mutants are shown schematically at the top. Domains: LZ, leucine zipper; CC, coiled coil; GAP, GTPase activation protein. (E) In vitro kinase assay using recombinant His-SGK1 and immunoprecipitated FLAG-TSC2 from 293T cells as a substrate (2 μM MK2206, 1 hr). (F) Quantification of the phosphorylated site identified using liquid chromatography-MS/MS in the absence or presence of recombinant SGK1. Schematic view and amino acid sequence of the predicted SGK1 phosphorylation sites in TSC2 are shown at the top. (G) In vitro kinase assay using recombinant His-SGK1 and immunoprecipitated and dephosphorylated FLAG-TSC2 WT or 6A as a substrate. (H) Western blot of phosphorylated TSC2 (S939) in HCC1954 and JIMT1 cells treated with DMSO, BYL719 (1 μM), GSK2334470 (1 μM), SGK1-inh (10 μM), or the combination of both agents for 4 hr. p Values were calculated using Student's t test. Error bars denote ±SEM. See also Figure S6.

Due to its similarity with AKT, we reasoned that SGK1 could modulate mTORC1 activity by interacting with a component of the TSC/RHEB/mTORC1 axis. Immunoprecipitation of TSC1, TSC2, RHEB, and mTOR in 293T cells revealed that SGK1 physically interacts with both mTOR and TSC2 proteins (Figures 5A and S6A). While the interaction between SGK1 and mTOR has previously been described, as mTORC2 is responsible for the HM phosphorylation of SGK1 (Garcia-Martinez and Alessi, 2008), to our knowledge the interaction between SGK1 and TSC2 was previously unreported. This result was corroborated in a live cell context by performing fluorescence resonance energy transfer (FRET) experiments using EGFP-tagged TSC2 and EYFP-tagged SGK1 in HeLa cells (Figure 5B). We further confirmed the interaction between endogenous SGK1 and TSC2 by co-immunoprecipitation (Figure 5C). Moreover, we determined the proportion of endogenous SGK1 that is associated with the TSC complex by performing sucrose gradient experiments in JIMT1 lysates. The TSC complex fractionated at high-density fractions (fraction 5), as assessed by the presence of the three components TSC1, TSC2, and TBC1D7 (Figure S6B) (Dibble et al., 2012). Although most of the SGK1 fractionated at low-molarity fractions, approximately 20% of the kinase eluted at fractions similar to those of the TSC complex. Considering SGK1 as a monomer (or perhaps a dimer [Zhao et al., 2007]), only the association with a larger complex such as the TSC complex can explain the elution at these high sucrose gradients.

Co-immunoprecipitation assays using five different fragments of TSC2 demonstrated that SGK1 binds to the N-terminal region of TSC2 (Figure 5D), which contains a leucine zipper (LZ) domain important for protein-protein interactions and the interaction with TSC1 (Li et al., 2004).

SGK1 has high similarity to AKT in the kinase domain and thus shares many substrates that contain the AGC kinase consensus motif RXRXX(S/T), where R is arginine, X is any amino acid, and (S/T) is a phosphorylatable serine or threonine (Alessi et al., 2009). The use of a degenerated phosphospecific motif antibody allows detection of these phosphosites and has previously been shown to be a reliable surrogate for phospho-TSC2 detection (Manning et al., 2002). When we analyzed the TSC2 protein sequence we found seven putative sites of phosphorylation: S939, S981, T993, S1130, S1132, T1462, and S1798. All of these sites were conserved across several species (Figure S6C). We then established an in vitro kinase assay using recombinant active SGK1 and TSC2 immunoprecipitated from 293T cells as a substrate. To deplete endogenous phosphorylated TSC2, we pre-treated 293T cells with the AKT inhibitor MK2206. The addition of recombinant SGK1 kinase increased the phosphorylation of the RXRXX(S/T) sites of TSC2, even when cells were pre-treated with AKT inhibitor (Figure 5E). Using mass spectrometry (MS), we found increased phosphorylation in all of these sites, except for T993 (Figure 5F). Mutation of these six sites into the non-phosphorylatable amino acid alanine (TSC2 6A) completely abrogated the ability of SGK1 to phosphorylate TSC2 in vitro (Figure 5G).

It is well accepted that phosphorylation of these sites increases downstream RHEB-GTP loading and mTORC1 signaling, as a result of a translocation from the lysosome to the cytoplasm (Inoki et al., 2003a, Menon et al., 2014). The phosphorylation and inhibition of TSC2 is phenocopied by the loss of expression of the protein itself, as demonstrated by the induction of mTORC1 activity and consequent resistance to BYL719 in the T47D PI3Kα inhibitor-sensitive cell line depleted of TSC2 (Figures S6D and S6E). To confirm that our biochemical findings are consistent with the proposed mechanism of resistance to BYL719, we treated HCC1954 and JIMT1 cells with BYL719, GSK2334470, SGK1-inh, and the combination of these agents, and found that the phosphorylation of endogenous TSC2 decreases only upon dual PI3Kα and PDK1 or SGK1 suppression (Figure 5H). These results demonstrate that SGK1 can sustain mTORC1 activity in BYL719-resistant cells by phosphorylating and inhibiting the mTORC1-negative regulator TSC2.

We then asked whether kinases other than AKT or SGK1 are involved in the phosphorylation of TSC2 and sustained activation of mTORC1 upon PI3Kα inhibition (Figure S6F). Extracellular Signal-regulated Kinase (ERK) and the downstream AGC kinase RSK phosphorylate TSC2, activating downstream mTORC1 effectors (Ma et al., 2005, Roux et al., 2004). However, we did not detect changes in TSC2 phosphorylation at S939 (or mTORC1 downstream signaling) when HCC1954 cells were treated with the MEK inhibitors PD0325901 and GSK1120212, and downstream ERK and RSK were fully inhibited (Figure S6G). AMP-dependent protein kinase (AMPK), which is activated in conditions of energy stress, phosphorylates TSC2 at S1345 and induces the inhibition of mTORC1 (Inoki et al., 2003b). Treatment of HCC1954 cells with the stress-inducing agent 2-deoxyglucose and the AMPK activator A769662 were unable to rescue the sustained phosphorylation of S6 in this resistant model (Figure S6H). In line with the AMPK regulation of mTORC1 signaling, GSK3 kinase has also been reported to phosphorylate TSC2 using the AMPK-specific site S1345 as a priming event, in a process downstream of WNT signaling (Inoki et al., 2006). However, incubation of HCC1954 cells with the recombinant WNT antagonist DKK-1 did not reduce the sustained S6 phosphorylation (Figure S6I).

Altogether, these results suggest that in our resistant models SGK1 is the main kinase involved in the phosphorylation of TSC2 and sustained mTORC1 activation.

## Discussion

**Figure 6:**
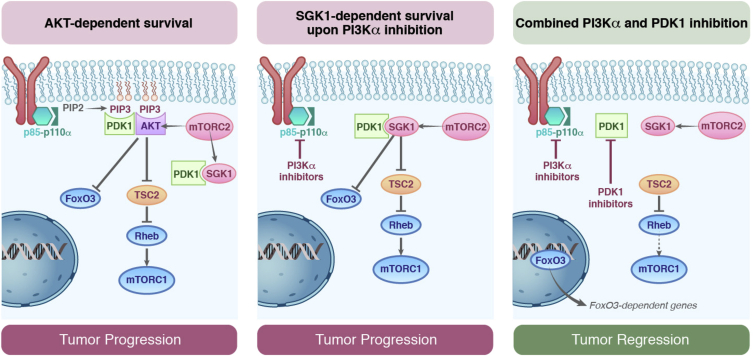
Proposed Model of PI3Kα Resistance in SGK1-Expressing Cells *PIK3CA*-mutant breast tumors depend on the PI3K pathway, which mainly signals through AKT. AKT phosphorylates and inhibits FOXO3 and TSC2, promoting mTORC1 activity and tumor progression (left panel). In the presence of PI3Kα inhibitors, PIP_3_ levels in the plasma membrane are negligible and AKT cannot be activated. High SGK1 cells become resistant to PI3Kα inhibitors, as SGK1 is not fully inhibited in the presence of these therapies, supporting FOXO3 and TSC2 phosphorylation, which promotes mTORC1 activity and tumor progression (middle panel). When SGK1 expressing cells are treated with PI3Kα and PDK1 inhibitors, both AKT and SGK1 are inhibited, inducing tumor regression as a result of FOXO3 activation and mTORC1 inhibition (right panel).

In this work, we show that inhibition of the constitutively active kinase PDK1 overcomes resistance to PI3Kα inhibitors in *PIK3CA*-mutant breast cancer cells insensitive to PI3Kα inhibition. We discovered that upon PI3Kα inhibition, and thus low levels of PIP_3_ and full suppression of AKT, SGK1 contributes to the maintenance of residual mTORC1 activity and cell survival through direct phosphorylation and inhibition of TSC2. Suppression of either PDK1 or SGK1 prevents mTORC1 activation and sensitizes resistant cells to PI3Kα blockade, underscoring the causative role of this signaling route in inducing the resistance phenotype (Figure 6).

Summarizing our current knowledge, resistance to PI3Kα inhibitors in *PIK3CA*-mutant malignancies may occur as a result of either PI3K-dependent or -independent mechanisms. An example of a PI3K-dependent acquired resistance mechanism has recently been shown by the observation that loss of PTEN results in activation of PI3K p110β, thus forfeiting PI3Kα signaling (Juric et al., 2015). Similarly, reactivation of PI3K p110β signaling has also been revealed to be a mechanism of adaptive resistance in PI3Kα-driven cells (Costa et al., 2015). In terms of PI3K-independent mechanisms, we now propose that mTORC1 sustained activity is, at least in part, mediated by PDK1-SGK1 signaling. In this context, AKT activity would be dispensable for cell survival, in accordance with previous reports showing that AKT activity is not always required for the downstream PI3K signaling (Gasser et al., 2014, Vasudevan et al., 2009).

The role of SGK1 in mediating mTORC1 activation upon PI3Kα inhibition can be explained by the differential regulation of AKT and SGK1 upon pharmacological stress. Although both kinases share the same upstream regulators, mTORC2 and PDK1, AKT contains a PH domain that is required for the PI3K-dependent plasma membrane translocation and subsequent activation. In contrast, SGK1 does not require plasma membrane localization, which could partially explain why it remains active in the absence of PIP_3_. In our resistant cell lines treated with PI3Kα inhibitor, we observe a substantial but incomplete decrease in SGK1 activity. This can be partially explained by the fact that PIP_3_ controls mTORC2 (Gan et al., 2011) in a mechanism that seems to require mSIN1 (Liu et al., 2015). However, other PIP_3_-independent pools of mTORC2 that are not regulated by growth factors (Frias et al., 2006) might be responsible for residual SGK1 activity. While PDK1 is a constitutively active kinase and can be present in both cytoplasm and membrane (upon PIP_3_ synthesis), the subcellular localization of mTORC2 is ambiguous (Cybulski and Hall, 2009). Therefore, it is plausible that different pools of mTORC2 can be found within the cell.

Pharmacological inhibition of PDK1 has been reported to have a profound effect on the activity of several AGC kinases such as RSK, S6K, PKC, and SGK (Najafov et al., 2011). However, higher doses of PDK1 inhibitors are required to achieve the same inhibitory effects on AKT. In fact, in the presence of 1 μM GSK2334470 AKT can be efficiently activated by PDK1 through different PIP_3_-dependent or PIF-binding pocket-dependent mechanisms (Najafov et al., 2012). In our experiments using endogenous immunoprecipitated SGK1 and AKT, we show that this is indeed the case. In the presence of BYL719, SGK1 but not AKT remains active; conversely, upon GSK2334470 treatment, SGK1 but not AKT is fully inhibited. Single activity of any of these kinases seems to be sufficient to propagate downstream pro-survival signaling through mTORC1 activation and FOXO3 repression. This is also confirmed by the fact that the combination of both agents efficiently inhibits FOXO3 and mTORC1, eliciting a powerful antitumor effect. In this setting, rather than inhibition of AKT, NDRG1 phosphorylation (a substrate of both AKT and SGK1) should be used as readout of pathway inhibition (Kobayashi et al., 1999).

The role of SGK1 in regulating signaling downstream of mTORC2 is intriguing but not entirely unexpected. From the evolutionary point of view, the SGK1 ortholog in *Drosophila melanogaster* is dAkt, which shares similarity with human SGK1 (63%) and AKT1 (67%). Thus, it is tempting to speculate that due to the high overlap of their substrates, *Drosophila* dAkt plays the role of both AKT and SGK1. In fact, Ypk2 and Gad8, the SGK1 orthologs in budding and fission yeast, respectively, are the main TORC2 downstream effectors (Cybulski and Hall, 2009, Kamada et al., 2005, Matsuo et al., 2003). Similarly, in *Caenorhabditis elegans*, sgk-1 appears to be essential for TORC2 signaling, lifespan, and growth (Jones et al., 2009, Soukas et al., 2009).

In summary, our findings show that SGK1 mediates resistance to PI3Kα inhibitors through the activation of mTORC1, which can be reverted by PDK1 blockade. This study highlights the importance of understanding the underlying mechanisms of protein kinase regulation in uncovering critical nodes for pharmacological intervention and improving the therapeutic options for oncogene-driven cancers.

## Experimental Procedures

### RNAi Screening

JIMT1 and HCC1954 cells were seeded and reverse transfected using Dharmafect-1 with the kinome and phosphatome Ambion Silencer Select v4.0 libraries. Cells were treated with DMSO or 1 μM BYL719, and 7 days after transfection cell viability was assessed. Full details can be found in Supplemental Experimental Procedures.

### Immunoblot Detection

Protein lysates were extracted and separated using SDS-PAGE gels according to standard methods. Membranes were probed using specific antibodies. PDK1, pAKT (S473), pAKT (T308), pS6K (T389), pS6 (S240/4), pS6 (235/6), p4EBP1 (S65), PARP, Actin, pRSK (S227), cleaved caspase 3, pFOXO1/3 (T24/T32), SGK1, SGK2, SGK3, pNDRG1 (T346), NDRG1, FLAG, HA, and phospho-RXRXX(S/T) were from Cell Signaling Technology.

### Animal Studies

Animals were housed, maintained, and treated at Memorial Sloan Kettering Cancer Center (MSKCC) in accordance with Institutional Animal Care and Use Committee (protocol number 12-10-019). 5 × 10^6^ cells in 1:1 PBS/Matrigel (Corning) were injected subcutaneously into athymic *Foxn1*^*nu*^ nude mice. When a volume of ∼150 mm^3^ was reached mice were randomized and treated, and tumors were measured twice per week for 1 month. Full details can be found in Supplemental Experimental Procedures.

### Patient Samples

The MSKCC Institutional Review Board approved the study. Pre-treatment formalin-fixed paraffin-embedded blocks from patients treated with the PI3Kα inhibitor BYL719 enrolled in the clinical trial NCT01870505 conducted at MSKCC were used for immunohistochemistry (IHC). Informed consent was obtained from all subjects. Full details of tissue microarray construction are provided in Supplemental Experimental Procedures.

## Author Contributions

P.C., D.R.A., J.B., and M.S. designed the research. P.C. and H.E. performed the in vitro and in vivo experiments. R.B. performed endogenous kinase assays. E.T. performed ChIP experiments. S.K. and C.S.V. performed the docking and MS. F.J.C. performed methylation analysis. E.B. scored and quantified IHC experiments. P.R., S. C., and M.D. provided patient samples. P.C. prepared the figures. P.C., M.S., and J.B. wrote the manuscript.

## Accession Numbers

The microarray data have been deposited in the Gene Expression Omnibus database under accession number GEO: GSE69189.

## Supplemental Information

Document S1. Supplemental Experimental Procedures, Figures S1–S6, and Table S1

Document S2. Article plus Supplemental Information
