# Supplementary material for: PDK1-SGK1 Signaling Sustains AKT-Independent mTORC1 Activation and Confers Resistance to PI3Kα Inhibition
Source: Cancer Cell. 2016 Aug 8;30(2):229–42. doi: 10.1016/j.ccell.2016.06.004 (PMC4982440; doi:10.1016/j.ccell.2016.06.004)
Supplement: Document S1. Supplemental Experimental Procedures, Figures S1–S6, and Table S1 [file mmc1.pdf]

Cancer Cell, Volume *30*

Supplemental Information

PDK1-SGK1 Signaling Sustains AKT-Independent mTORC1 Activation and Confers Resistance to PI3Kα Inhibition

Pau Castel, Haley Ellis, Ruzica Bago, Eneda Toska, Pedram Razavi, F. Javier Carmona, Srinivasaraghavan Kannan, Chandra S. Verma, Maura Dickler, Sarat Chandarlapaty, Edi Brogi, Dario R. Alessi, José Baselga, and Maurizio Scaltriti

Supplemental Data

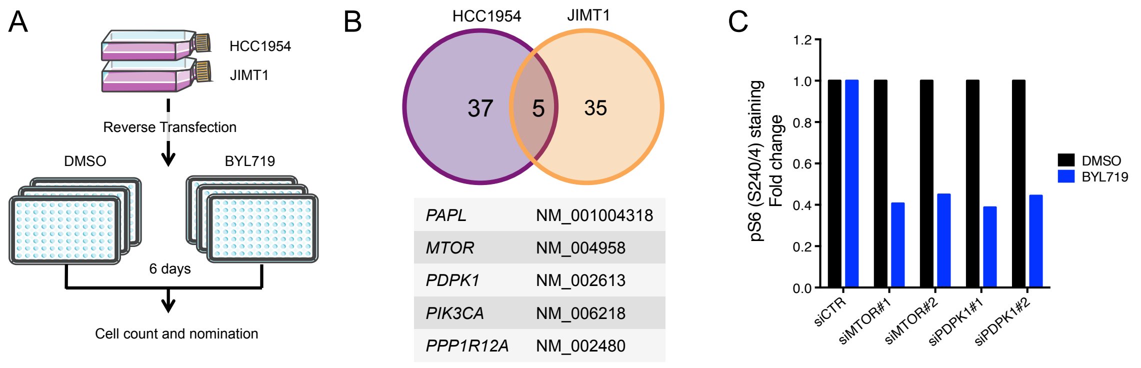

Figure S1. Relative to Figure 1

(A) Overview of the large screening carried out in this work using libraries against the human kinome and phosphatome. **(B)** Venn diagram indicating the number of genes found to sensitize to BYL719 treatment (1 μM) in each cell line and in common. The table contains the gene name and NCBI mRNA accession number targeted by the siRNA found to sensitize both cell lines to BYL719. **(C)** Quantification of pS6 (S240/4) staining in the siCTR, siMTOR, and siPDK1 transfected cells in the presence of DMSO or BYL719 (1 μM) in JIMT1 cells. Quantification of the green fluorescence from the whole well is indicated as a fold change of the control untreated cells.

Table S1. Relative to Figure 1. Genes identified in the siRNA screening

HCC1954 cell line

Gene Symbol	RefSeq ID	Gene Description	H score (Drug)	Validated viability	pS6 (S240/4) staining
AKAP8	NM_005858	A kinase (PRKA) anchor protein 8	67		
CAMKK2	NM_006549	calcium/calmodulin-dependent protein kinase kinase 2, beta	67		
CHEK1	NM_001274	CHK1 checkpoint homolog	67		
DUSP10	NM_007207	dual specificity phosphatase 10	67		
ERBB3	NM_001005915	v-erb-b2 erythroblastic leukemia viral oncogene homolog 3	67		
FBP1	NM_000507	fructose-1,6-bisphosphatase 1	67		
FER	NM_005246	fer (fps/fes related) tyrosine kinase	67		
PAPL	NM_001004318	purple acid phosphatase long form	67	NO	NO
FLT1	NM_002019	fms-related tyrosine kinase 1 (vascular endothelial growth factor)	67		
MTOR	NM_004958	FK506 binding protein 12-rapamycin associated protein 1	67	**YES**	**YES**
HDHD1A	NM_012080	haloacid dehalogenase-like hydrolase domain containing 1A	67		
INPPL1	NM_001567	inositol polyphosphate phosphatase-like 1	100		
IRAK4	NM_016123	interleukin-1 receptor-associated kinase 4	67		
JAK1	NM_002227	Janus kinase 1	67		
MAP2K3	NM_002756	mitogen-activated protein kinase kinase 3	100		
MASTL	NM_032844	microtubule associated serine/threonine kinase-like	67		
MPP5	NM_022474	membrane protein, palmitoylated 5	67		
MPP7	NM_173496	membrane protein, palmitoylated 7	67		
MTMR1	NM_003828	myotubularin related protein 1	100		
MTMR3	NM_021090	myotubularin related protein 3	67		
MTMR6	NM_004685	myotubularin related protein 6	67		
MTMR8	NM_017677	myotubularin related protein 8	67		
NAP1L1	NM_004537	nucleosome assembly protein 1-like 1	100		
NAP1L4	NM_005969	nucleosome assembly protein 1-like 4	67		
NME4	NM_005009	non-metastatic cells 4	67		
PDGFRA	NM_006206	platelet-derived growth factor receptor, alpha polypeptide	67		
PDPK1	NM_002613	3-phosphoinositide dependent protein kinase-1	100	**YES**	**YES**
PIK3CA	NM_006218	phosphoinositide-3-kinase	100	NO	NO
PIP5KL1	NM_173492	phosphatidylinositol-4-phosphate 5-kinase-like 1	67		
PLK3	NM_004073	polo-like kinase 3	100		
PPAP2B	NM_003713	phosphatidic acid phosphatase type 2B	67		
PPP1R12A	NM_002480	protein phosphatase 1, regulatory (inhibitor) subunit 12A	67	NO	NO
PPP1R12B	NM_032104	protein phosphatase 1, regulatory (inhibitor) subunit 12B	100		
PPP1R8	NM_002713	protein phosphatase 1, regulatory (inhibitor) subunit 8	67		
PPP6C	NM_002721	protein phosphatase 6, catalytic subunit	67		
PTP4A1	NM_003463	protein tyrosine phosphatase type IVA	67		
PTPN13	NM_006264	protein tyrosine phosphatase, non-receptor type 13	67		
PXK	NM_017771	PX domain containing serine/threonine kinase	67		
RBKS	NM_022128	ribokinas	67		
SNRK	NM_017719	SNF related kinase	67		
TNK1	NM_003985	tyrosine kinase, non-receptor	67		
WNK2	NM_006648	WNK lysine deficient protein kinase 2	67		

JIMT1 cell line

Gene Symbol	RefSeq ID	Gene Description	H score (Drug)	Validated viability	pS6 (S240/4) staining
ACVRL1	NM_000020	activin A receptor type II-like 1	67		
AKT1	NM_001014431	v-akt murine thymoma viral oncogene homolog 1	67		
ALPI	NM_001631	alkaline phosphatase, intestinal	67		
ALPL	NM_000478	alkaline phosphatase, liver/bone/kidney	67		
ARAF	NM_001654	v-raf murine sarcoma 3611 viral oncogene homolog	67		
CDC42BPA	NM_003607	CDC42 binding protein kinase alpha	67		
CDK2	NM_001798	cyclin-dependent kinase 2	67		
CDK4	NM_000075	cyclin-dependent kinase 4	100		
CDK9	NM_001261	cyclin-dependent kinase 9 (CDK9), mRNA	67		
CSNK1A1	NM_001025105	casein kinase 1, alpha 1	67		
CSNK1A1L	NM_145203	casein kinase 1, alpha 1-like	67		
CSNK1G2	NM_001319	casein kinase 1, gamma 2	67		
ENTPD2	NM_001246	ectonucleoside triphosphate diphosphohydrolase 2	67		
EPHA3	NM_005233	EPH receptor A3 (EPHA3), transcript variant 1, mRNA	67		
EPHA5	NM_004439	EPH receptor A5	67		
EPHB2	NM_004442	EPH receptor B2	67		
PAPL	NM_001004318	purple acid phosphatase long form	67	NO	NO
MTOR	NM_004958	FK506 binding protein 12-rapamycin associated protein 1	67	**YES**	**YES**
GK5	NM_001039547	glycerol kinase 5	67		
IGFN1	NM_178275	immunoglobulin-like and fibronectin type III domain containing 1	67		
ITGB1BP3	NM_170678	integrin beta 1 binding protein 3	67		
LRRK2	NM_198578	leucine-rich repeat kinase 2	67		
LTK	NM_002344	leukocyte receptor tyrosine kinase	67		
MAP2K2	NM_030662	mitogen-activated protein kinase kinase	67		
MST1R	NM_002447	macrophage stimulating 1 receptor	67		
NEK3	NM_002498	NIMA (never in mitosis gene a)-related kinase 3	67		
NEK7	NM_133494	NIMA (never in mitosis gene a)-related kinase 7	67		
PDK2	NM_002611	pyruvate dehydrogenase kinase, isozyme 2	67		
PDPK1	NM_002613	3-phosphoinositide dependent protein kinase-1	67	**YES**	**YES**
PIK3CA	NM_006218	phosphoinositide-3-kinase, catalytic, alpha polypeptide	67, 100	NO	NO
PPP1CA	NM_001008709	protein phosphatase 1, catalytic subunit, alpha isoform	67		
PPP1R12A	NM_002480	protein phosphatase 1, regulatory (inhibitor) subunit 12A	67	NO	NO
PPP1R3F	NM_033215	protein phosphatase 1, regulatory (inhibitor) subunit 3F	67		
PPP2R5D	NM_006245	protein phosphatase 2, regulatory subunit B', delta isoform	67		
PRKD2	NM_001079880	protein kinase D2	67		
PTPRF	NM_002840	protein tyrosine phosphatase, receptor type, F	67		
PTPRT	NM_007050	protein tyrosine phosphatase, receptor type, T	67		
TESK1	NM_006285	testis-specific kinase 1	67		
TRIB1	NM_025195	tribbles homolog 1	67		
WNK1	NM_018979	WNK lysine deficient protein kinase 1	67		

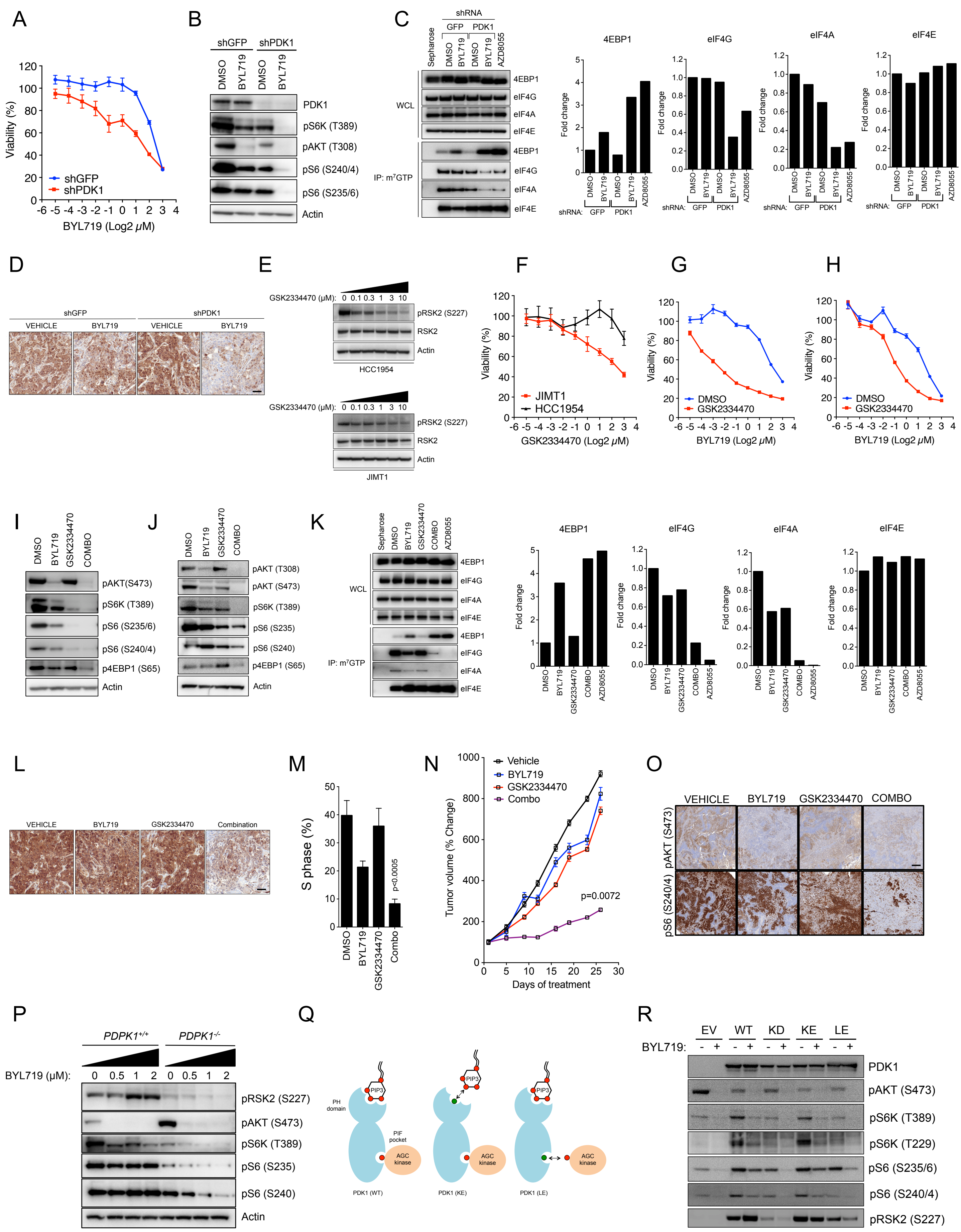
A
120
100
80
60
40
20
0
Viability (%)
-6 -5 -4 -3 -2 -1 0 1 2 3 4
BYL719 (Log2 μM)
shGFP
shPDK1
B
shGFP
shPDK1
DMSO
BYL719
DMSO
BYL719
PDK1
pS6K (T389)
pAKT (T308)
pS6 (S240/4)
pS6 (S235/6)
Actin
C
shRNA
GFP
PDK1
Sepharose
DMSO
BYL719
DMSO
BYL719
AZD8055
WCL
IP: m7GTP
4EBP1
eIF4G
eIF4A
eIF4E
4EBP1
eIF4G
eIF4A
eIF4E
Fold change
shRNA: GFP PDK1
D
shGFP
shPDK1
VEHICLE
BYL719
VEHICLE
BYL719
E
GSK2334470 (μM):
0 0.1 0.3 1 3 10
pRSK2 (S227)
RSK2
Actin
HCC1954
JIMT1
F
Viability (%)
JIMT1
HCC1954
GSK2334470 (Log2 μM)
G
DMSO
GSK2334470
BYL719 (Log2 μM)
H
DMSO
GSK2334470
BYL719 (Log2 μM)
I
DMSO
BYL719
GSK2334470
COMBO
pAKT(S473)
pS6K (T389)
pS6 (S235/6)
pS6 (S240/4)
p4EBP1 (S65)
Actin
J
DMSO
BYL719
GSK2334470
COMBO
pAKT (T308)
pAKT (S473)
pS6K (T389)
pS6 (S235)
pS6 (S240)
p4EBP1 (S65)
Actin
K
Sepharose
DMSO
BYL719
GSK2334470
COMBO
AZD8055
WCL
IP: m7GTP
4EBP1
eIF4G
eIF4A
eIF4E
Fold change
L
VEHICLE
BYL719
GSK2334470
Combination
M
S phase (%)
DMSO
BYL719
GSK2334470
Combo
p<0.0005
N
Vehicle
BYL719
GSK2334470
Combo
Tumor volume (% Change)
Days of treatment
p=0.0072
O
VEHICLE
BYL719
GSK2334470
COMBO
pAKT (S473)
pS6 (S240/4)
P
PDPK1+/+
PDPK1-/-
BYL719 (μM):
0 0.5 1 2 0 0.5 1 2
pRSK2 (S227)
pAKT (S473)
pS6K (T389)
pS6 (S235)
pS6 (S240)
Actin
Q
PIP3
PH domain
PIF pocket
AGC kinase
PDK1 (WT)
PDK1 (KE)
PDK1 (LE)
R
EV
WT
KD
KE
LE
BYL719:
PDK1
pAKT (S473)
pS6K (T389)
pS6K (T229)
pS6 (S235/6)
pS6 (S240/4)
pRSK2 (S227)

Figure S2. Relative to Figure 1

(A) Western blot comparing JIMT1 shGFP and shPDK1 cells treated with BYL719 (1 μM) for 4 hr. **(B)** Dose-response curves from JIMT1 shGFP and shPDK1 cells treated with BYL719 for 6 days. **(C)** m^7GTP pull down assay for HCC1954 shGFP and shPDK1 cells treated with BYL719 (1 μM) for 4 hr. Quantification of the m^7GTP-precipitated proteins is indicated in fold change. AZD8055 is used as a control at 1 μM. **(D)** p4EBP1 (T37/46) IHC from the tumors harvested from Figure 1E. Scale bar: 100 μM. **(E)** Western blot analysis of phosphorylated RSK2 (S227) in HCC1954 and JIMT1 cells treated with increasing concentrations of GSK2334470 for 8 hr. **(F)** Dose-response curves from HCC1954 and JIMT1 resistant cell lines treated with GSK2334470 for 6 days. **(G)** Dose-response curves from JIMT1 cells treated with BYL719 in the presence or absence of GSK2334470 (1 μM) during 6 days. **(H)** Same as (G) using BT20 TNBC cells. **(I)** Western blot comparing JIMT1 cells treated with BYL719 (1 μM), GSK2334470 (1 μM), or the combination of both agents for 4 hr. **(J)** Same as (I) using BT20 TNBC cells. **(K)** m^7GTP pull down assay for HCC1954 cells treated with BYL719 (1 μM), GSK2334470 (1 μM), or the combination of both agents for 4 hr. Quantification of the m^7GTP-precipitated proteins is indicated in fold change. AZD8055 is used as a control at 1 μM. **(L)** p4EBP1 (T37/46) IHC from the tumors harvested from Figure 1K. Scale bar: 100 μM. **(M)** S-phase quantification in JIMT1 cells treated with BYL719 (1 μM), GSK2334470 (1 μM), or the combination of both agents for 24 hr and stained with Propidium iodide for cell cycle analysis. **(N)** JIMT1 in vivo xenograft treated with Vehicle, BYL719, GSK2334470, or the combination of both agents (n=10/arm). **(O)** IHC analysis of tumors from (N) collected at the end of the experiment 4 hr after the last dosage. Scale bar: 100 μM. **(P)** Western blot analysis of HCT116 *PDPK1*+/+ and *PDPK1*-/- isogenic cell lines treated with increasing concentrations of BYL719 for 4 hr. **(Q)** Schematic representation of the effects of the PIP3-binding and PIF-binding pocket deficient mutants used in (R). Red circles indicate phosphate groups and green circles indicate hydrophilic-charged aminoacid E. Arrows indicate electric charge repulsion. **(R)** Western blot of HCT116 *PDPK1*-/- cells transfected with different pCCL-PDK1 mutants. EV (empty vector), WT (wild type), KD (kinase death; K111N), KE (PIP3-binding deficient; K465E), LE (PIF-binding pocket deficient; L155E). Cells were treated with BYL719 (1 μM) for 4 hr before collection.

p values are calculated using Student's t-test. Error bars are ± SEM.

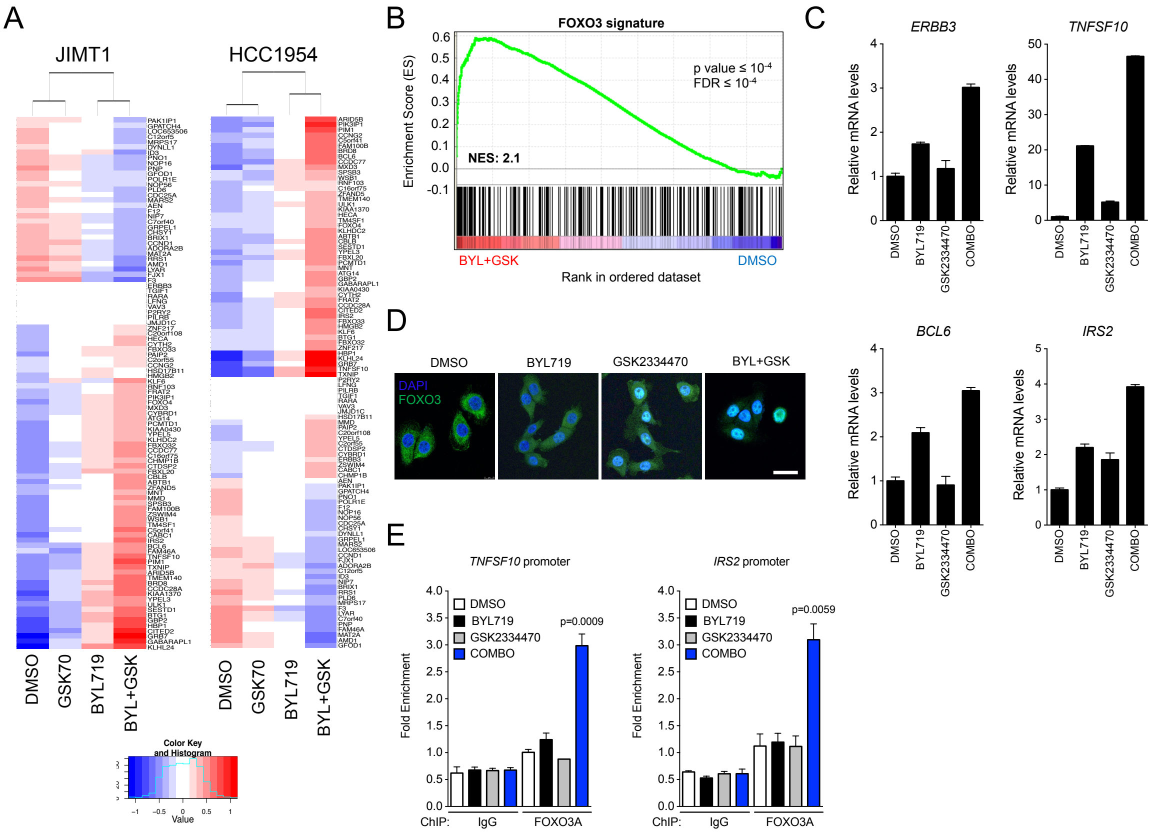

Figure S3. Relative to Figure 2

(A) Differentially expressed genes in JIMT1 (left) and HCC1954 (right) cells treated with BYL719 (1 µM), GSK2334470 (1 µM), or the combination of both agents for 4 hr. Gene expression up-regulation is indicated in red, while blue represents gene expression down-regulation. **(B)** Enrichment plot for the FOXO3 signature of GSEA in JIMT1 cells. NES: Normalized Enrichment Score. **(C)** *ERBB3*, *TNFSF10*, *BCL6*, and *IRS2* mRNA expression in JIMT1 cells treated with DMSO, BYL719 (1 µM), GSK2334470 (1 µM), or the combination of both agents for 4 hr. **(D)** FOXO3A immunofluorescence (green) in JIMT1 cells treated with DMSO, BYL719 (1 µM), GSK2334470 (1 µM), or the combination of both agents for 4 hr. Nuclei are shown in blue (DAPI). Scale bar: 25 µM. **(E)** ChIP-qPCR assay of FOXO3A binding at the *TNFSF10A* and *IRS2* promoters in JIMT1 cells treated as indicated in (C).

p values are calculated using Student's t-test. Error bars are ± SEM.

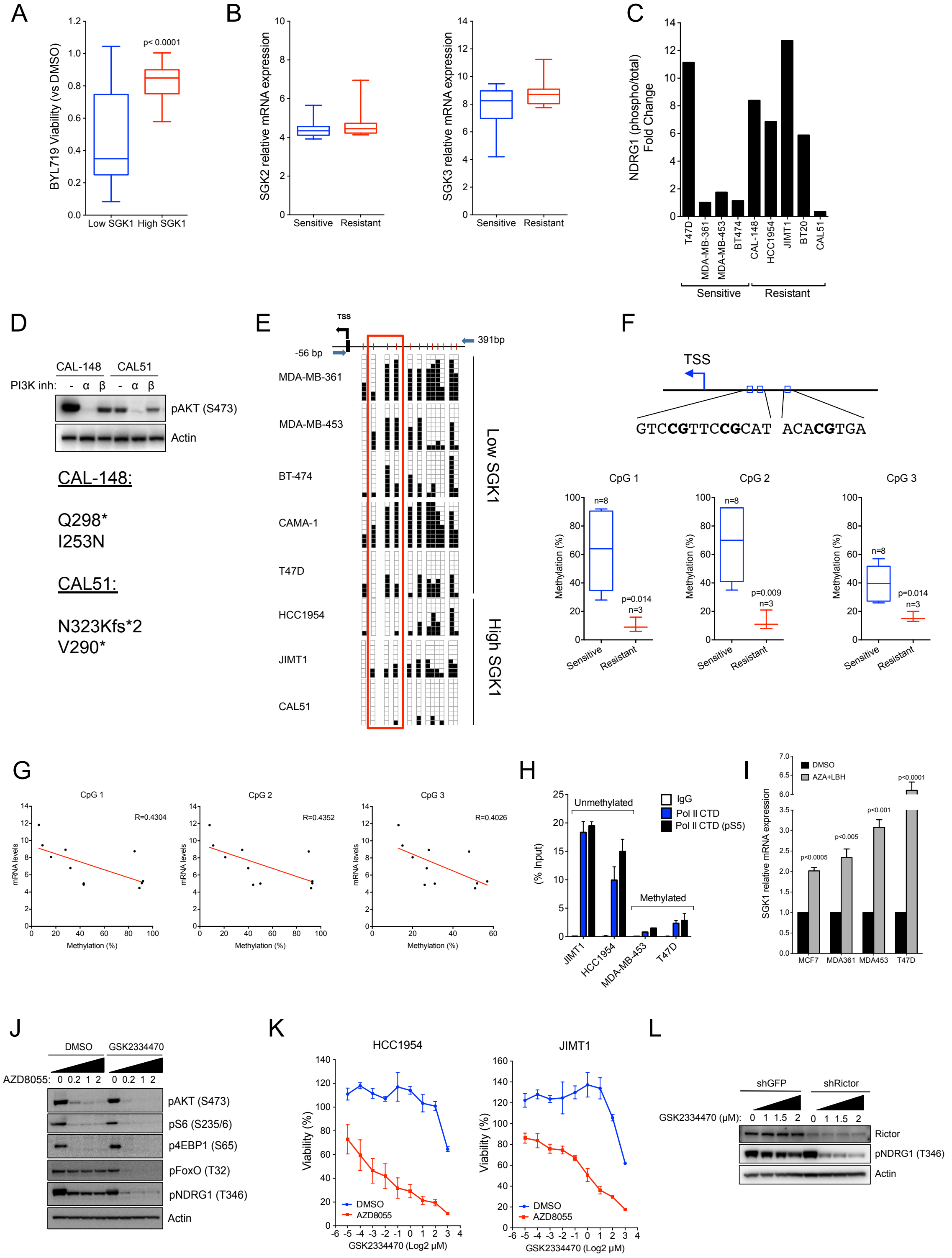
A
BYL719 Viability (vs DMSO)
p< 0.0001
Low SGK1
High SGK1
B
SGK2 relative mRNA expression
Sensitive
Resistant
SGK3 relative mRNA expression
Sensitive
Resistant
C
NDRG1 (phospho/total) Fold Change
T47D
MDA-MB-361
MDA-MB-453
BT474
CAL-148
HCC1954
JIMT1
BT20
CAL51
Sensitive
Resistant
D
CAL-148
CAL51
PI3K inh:
- α β - α β
pAKT (S473)
Actin
CAL-148:
Q298*
I253N
CAL51:
N323Kfs*2
V290*
E
TSS
-56 bp
391bp
MDA-MB-361
MDA-MB-453
BT-474
CAMA-1
T47D
HCC1954
JIMT1
CAL51
Low SGK1
High SGK1
F
TSS
GTCCGTTCCGCAT ACACGTGA
CpG 1
CpG 2
CpG 3
Methylation (%)
n=8
n=3
p=0.014
p=0.009
p=0.014
Sensitive
Resistant
G
CpG 1
CpG 2
CpG 3
R=0.4304
R=0.4352
R=0.4026
mRNA levels
Methylation (%)
H
Unmethylated
Methylated
IgG
Pol II CTD
Pol II CTD (pS5)
(% Input)
JIMT1
HCC1954
MDA-MB-453
T47D
I
DMSO
AZA+LBH
SGK1 relative mRNA expression
p<0.0005
p<0.005
p<0.001
p<0.0001
MCF7
MDA361
MDA453
T47D
J
DMSO
GSK2334470
AZD8055:
0 0.2 1 2 0 0.2 1 2
pAKT (S473)
pS6 (S235/6)
p4EBP1 (S65)
pFoxO (T32)
pNDRG1 (T346)
Actin
K
HCC1954
JIMT1
Viability (%)
DMSO
AZD8055
GSK2334470 (Log2 µM)
L
shGFP
shRictor
GSK2334470 (µM):
0 1 1.5 2 0 1 1.5 2
Rictor
pNDRG1 (T346)
Actin

Figure S4. Relative to Figure 3

(A) Cell viability of breast cancer cell lines treated with BYL719 (2 µM) and classified according to the SGK1 mRNA expression in high (>median expression) and low (<median expression) (n=27). Box indicates the median and the interquartile range, while whiskers represent minimum and maximum. **(B)** SGK2 and SGK3 mRNA levels in breast cancer cell lines sensitive or resistant to BYL719 (n=27). Box indicates the median and the interquartile range, while whiskers represent minimum and maximum. **(C)** Quantification of pNDRG1 (T346) basal levels in *PIK3CA*-mutant breast cancer cell lines classified according to their sensitivity to BYL719. **(D)** pAKT (S473) Western blot in CAL-148 and CAL51 cells treated with BYL719 (1 µM) and AZD6482 (1 µM) during 4 hr. Mutations identified in *PTEN* are shown below. **(E)** Bisulfite sequencing of the promoter region of *SGK1* in a cohort of eight breast cancer cell lines classified according to their sensitivity to BYL719. In red, the three CpG sites identified to be differentially methylated. TSS: Transcription Start Site. **(F)** Schematic representation of the three CpG sites (bold) identified to be differentially methylated in the promoter of *SGK1*. Below, pyrosequencing quantification of the methylated CpG sites in eleven breast cancer cell lines classified according to their sensitivity to BYL719. Box indicates the median and the interquartile range, while whiskers represent minimum and maximum. **(G)** Correlation between the SGK1 mRNA levels and the percentage of CpG promoter methylation in the cells indicated in (F). R indicates the R-square goodness of fit, and all correlations had a significant p value <0.05. **(H)** ChIP-qPCR assay of RNA Polymerase II (Pol II) and the phosphorylated S5 of RNA Polymerase II (Pol II pS5) for *SGK1* promoter in unmethylated (resistant) and methylated (sensitive) cell lines. Primers for the *SGK1* promoter were design in order to amplify the region containing the three CpG islands identified in this study. CTD: C-terminal domain. **(I)** RT-qPCR analysis of SGK1 mRNA levels in methylated sensitive cell lines treated for 72 hr with the demethylating agent 5-aza-2'-deoxycytidine(5 µM) and the histone deacetylase inhibitor panobinostat (LBH) (50 nM). **(J)** Western blot analysis of HCC1954 cells treated with increasing concentrations of the mTOR catalytic inhibitor AZD8055 (µM) in the presence or absence of GSK2334470 (1 µM) for 4 hr. **(K)** Dose-response curves of HCC1954 and JIMT1 cell lines treated with increasing concentrations of GSK2334470 in the presence or absence of the mTOR catalytic inhibitor AZD8055 (1 µM) for 6 days. **(L)** Western blot analysis of JIMT1 cells stably expressing shRNA against GFP and RICTOR mRNA and treated with increasing concentrations of GSK2334470.

p values are calculated using Student's t-test. Error bars are ± SEM.

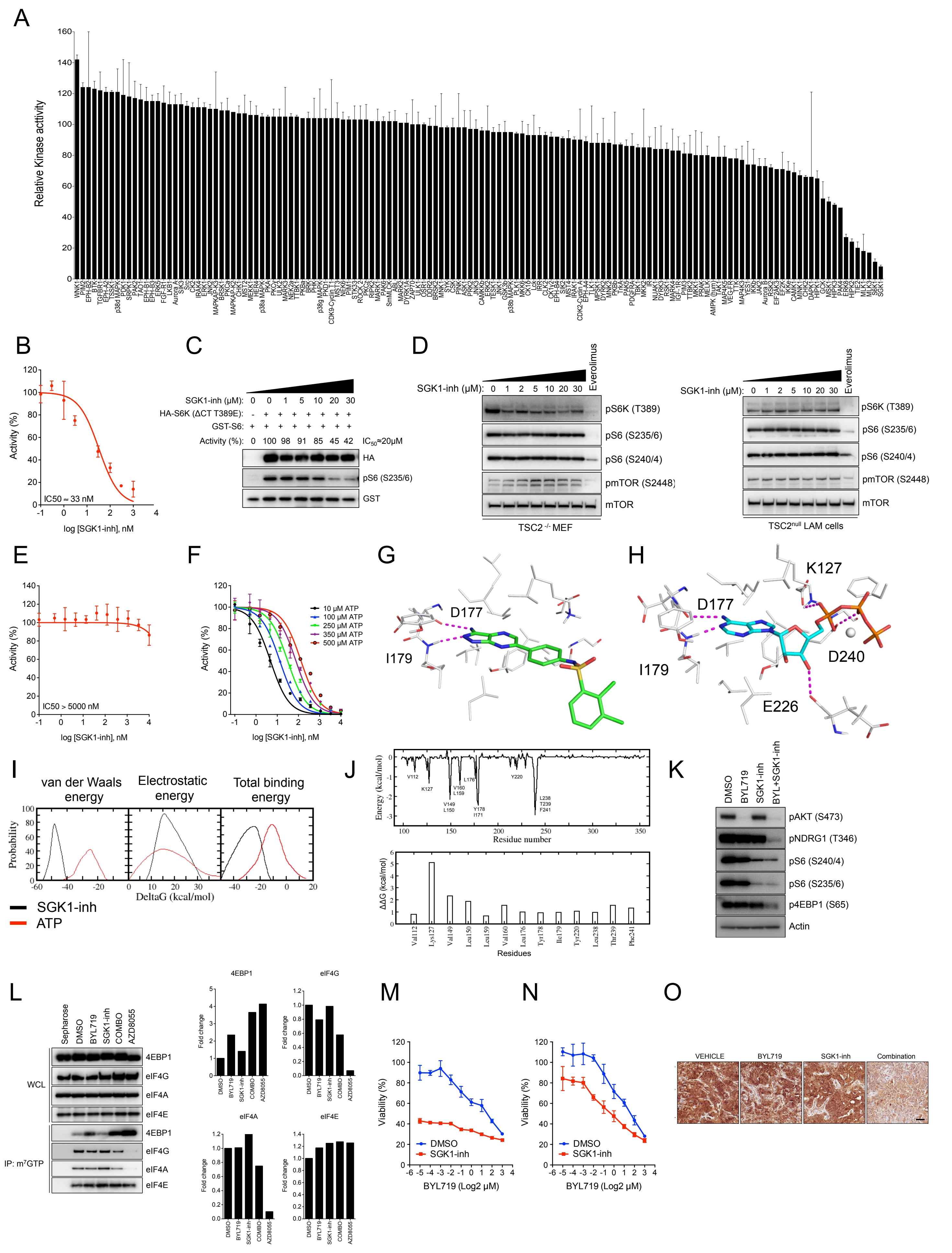
A
Relative Kinase activity
B
Activity (%)
IC50 ≈ 33 nM
log [SGK1-inh], nM
C
SGK1-inh (µM): 0 0 1 5 10 20 30
HA-S6K (ΔCT T389E): - + + + + + +
GST-S6: + + + + + + +
Activity (%): 0 100 98 91 85 45 42
IC50≈20µM
HA
pS6 (S235/6)
GST
D
SGK1-inh (µM): 0 1 2 5 10 20 30
Everolimus
pS6K (T389)
pS6 (S235/6)
pS6 (S240/4)
pmTOR (S2448)
mTOR
TSC2 -/- MEF
TSC2null LAM cells
E
IC50 > 5000 nM
F
10 µM ATP
100 µM ATP
250 µM ATP
350 µM ATP
500 µM ATP
G
D177
I179
H
K127
D177
I179
D240
E226
I
van der Waals energy
Electrostatic energy
Total binding energy
Probability
DeltaG (kcal/mol)
SGK1-inh
ATP
J
Energy (kcal/mol)
Residue number
ΔΔG (kcal/mol)
Residues
K
DMSO
BYL719
SGK1-inh
BYL+SGK1-inh
pAKT (S473)
pNDRG1 (T346)
pS6 (S240/4)
pS6 (S235/6)
p4EBP1 (S65)
Actin
L
Sepharose
DMSO
BYL719
SGK1-inh
COMBO
AZD8055
WCL
IP: m7GTP
4EBP1
eIF4G
eIF4A
eIF4E
Fold change
M
N
Viability (%)
DMSO
SGK1-inh
BYL719 (Log2 µM)
O
VEHICLE
BYL719
SGK1-inh
Combination

Figure S5. Relative to Figure 4

(A) Selectivity screening results of SGK1-inh at 1 μM against a library containing 140 kinases representative of the human kinome. Values are available at the Kinase Inhibitor Database of the MRC Protein Phosphorylation and Ubiquitylation Unit of the University of Dundee (http://www.kinasescreen.mrc.ac.uk/kinase-inhibitors). **(B)** In vitro S6K1 kinase assay using recombinant KKRNRTLTK peptide as a substrate in the presence of increasing concentrations of SGK1-inh. IC_{50} value is indicated. **(C)** In vitro S6K1 kinase assay using constitutively active S6K kinase immunoprecipitated from 293T cells expressing HA-S6K (ΔCT T389E) and treated with increasing concentrations of SGK1-inh. Recombinant GST-S6 was used as a substrate and phosphorylated S6 (S235/6) antibody was used for the detection of phosphorylated substrate by Western blot. IC_{50} value is indicated. **(D)** Western blot analysis of S6K targets in *TSC2* knockout mouse embryonic fibroblasts (MEF) and fibroblasts derived from a *TSC2*^{null} Lymphangioleiomyomatosis (LAM) patient treated with increasing concentrations of SGK1-inh for 4 hr. Everolimus was used as a positive control at 200 nM. **(E)** In vitro mTOR kinase assay using recombinant 4EBP1 as a substrate in the presence of increasing concentrations of SGK1-inh. IC_{50} value is indicated. **(F)** SGK1-inh IC_{50} determination in an ATP competition assay using increasing concentration of ATP. **(G)** Residues involved in the interaction between SGK1 (active conformation) and SGK1-inh. Hydrogen bonds are shown as purple dotted lines. **(H)** Residues involved in the interaction between ATP and the active conformation of SGK1. Hydrogen bonds are shown as purple dotted lines and Mg^{+2} as a grey sphere. **(I)** Distribution of free energies (ΔG) of the conformations sampled during MD simulations of SGK1 bound to SGK1-inh (black) or ATP (red). Distribution of van der Waals' interactions and electrostatic solvation contribution for the total binding energy are shown. **(J)** Upper panel. Decomposition of binding free energy on per-residue basis for SGK1 (DFG-out conformation) and SGK1-inh complex. Lower panel. Alanine scanning results for the selected residues are shown. Results are expressed in the change of free energy when the indicated residues are mutated to alanine ΔΔG. **(K)** Western blot analysis of JIMT1 cells treated with BYL719 (1 μM), SGK1-inh (10 μM), or the combination of both agents for 4 hr. **(L)** m^7GTP pull down assay for HCC1954 cells treated with BYL719 (1 μM), SGK1-inh (10 μM), or the combination of both agents for 4 hr. Quantification of the m^7GTP-precipitated proteins is indicated in fold change. AZD8055 is used as a control at 1 μM. **(M)** Dose-response curves from JIMT1 cells treated with increasing concentrations of BYL719 in the presence or absence of SGK1-inh (2 μM) for 6 days. **(N)** Dose response curves from JIMT1 cells treated with increasing concentrations of BYL719 in the presence or absence of SGK1-inh (2 μM) for 6 days. **(O)** p4EBP1 (T37/46) IHC from the tumors harvested from Figure 4L. Scale bar: 100 μM.

Error bars are ± SEM.

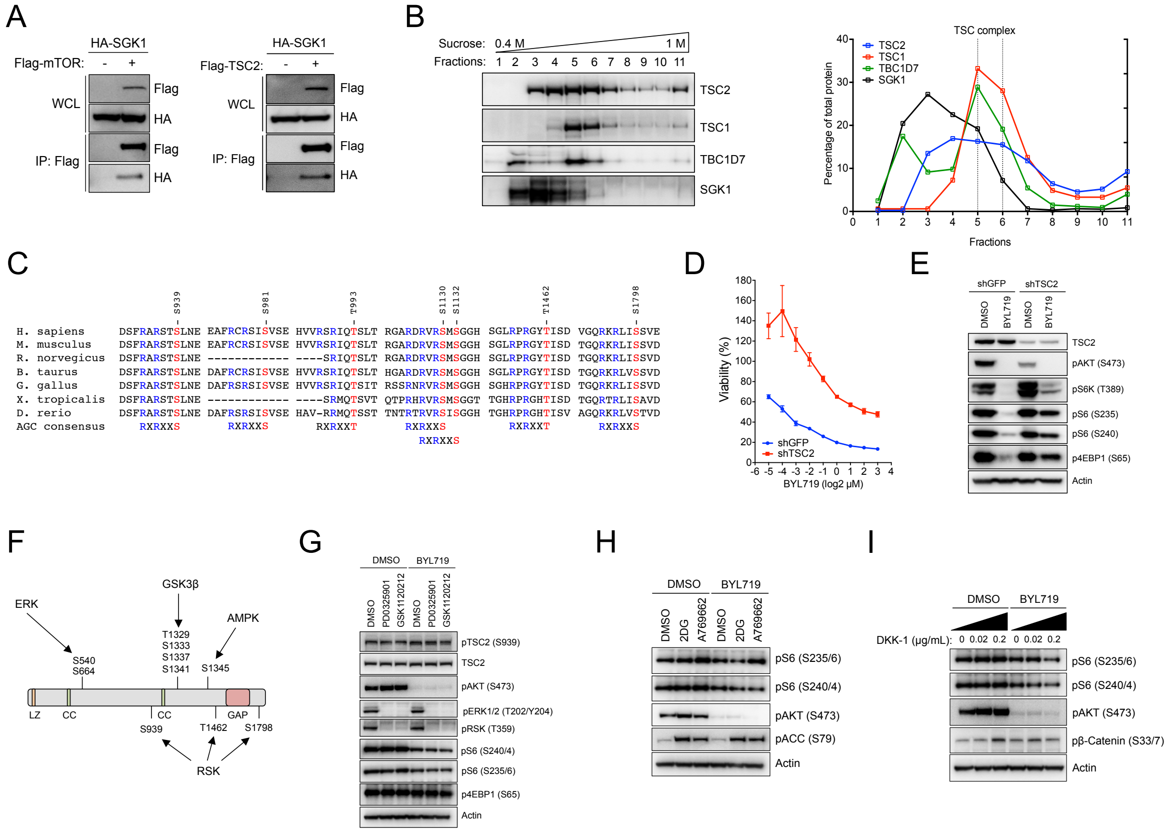

Figure S6. Relative to Figure 5

(A) Co-immunoprecipitation assay using Flag-mTOR (left) or Flag-TSC2 (right) and HA-SGK1 in 293T cells. **(B)** Western blot analysis of sucrose gradient fractions collected upon ultracentrifugation. Columns were packed in densities ranging from 0.4 to 1 M of sucrose, and a small aliquot of each fraction was analyzed by Western blot. Densitometry quantification of the sucrose gradient results are represented and dotted lines indicate the fractions in which the TSC complex is highly enriched, as assessed by the immunodetection of the three components TSC1, TSC2, and TBC1D7. **(C)** Alignment of the sequence of TSC2 comprising the AGC phosphorylation motifs RXRXX(S/T). R-1 and R-3 are highlighted in blue and phosphorylatable S or T in red. Alignment was performed with ClustalW2 using the protein sequence from mouse (*Mus musculus*), rat (*Rattus norvegicus*), cattle (*Bos taurus*), chicken (*Gallus gallus domesticus*), frog (*Xenopus tropicalis*), and zebrafish (*Danio rerio*). **(D)** Dose-response curves from T47D cells transduced with lentivirus expressing shGFP and shTSC2 and treated with increasing concentrations of BYL719 for 6 days. **(E)** Western blot from T47D shGFP and shTSC2 cells treated with BYL719 (1 μM) for 4 hr. **(F)** Representative signaling integration of other kinases involved in the phosphorylation of TSC2. Residues previously identified to be phosphorylated by the indicated kinases are shown. Domains are indicated: LZ (leucine zipper); CC (coiled coil); GAP (GTP-ase activation protein). **(G)** Western blot analysis of HCC1954 cells treated with the MEK inhibitors PD0325901 (1 μM) and GSK1120212 (50 nM) in the presence or absence of BYL719 (1 μM) for 4 hr. **(H)** Western blot analysis of HCC1954 cells treated with the AMPK inductors 2-deoxyglucose (50 mM) and A769662 (300 μM) in the presence or absence of BYL719 (1 μM) for 4 hr. Phosphorylation of the previously described substrate Acetyl-CoA Carboxylase (ACC) S79 is shown as control for AMPK activation. **(I)** Western blot analysis of HCC1954 cells treated with increasing concentrations of the WNT antagonist Dickkopf WNT signaling pathway inhibitor 1 (DKK-1) for 30 min in the presence or absence of BYL719 (1 μM) for 4 hr. Phosphorylation of the previously described substrate β-catenin S33/7 is shown as control for GSK3β activation.

Error bars are ± SEM.

Supplemental Experimental Procedures

RNAi screening

The synthetic lethal RNAi screening was carried out at the High-Throughput Screening Core Facility of MSKCC. The kinome and phosphatome Ambion Silencer Select v4.0 libraries were purchased from Life Technologies and contain 2130 unique siRNAs targeting each of the 710 human kinase genes and 894 unique siRNAs targeting each of the 298 human phosphatase genes. Diluted siRNA were transferred into assay plates at a final concentration of 50 nM. As a reference, we used Silencer Select Negative Control #1 siRNA (4390843) as a negative control and PLK1 siRNA (s449) as the positive control.
JIMT1 and HCC1954 cells were seeded and were reverse transfected using Dharmafect-1 at 0.05 µL/well. Next, cells were treated with DMSO or BYL719 1 µM and 7 days after transfection, cell viability was assessed using Alamar blue and Nuclei Count using Hoechst staining and quantified using LEADseeker (GE Healthcare) and INCA2000 (GE Healthcare), respectively.
For the hit nomination, the BDA method was used as previously described (Bhinder and Djaballah, 2012). Briefly, this method comprises 5 steps to analyze and score active siRNA duplexes and genes: (1) active duplex identification, (2) active gene identification, (3) off-target effects filtering, (4) re-scoring, and (5) biological classifications. To identify modulators of BYL719 resistance, active genes were nominated from the active siRNA duplexes using a hit rate per gene (H score) of ≥60. H score is defined as follows:

$$\text{H score} = \frac{\text{number of active siRNA duplex}}{\text{total number of siRNA duplexes}} \times 100$$

Using this approach, 5 genes were identified and the two most active duplexes of each gene were purchased and screened for cell viability and pS6 staining in the presence of BYL719 1 µM. siRNA were from Ambion: *PIK3CA* (s10520, s10522), *MTOR* (s602, s603), *PDPK1* (s10274, s10275), *PAPL* (s52890, s52892), and *PP1R12A* (s935, s937). Confirmation screening was carried out as described above. For pS6 (S240/4) staining, cells were reverse-transfected and after 72 hr, they were treated for 4 hr with BYL719 at 1 µM. Next, cells were fixed with 4% Paraformaldehyde in PBS and stained using pS6 (S240/4) antibody from Cell Signaling (2215), followed by Alexa Fluor 488 secondary antibody. Fluorescence was quantified using INCA2000 (GE Healthcare).
Final nomination was performed using the H score described above and genes that sensitized cells to BYL719 and decreased pS6 (S240/4) were selected.

Plasmids and site-directed mutagenesis

The Myc-tagged constructs pCCL-PDK1 WT, KD (K111N), K465E, and L155E were a gift from Dr. Primo and Dr. Gagliardi (University of Turin). pLPCX-HA-SGK1(Δ60) was obtained from Dr. Conzen (The University of Chicago) and was used as a template to subclone the cDNA and generate pdEYFP-SGK1(Δ60) and pLenti7.3-V5-SGK1(Δ60,S422D). The kinase-inactive K127A and constitutively active S422D mutant were generated using PCR-based site-directed mutagenesis.
Plasmids expressing Flag-tagged mTOR (26603), TSC1 (8995), TSC2 (8996), RHEB (15888), and HA-S6K (ΔCT, T389E) (8993) were obtained from Addgene. pcDNA3-Flag-TSC2 WT and 5A (S939A, S981A, S1130A, S1132A, T1462A) were a gift from Dr. Manning and were used as a template for the generation of pcDNA3-Flag-TSC2 6A (5A, S1798A) and pcDNA3-Flag-TSC2 6E, respectively. Plasmids encoding for the TSC2 truncation mutants were provided by Dr. Xiong (University of North Carolina at Chapel Hill) and pEGFP-TSC2 was from Dr. Krymskaya (University of Pennsylvania). *PDPK1* targeting shRNA pLKO-based vector used in this study was TRCN0000039782, although other clones were also tested. *RICTOR*-targeting shRNA plasmid was from Addgene (1853).
Lentiviral doxycycline-inducible mirE-embedded shRNAs were XhoI/EcoRI cloned into the LT3GEPIR vector previously described (Fellmann et al., 2013). Briefly, this all-in-one vector contains the puromycin resistance and the reverse transactivator (rtTA3) under the control of the constitutive phosphoglycerate kinase (PGK) promoter. The shRNA and the fluorescent marker GFP are expressed under the control of the Tet-responsive element promoter (T3G). Control shRNA was a hairpin designed against the *Renilla reniformis* luciferase. *SGK1* shRNA was chosen experimentally based on five different hairpins. The sequence targeting the exon 5 provided the most robust results.

REN shRNA:

5’-TGCTGTTGACAGTGAGCGCAGGAATTATAATGCTTATCTATAGTGAAGCCACAGATGTATAGATAA GCATTATAATTCCTATGCCTACTGCCTCGGA-3’

SGK1#282 shRNA:

5’-TGCTGTTGACAGTGAGCGCAGAAGTGTTCTATGCAGTCAATAGTGAAGCCACAGATGTATTGACTG CATAGAACACTTCTTTGCCTACTGCCTCGGA-3’

All constructs were validated by Sanger sequencing.

Cells and lentiviral production

All cell lines were obtained from ATCC except for JIMT1 (AddexBio), used at low passages, and maintained at 37°C in a 5% CO_2 atmosphere in the recommended culture media. HCT116 *PDPK1*$^{-/-}$ and *PDPK1*$^{+/+}$ cells were a gift from Dr. Mills (MD Anderson) and were originally generated by Dr. Vogelstein’s laboratory (Johns Hopkins University) (Ericson et al., 2010).
For lentiviral production, 293T cells were seeded in 10-cm plates, transfected with pCMV-VSVG, pCMV-dR8.2, and the plasmid of interest using FuGene HD (Promega). Viruses were collected 72 hr post-transfection, filtered through a 0.45 µm filter (Millipore), and recipient cells were infected twice using viral supernatants supplemented with 8 µg/µL of polybrene (Sigma). Transduced cells were selected using puromycin (2 µg/mL) or Fluorescence Activated Cell Sorting (FACS) for the pCCL and pLenti7.3 vectors, which contain EGFP as a selectable marker.

Reagents, cell viability and apoptosis

BYL719 and MK2206 were obtained from the Stand Up to Cancer (SU2C) pharmacy. GSK2334470 and Staurosporine were purchased at Selleckchem. SGK1-inh was a gift from M. Nazare and N. Halland. All drugs were dissolved in DMSO for in vitro experiments.
Cell viability was measures using the MTT assay. Briefly, 5000 cells were seeded in 96 well plates, treated for 6 days, and assayed using 0.25% MTT (Sigma) and 50 mM sodium succinate (Sigma) solutions for 3 hr. Formazan crystals were dissolved with DMSO and absorbance was measured at 570 nm of wavelength.
For Caspase 3/7 activity, the Caspase-Glo® 3/7 Assay kit from Promega was used following manufacturer’s instructions. The caspase inhibitor zVAD-fmk was used to inhibit apoptosis in cells and was also obtained from Promega.

Immunoblot, immunoprecipitation, and kinase assay

For western blot analysis, proteins were extracted in RIPA buffer supplemented with protease and phosphatase inhibitors (Roche). Protein lysates were separated using SDS-PAGE gels and transferred to a PVDF membrane. Then, membranes were probed using specific antibodies. PDK1, pAKT (S473), pAKT (T308), pS6K (T389), pS6 (S240/4), pS6 (235/6), p4EBP1 (S65), PARP, Actin, pRSK (S227), cleaved Caspase 3, pFOXO1/3 (T24/T32), SGK1, SGK2, SGK3, pNDRG1 (T346), NDRG1, Flag, HA, TSC2, pAcetyl-CoA Carboxylase (Ser79), pbetaCatenin (S33/7), pERK1/2 (T202/Y203), pRSK (T359), pTSC2 (S939), TBDC1D7, TSC1, and phospho-RXRXX(S/T) were from Cell Signaling Technology (CST). For S6K T229 phosphorylation detection we used pPKC (pan) (γT514) (9379) from CST, as previously reported (Garcia-Martinez and Alessi, 2008). The SGK1 and AKT antibodies for endogenous immunoprecipitation were raised in sheep by the Division of Signal Transduction Therapy (DSTT) at the University of Dundee and affinity-purified against the indicated antigens: anti-AKT1 (S695B, third bleed; raised against residues 466–480 of human Akt1: RPHFPQFSYSASGTA), anti-SGK1 antibody (S062D, third bleed, raised against recombinant SGK1 protein (DU35257). For endogenous co-immunoprecipitation of SGK1 and TSC2, we employed the S062D sheep antibody in 10 mg of JIMT1 lysate and TSC2 was recognized using the CST rabbit antibody with a secondary conformational specific antibody (Clean Blot from Thermo).
For immunoprecipitation assays, 293T cells were transiently transfected with appropriate plasmids and 24 hr post-transfection, cells were washed in cold PBS and lysed using NP-40 buffer (150 mM NaCl, 10 mM Tris pH 8, 1% NP-40, 10% glycerol). Lysates were rotated at 4°C for 4 hr with EZview™ Red ANTI-FLAG® M2 or ANTI-HA agarose beads (Sigma) and washed three times using NP-40 buffer. For in vitro kinase assay, immunoprecipitated

Flag-TSC2 was used as a substrate in a reaction with recombinant His-SGK1 (Δ60) (MRC-PPU Reagents) and ATP (Signalchem) in kinase assay buffer containing 25 mM MOPS pH 7.2, 12.5 mM β-glycerophosphate, 25 mM $MgCl_2$, 5 mM EGTA, 2 mM EDTA and 0.25 mM DTT at 30°C for 30 minutes. In vitro kinase activity of endogenous SGK1 and AKT was assayed by measuring [γ-^{32}P] ATP incorporation into Crosstide substrate peptide [GRPRTSSFAEGKK]. SGK1 and AKT were immunoprecipitated from HCC1954 cell line 4 hr after treatment. Immunoprecipitates were washed once with lysis buffer containing 500 mM NaCl, once with lysis buffer, and twice with Buffer A (50 mM Tris pH 7.5, 0.1 mM EGTA). Reactions were carried out in 40 µL total volume containing 0.1 mM [γ-^{32}P] ATP (400-1000 cpm/pmol), 10 mM magnesium acetate, and 30 µM Crosstide peptide. Reactions were terminated by adding 10 µL 0.1 mM EDTA. 40 µL of the reaction mix was spotted on P81 paper, which was immediately immersed into 50 mM ortophosphoric acid and washed several times. Papers were rinsed in acetone and air dried. Radioactivity was quantified by Cerenkov counting. One unit of enzyme activity was defined as the amount of enzyme that catalyzes incorporation of 1 nmol of [γ-^{32}P] ATP into the substrate over one minute.

m^{7}GTP pull downs

2 million cells were seeded in 10 cm plates and treated accordingly 12 hr after seeding. Lysates were prepared using m^{7}GTP pull down buffer (50 mM Hepes, pH 7.4, 75 mM NaCl, 10 mM MgCl2, 1 mM DTT, 8 mM EGTA, 10 mM β-glycerophosphate, 0.5 mM Na_3VO_4, 0.5% Triton X-100) supplemented with protease and phosphatase inhibitors. Lysates were centrifuged at 13000 rpm for 10 min and supernatants were rotated for 2 h at 4°C with 7-methyl-GTP-Sepharose or control Sepharose beads (Jena Bioscience). Beads were washed three times with m^{7}GTP pull down buffer, resuspended in Laemmli buffer, and associated proteins were detected by Western blot.

Mass spectrometry

Kinase assay reactions were performed in biological triplicates and resolved using SDS-polyacrylamide gel electrophoresis, stained with SimplyBlue SafeStain (Life Technologies, Thermo Fisher Scientific), and the band corresponding to Flag-TSC2 was excised and digested with trypsin as described by (Shevchenko et al., 2006). The tryptic peptides were resuspended in buffer A containing 3% formic acid and analyzed by microcapillary liquid chromatography with tandem mass spectrometry using a NanoAcquity LC (Waters) with a 100 µm-inner-diameter x 10 cm-length C18 column (1.7µm BEH130, Waters) configured with a 180 µm x 2 cm trap column coupled to a Q-Exactive mass spectrometer (Thermo Fisher Scientific) scanning 380-1800 m/z at 70,000 resolution with AGC set at 3 x 10^6. Peptides were eluted with a linear gradient of 2-30% acetonitrile (0.1% formic acid) in water over 90 min at a flow rate of 300 nL/min. Key parameters for the data dependent MS were top 10 DDA, AGC 5e4, and ms/ms resolution of 17,000. Data were analyzed using MaxQuant (Max Planck Institute of Biochemistry, Germany; version 1.5.1.0) at default settings with a few modifications. The default was used for first search tolerance and main search tolerance: 20 ppm and 6 ppm, respectively. MaxQuant was set up to search the reference human proteome database. Maxquant performed the search using trypsin digestion with up to 2 missed cleavages. Peptide, Site and Protein FDR were all set to 1% with a minimum of 1 peptide needed for identification but 2 peptides needed to calculate a protein ratio. LFQ quantitation was confirmed by manual integration of the MS1 data for the phosphorylation sites of interest. Raw data as well as original MaxQuant results files can be provided upon request.

Microarray, qPCR, and ChIP-qPCR

RNA was isolated from cells using the QIAGEN RNeasy kit. For microarray analysis, biotinylated cRNA was prepared according to the standard Illumina protocol. After fragmentation, cRNA was hybridized with Illumina GX HT12 Human Array. Slides were washed and stained in the Illumina instrument following manufacturer's protocol. Slides were scanned using Illumina Bead Array Reader. Data were analyzed using GenomeStudio software. No normalization and background correction are performed first, then quantile normalization and background correction are done.
For mRNA expression analysis, cDNA was prepared using the Bio-Rad cDNA synthesis kit. cDNA was amplified by quantitative PCR using SYBR Select Master Mix (Applied Biosystems) in the ViiA 7 Real-Time PCR system. All reactions were carried out in triplicate. Primers used for mRNA expression were:

ERBB3: Fw-CTGATCACCGGCCTCAAT; Rv-GGAAGACATTGAGCTTCTCTGG
IRS2: Fw-TTCTTGTCCCACCACTTGAA; Rv-CTGACATGTGACATCCTGGTG
TNFSF10: Fw-CCTCAGAGAGTAGCAGCTCACA; Rv-CAGAGCCTTTTCATTCTTGGA

BCL6: Fw-CTGCAGATGGAGCATGTTGT; Rv-TCTTCACGAGGAGGCTTGAT
Actin: Fw-CGTCTTCCCCTCCATCGT; Rv-GAAGGTGTGGTGCCAGATTT

ChIP assays were performed as described previously (Toska et al., 2012). Briefly, cells were treated with 1% formaldehyde for 15 min at room temperature and quenched with ice-cold 125 nM glycine for 5 min. Lysed cells were sonicated on ice to yield 200-800 bp DNA fragments. Chromatin was incubated overnight at 4°C with 2 µg of anti-FOXO3A antibody (Santa Cruz Biotechnology; sc-11351), anti-RNA polymerase II CTD (YSPTSPS) antibody (Abcam; ab817), anti-RNA polymerase II CTD (phospho S5) (Abcam; ab5131) or nonspecific IgG. Immunocomplexes were precipitated by incubation overnight with protein G-conjugated beads. Immunoprecipitates were washed and crosslinks were reversed by heating to 65°C for 6 hr and then treated with proteinase K for 1 hr at 55°C. Chromatin was purified using QiaQuick PCR clean-up columns. ChIP primers used in this study were:
Control: Fw-CAGCTCAGTGCTGTTGGTGG; Rv-ACCATCCAACCCTGGAGATC
IRS2 promoter: Fw-GACAATCAAAGTCCTTCCCAAA; Rv-CCTTTTGACCTGTGCTGTTGT
TNFSF10 promoter, Fw-AAAGAAAATCCCTCCCCTCTT; Rv-CACTCACCTCAAGCCCATTT
SGK1 promoter, Fw- GGGAGGGAGAGGTCAGGAAT; Rv-TCGCTTGTTACCTCCTCACG

Animal studies and IHC

Animals were maintained and treated in accordance with Institutional Guidelines of Memorial Sloan Kettering Cancer Center (Protocol number 12-10-019). 5 x 10^6 cells in 1:1 PBS/Matrigel (Corning) were injected subcutaneously into six-week-old female athymic *Foxn1*^{nu} nude mice. When tumors reached a volume of ~150mm^3, mice were randomized, treated, and tumors were measured twice a week during a month. At least 10 tumors per group were used in all the studies. Treatments were as follows: BYL719 (25 mg × kg^{-1} in 0.5% carboxymethylcellulose (Sigma), daily p.o.); GSK2334470 (100 mg × kg^{-1} in 10% of 1:1 Kolliphor® EL/EtOH (Sigma), three times/week, i.p.); SGK1-inh (50 mg × kg^{-1} in 40% of 3:1 Glycofurol/Kolliphor® RH 40 mixture (Sigma) in 0.9% saline, daily p.o.). Tumors were harvested at the end of the experiment 3 hr after the last dosage, fixed in 4% formaldehyde in PBS, and paraffin-embedded. IHC was performed on a Ventana Discovery XT processor platform using standard protocols and the following antibodies from Cell Signaling Technology: pAKT(S473) (4060),1:70; pS6 (S240/4) (5364),1:500; pNDRG1 (T346) (5482), 1:200; p4EBP1 (T37/46) (2855), 1:300. Primary staining was followed by 60 minutes incubation with biotinylated goat anti-rabbit IgG (Vector labs) 1:200. Blocker D, Streptavidin- HRP and DAB detection kit (Ventana Medical Systems) were used according to the manufacturer instructions.

Docking and molecular dynamics simulations

The structure of SGK1 kinase is only available in its inactive form, with missing structural information such as the coordinates of the αC helix. We constructed the 3D structures of SGK1 kinase both in its active and inactive forms using comparative modeling methods based on homology. The templates used were the available crystal structure of SGK1 kinase in the inactive state (PDB: 2R5T) (Zhao et al., 2007), high-resolution crystal structure of the kinase domain of AKT (55% homology) in its active (PDB: 1O6K) (Yang et al., 2002a) and inactive (PDB: 1GZN) (Yang et al., 2002b) states. The program Modeller (version 9.12) (Sali and Blundell, 1993) was used for the generation of homology models. Several models were generated and the models with the best physicochemical properties were further refined using all atom molecular dynamics (MD) simulations.
The 3D structures of SGK1-inh and ATP were built using the Maestro module and minimized using the Macromodel module, employing the OPLS-2005 force field, in the program Schrodinger 9.0. The minimized SGK1 inhibitor and ATP were docked into the binding pockets of SGK1 kinase models with Glide (Friesner et al., 2004) using standard docking protocols (Kannan et al., 2015). Refinement of the docked models of SGK1-inhibitor and SGK1-ATP complexes were carried out using MD simulations under the Sander module of the program Amber14. The partial charges and force field parameters for SGK1 inhibitor and ATP were generated using the Antechamber module in Amber. All atom versions of the Amber 03 force field (ff03) (Duan et al., 2003) and the general Amber force field (GAFF) (Wang et al., 2004) were used for the protein and the inhibitors respectively. All the simulations were carried out at 300 K using standard protocols (Kannan et al., 2015). Three independent MD simulations (assigning different initial velocities) were carried out on each equilibrated SGK1-ATP and SGK1-inhibitor structure for 100 ns each, with conformations saved every 10 ps. Simulation trajectories were visualized using VMD (Humphrey et al., 1996) and figures were generated using Pymol.
The binding free energies (enthalpic components), energy decompositions (to identify "hot spot" residues) and

computational alanine scans (of the "hot spot" residues) were calculated using the MMPBSA (Molecular Mechanics Poisson–Boltzmann Surface Area) methodology (Kannan et al., 2015).

DNA methylation quantification

For DNA methylation analyses, bisulfite conversion of 500 ng of genomic DNA was performed using the EZ DNA Methylation Gold kit (Zymo Research, Orange, CA, USA) following the manufacturer's indications. For bisulfite sequencing, specific primers were designed to amplify the annotated promoter region using the MethylExpress program (Applied Biosystems) (Fw-AATTTTAGAATTTGGAAGAGGA and Rv-ACAACCTTAAATTAAACCCAAA), and a minimum of eight single clones was interrogated for each cell line. In order to quantify the absolute levels of DNA methylation on CpG sites in the proximity of the transcription start site of SGK1 we carried out pyrosequencing on bisulfite-treated DNA using specific primers designed with the PyroMark Assay Design Software (Qiagen, version 2.0.01.15) (Fw- GAGGGAGAGGTTAGGAATGT, Rv-CCCTCCCTTCRCTTATTACCTCCTCAC and Seq- TTTTGAAGTAATTTTTGAGAATATT). Pyrosequencing reactions and quantification of DNA methylation values were performed in a PyroMark Q96 System version 2.0.6 (Qiagen) including appropriate controls. As previously described, *SGK1* DNA methylation levels were categorized into three groups. DNA methylation values in the first group (<33%) were defined as low DNA methylation, and high DNA methylation was assigned to values on the two top groups (>33%).

FRET

For FRET experiments, HeLa cells were seeded in chambered coverglass and transfected with 0.5 µg of EGFP Donor plasmid, 0.5 µg of EYFP Acceptor plasmid, or both constructs. 16 hr post-transfection cells were imaged with a Leica TCS SP8 microscope using the established parameters for Donor (Ex: 458 nm laser at 15%; Em: 466-501 nm) and Acceptor (Ex: 528 nm laser at 3%; Em: 555-600 nm).
FRET efficiency was calculated using the following equation as described in (van Rheenen et al., 2004):

$$E_{\mathrm{FRET}} = \frac{FRET - EGFP \times \beta - EYFP \times (\gamma - \alpha \times \beta)}{EYFP \times (1 - \beta \times \delta)}$$

Where FRET, EGFP and EYFP refers to the FRET, Donor and Acceptor channels respectively. The corrections factors were α=0.01; β=0.37; γ=0.31; δ=0.02, where α corrects for acceptor cross-excitation crosstalk (α=Donor/Acceptor), β corrects for donor crosstalk (β=FRET/Donor), γ corrects for acceptor cross-excitation (γ=FRET/Acceptor), and δ corrects for FRET crosstalk (δ=Donor/FRET). Mock-transfected cells were used to calculate the background threshold level (background intensity mean + 4 Standard deviation).

TMA and patients

Formalin-fixed paraffin-embedded (FFPE) tissue blocks from primary invasive breast carcinomas were used to construct the TMA reported in this study. A certified pathologist (E.B.) microscopically examined hematoxylin and eosin-stained sections of all the tumors and selected representative areas, excluding foci of ductal carcinoma in situ and tumor necrosis. All carcinomas were represented in the TMAs in triplicate 0.6-mm cores. An Automatic Tissue MicroArrayer (ATA-27, Beecher Instruments Inc) was used to construct TMAs from a total of 273 breast invasive carcinomas. This comprised clinically and pathologically confirmed triple-negative breast cancer patients (138), ER/PR receptor-positive breast cancer patients (68), and HER2-positive cancer patients (67). Tumor were considered ER/PR receptor-positive if >10% of neoplastic cells showed nuclear positivity. Cases with HER-2 staining intensity of 3+ were considered positive, whereas those with 2+ staining intensity of HER2 were further evaluated by ERBB2 FISH using the PathVision HER2 probe Kit (Abbott Laboratories), and scored as positive if the HER2/Cep17 ratio was 2.2 or greater. 5-µm thick TMA sections were stained for pNDRG1 (T346) following the protocol described above. Based on the observed staining across the different samples, cases were scored as High expression when pNDRG1 staining intensity of 2+ was found >20% of the neoplastic cells. Intermediate staining represented tumors that had 1+ staining intensity in >10% of the neoplastic cells.
For the study of patients treated with the PI3Kα inhibitor BYL719, pre-treatment biopsy FFPE blocks from patients enrolled in the clinical trial NCT01870505 conducted at MSKCC were used for IHC as described above. For the selection of the patients, *PIK3CA* and other tumor genomic drivers were analyzed using MSK-IMPACT (Cheng et al., 2015). Only patients that did not exhibit toxicity during the trial, harbored hot-spot mutations in *PIK3CA*, and

did not harbor mutations in *PTEN* or *KRAS* (known to cause resistance to PI3Kα inhibitors) were selected for the biomarker study. *SGK1* mRNA levels were determined using next-generation sequencing (NGS) and the expression results were presented as raw Reads Per Kilobase of transcript per Million mapped reads (RPKM). Mean and standard deviation (SD) was calculated across all the samples with available NGS data and overexpression of *SGK1* was called for the samples with mRNA levels greater than mean+1SD. For 6 samples, RNA quality and quantity was not optimal for NGS. In these cases, *SGK1* mRNA levels were determined using RT-qPCR as described above. RNA from low expressing (T47D) and high expressing (HCC1954) cell lines were used as positive control and absolute mRNA levels were quantified. Primers used for the detection of *SGK1* were:
Fw: GACAGGACTGTGGACTGGTG; Rv: TTTCAGCTGTGTTTCGGCTA
The MSKCC Institutional Review Board approved the study and informed consent was obtained from all subjects.

Supplemental References

Bhinder, B., and Djaballah, H. (2012). A simple method for analyzing actives in random RNAi screens: introducing the "H Score" for hit nomination & gene prioritization. Comb Chem High Throughput Screen *15*, 686-704.

Cheng, D. T., Mitchell, T. N., Zehir, A., Shah, R. H., Benayed, R., Syed, A., Chandramohan, R., Liu, Z. Y., Won, H. H., Scott, S. N.*, et al.* (2015). Memorial Sloan Kettering-Integrated Mutation Profiling of Actionable Cancer Targets (MSK-IMPACT): A Hybridization Capture-Based Next-Generation Sequencing Clinical Assay for Solid Tumor Molecular Oncology. J Mol Diagn *17*, 251-264.

Duan, Y., Wu, C., Chowdhury, S., Lee, M. C., Xiong, G., Zhang, W., Yang, R., Cieplak, P., Luo, R., Lee, T.*, et al.* (2003). A point-charge force field for molecular mechanics simulations of proteins based on condensed-phase quantum mechanical calculations. J Comput Chem *24*, 1999-2012.

Fellmann, C., Hoffmann, T., Sridhar, V., Hopfgartner, B., Muhar, M., Roth, M., Lai, D.Y., Barbosa, I.A.M., Kwon, J.S., Guan, Y., Sinha, N., Zuber, J. (2013). An Optimized microRNA Backbone for Effective Single-Copy RNAi. Cell Reports *5 (6)*,1704–1713.

Friesner, R. A., Banks, J. L., Murphy, R. B., Halgren, T. A., Klicic, J. J., Mainz, D. T., Repasky, M. P., Knoll, E. H., Shelley, M., Perry, J. K.*, et al.* (2004). Glide: a new approach for rapid, accurate docking and scoring. 1. Method and assessment of docking accuracy. J Med Chem *47*, 1739-1749.
Garcia-Martinez, J. M., and Alessi, D. R. (2008). mTOR complex 2 (mTORC2) controls hydrophobic motif phosphorylation and activation of serum- and glucocorticoid-induced protein kinase 1 (SGK1). Biochem J *416*, 375-385.

Humphrey, W., Dalke, A., and Schulten, K. (1996). VMD: visual molecular dynamics. J Mol Graph *14*, 33-38, 27-38.

Kannan, S., Poulsen, A., Yang, H. Y., Ho, M., Ang, S. H., Eldwin, T. S., Jeyaraj, D. A., Chennamaneni, L. R., Liu, B., Hill, J.*, et al.* (2015). Probing the binding mechanism of Mnk inhibitors by docking and molecular dynamics simulations. Biochemistry *54*, 32-46.

Sali, A., and Blundell, T. L. (1993). Comparative protein modelling by satisfaction of spatial restraints. J Mol Biol *234*, 779-815.

Shevchenko, A., Tomas, H., Havlis, J., Olsen, J. V., and Mann, M. (2006). In-gel digestion for mass spectrometric characterization of proteins and proteomes. Nat Protoc *1*, 2856-2860.

Toska, E., Campbell, H. A., Shandilya, J., Goodfellow, S. J., Shore, P., Medler, K. F., and Roberts, S. G. (2012). Repression of transcription by WT1-BASP1 requires the myristoylation of BASP1 and the PIP2-dependent recruitment of histone deacetylase. Cell Rep *2*, 462-469.

van Rheenen, J., Langeslag, M., and Jalink, K. (2004). Correcting confocal acquisition to optimize imaging of fluorescence resonance energy transfer by sensitized emission. Biophys J *86*, 2517-2529.

Wang, J., Wolf, R. M., Caldwell, J. W., Kollman, P. A., and Case, D. A. (2004). Development and testing of a general amber force field. J Comput Chem *25*, 1157-1174.

Yang, J., Cron, P., Good, V. M., Thompson, V., Hemmings, B. A., and Barford, D. (2002a). Crystal structure of an activated Akt/protein kinase B ternary complex with GSK3-peptide and AMP-PNP. Nat Struct Biol *9*, 940-944.

Yang, J., Cron, P., Thompson, V., Good, V. M., Hess, D., Hemmings, B. A., and Barford, D. (2002b). Molecular mechanism for the regulation of protein kinase B/Akt by hydrophobic motif phosphorylation. Mol Cell *9*, 1227-1240.

Zhao, B., Lehr, R., Smallwood, A. M., Ho, T. F., Maley, K., Randall, T., Head, M. S., Koretke, K. K., and Schnackenberg, C. G. (2007). Crystal structure of the kinase domain of serum and glucocorticoid-regulated kinase 1 in complex with AMP PNP. Protein Sci *16*, 2761-2769.
