# Supplementary material for: PDK1-SGK1 Signaling Sustains AKT-Independent mTORC1 Activation and Confers Resistance to PI3Kα Inhibition
Source: Cancer Cell. 2016 Aug 8;30(2):229–42. doi: 10.1016/j.ccell.2016.06.004 (PMC4982440; doi:10.1016/j.ccell.2016.06.004)
Supplement: Document S2. Article plus Supplemental Information [file mmc2.pdf]

Cancer Cell

Article

PDK1-SGK1 Signaling Sustains AKT-Independent mTORC1 Activation and Confers Resistance to PI3Kα Inhibition

Graphical Abstract

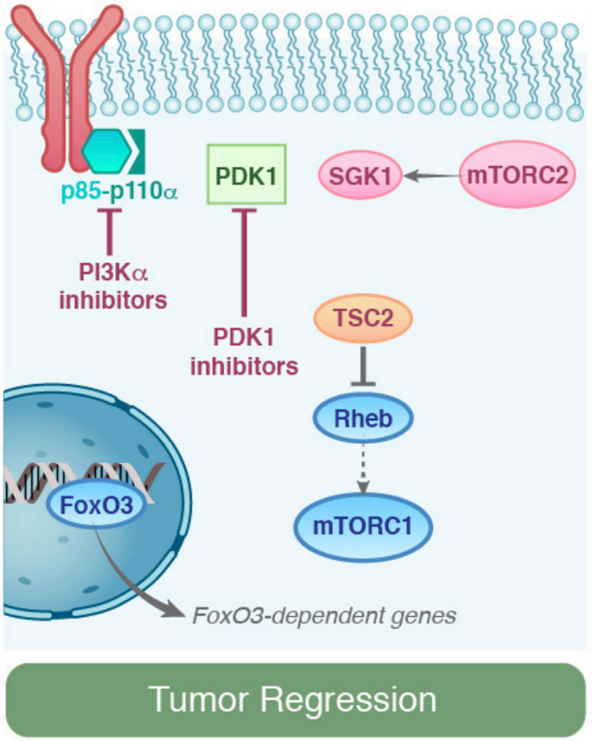

Authors

Pau Castel, Haley Ellis, Ruzica Bago, ..., Dario R. Alessi, José Baselga, Maurizio Scaltriti

Correspondence

baselgaj@mskcc.org (J.B.), scaltrim@mskcc.org (M.S.)

In Brief

Castel et al. show that PDK1 activates SGK1 to phosphorylate and inhibit TSC2, contributing to maintenance of mTORC1 activity and resistance of PIK3CA-mutant cancer cells to PI3Kα inhibition. Targeting either PDK1 or SGK1 restores the sensitivity of these resistant cancer cells to PI3Kα inhibition.

Highlights

- The PDK1-SGK1 axis can sustain mTORC1 activity upon PI3K/AKT suppression
- SGK1 directly phosphorylates TSC2, resulting in mTORC1 activation
- Targeting PDK1 sensitizes *PIK3CA*-mutant cells to PI3Kα inhibition

Accession Numbers

GSE69189

Castel et al., 2016, Cancer Cell *30*, 229–242
August 8, 2016 © 2016 The Author(s). Published by Elsevier Inc.
http://dx.doi.org/10.1016/j.ccell.2016.06.004

Cancer Cell
Article

PDK1-SGK1 Signaling Sustains AKT-Independent mTORC1 Activation and Confers Resistance to PI3Kα Inhibition

Pau Castel,[1] Haley Ellis,[1] Ruzica Bago,[2] Eneda Toska,[1] Pedram Razavi,[1,3] F. Javier Carmona,[1] Srinivasaraghavan Kannan,[4] Chandra S. Verma,[4,5,6] Maura Dickler,[3] Sarat Chandarlapaty,[1,3] Edi Brogi,[7] Dario R. Alessi,[2] José Baselga,[1,3,*] and Maurizio Scaltriti[1,7,*]
[1]Human Oncology & Pathogenesis Program (HOPP), Memorial Sloan Kettering Cancer Center, 1275 York Avenue, Box 20, New York, NY 10065, USA
[2]MRC Protein Phosphorylation and Ubiquitylation Unit, College of Life Sciences, University of Dundee, Dow Street, Dundee DD1 5EH, Scotland
[3]Department of Medicine, Memorial Sloan Kettering Cancer Center, 1275 York Avenue, Box 20, Suite M2015, New York, NY 10065, USA
[4]Bioinformatics Institute (A*STAR), 30 Biopolis Street, #07-01 Matrix, Singapore 138671, Singapore
[5]School of Biological Sciences, Nanyang Technological University, 60 Nanyang Drive, Singapore 637551, Singapore
[6]Department of Biological Sciences, National University of Singapore, 14 Science Drive 4, Singapore 117543, Singapore
[7]Department of Pathology, Memorial Sloan Kettering Cancer Center, 1275 York Avenue, Box 20, New York, NY 10065, USA
*Correspondence: baselgaj@mskcc.org (J.B.), scaltrim@mskcc.org (M.S.)
http://dx.doi.org/10.1016/j.ccell.2016.06.004

SUMMARY

***PIK3CA*, which encodes the p110α subunit of PI3K, is frequently mutated and oncogenic in breast cancer. PI3Kα inhibitors are in clinical development and despite promising early clinical activity, intrinsic resistance is frequent among patients. We have previously reported that residual downstream mTORC1 activity upon treatment with PI3Kα inhibitors drives resistance to these agents. However, the mechanism underlying this phenotype is not fully understood. Here we show that in cancer cells resistant to PI3Kα inhibition, PDK1 blockade restores sensitivity to these therapies. SGK1, which is activated by PDK1, contributes to the maintenance of residual mTORC1 activity through direct phosphorylation and inhibition of TSC2. Targeting either PDK1 or SGK1 prevents mTORC1 activation, restoring the antitumoral effects of PI3Kα inhibition in resistant cells.**

INTRODUCTION

The phosphoinositide 3-kinase (PI3K) pathway integrates many extracellular stimuli and triggers the phosphorylation of key downstream effectors such as AKT and the mammalian Target of Rapamycin Complex 1 and 2 (mTORC1 and 2). This signaling cascade is essential for regulating cell size, proliferation, survival, and metabolism (Engelman, 2009; Thorpe et al., 2015). Activation of PI3K results in increased phosphatidylinositol-(3,4,5)-triphosphate (PIP_3) at the plasma membrane, promoting the recruitment of the pleckstrin homology (PH) domain-containing proteins PDK1 and AKT (Currie et al., 1999; Pearce et al., 2010), where the constitutively active kinase PDK1 phosphorylates AKT at the activation loop (T308) (Alessi et al., 1997) and mTORC2 in the hydrophobic motif (S473) (Sarbassov et al., 2005). Once active, AKT phosphorylates a variety of antiapoptotic and cell-cycle-related proteins as well as transcription factors (Manning and Cantley, 2007). Moreover, AKT activates downstream mTORC1 through the phosphorylation of the negative regulators TSC2 and PRAS40 (Inoki et al., 2002; Manning et al., 2002; Potter et al., 2002; Sancak et al., 2007).

Significance

Promising clinical activity has been reported in breast tumors bearing activating mutations in *PIK3CA*, the gene that encodes for the α isoform of the p110 catalytic subunit of PI3K. However, despite these encouraging results some tumors are resistant to these agents. In this work we show that, upon PI3Kα inhibition, the PDK1-SGK1 axis can overcome AKT inhibition by activating mTORC1 via direct phosphorylation of TSC2. Therefore, both AKT and PDK1 must be suppressed to achieve full mTORC1 inhibition and antitumor activity in these tumors resistant to PI3Kα inhibition. This study uncovers a mechanism of cell survival under pharmacological pressure that can be exploited to improve the therapeutic options of patients with breast cancer.

This is an open access article under the CC BY license (http://creativecommons.org/licenses/by/4.0/).

Activating mutations in *PIK3CA*, which encodes the α isoform of the p110 catalytic subunit of PI3K, results in hyperactivation of the PI3K/AKT/mTOR pathway (Engelman, 2009). These mutations are common in breast cancer and provide the rationale for the development of inhibitors targeting the different nodes of the PI3K pathway (Fruman and Rommel, 2014).

PI3Kα-specific inhibitors are showing promising results in patients with tumors bearing activating *PIK3CA* mutations, but not all such tumors are sensitive (Juric et al., 2012, 2013). Understanding the molecular mechanisms by which tumors bypass the pharmacological inactivation of PI3Kα is crucial for the identification of patients more likely to respond to these inhibitors and for devising therapeutic strategies to improve the clinical benefit.

We previously reported that the activation status of mTORC1 upon PI3Kα blockade is a determinant of drug sensitivity in *PIK3CA*-mutant tumors. Despite full inhibition of PI3K/AKT, the presence of residual mTORC1 activity is sufficient to weaken the antitumor activity of PI3Kα inhibition. Resistant tumors are sensitized by co-treatment with the mTORC1 allosteric inhibitor everolimus, underscoring the causative role of mTORC1 in limiting the effects of PI3Kα blockade (Elkabets et al., 2013). In this work, we elucidate the molecular pathway that allows mTORC1 to retain activity in the presence of PI3K and AKT inactivation.

RESULTS

PDK1 Inhibition Sensitizes Resistant Cells to BYL719

Aiming to identify possible kinases or phosphatases responsible for the AKT-independent sustained mTORC1 activity in cells resistant to PI3Kα inhibition, we performed a small interfering RNA (siRNA) screen using a library targeting 710 kinases and 298 phosphatases encoded in the human genome. We measured cell viability and S6 ribosomal protein (S6) phosphorylation, a bona fide readout of mTORC1 activity, in the presence of BY719, a PI3Kα-specific inhibitor.

The screen design is shown in Figure S1A. Three different siRNAs targeting each member of the kinome/phosphatome were transfected in JIMT1 and HCC1954 cell lines, both of which are *PIK3CA* mutant and insensitive to BYL719. After treatment over 6 days, we found that knockdown of 37 enzymes in HCC1954 and 35 enzymes in JIMT1 sensitized cells to PI3Kα inhibition (Figure S1B and Table S1). Among these, five were found to be shared in both cell lines: *MTOR*, *PDPK1*, *PIK3CA*, *PPP1R12A*, and *PAPL* (Figure S1B). These findings were validated with a second targeted screening using the two most active siRNAs against each of these five targets, interrogating for both cell viability and phosphorylation of S6. With this more stringent approach, we found that only knockdowns of mTOR and PDK1 were capable of reducing S6 phosphorylation (S240/4) in the presence of PI3Kα inhibition (Figure S1C). While the finding of mTOR confirmed our previous data (Elkabets et al., 2013), the contribution of PDK1 in maintaining the resistant phenotype was an original finding.

PDK1 is a kinase that belongs to the Containing PKA, PKG, and PKC (AGC) kinase family that includes AKT, PKC, RSK, SGK, and S6K (Pearce et al., 2010). To confirm that PDK1 limits the sensitivity to PI3Kα inhibition by maintaining mTORC1 activity upon PI3Kα inhibition, we generated HCC1954 and JIMT1 cell lines stably expressing a PDK1 short hairpin RNA (shRNA). We observed that PDK1 knockdown is sufficient to decrease cell viability upon BYL719 treatment (Figures 1A and S2A). As previously described, treatment with BYL719 alone reduced AKT phosphorylation (S473 and T308) but not downstream mTORC1 targets (Elkabets et al., 2013). In contrast, the combination of PDK1 knockdown with BYL719 decreased the phosphorylation of the mTORC1 downstream targets p70 S6 kinase (S6K) and translation initiation factor 4E-binding protein (4EBP1), as well as phosphorylated S6 at both S240/4 and S235/6 sites (Figures 1B and S2B). As a result, the combination of BYL719 and PDK1 knockdown decreased cap-dependent translation (Figure S2C), a cellular process directly regulated by mTORC1 (Silvera et al., 2010). In PDK1 knockdown cells, inhibition of PI3Kα induced an increased binding of 4EBP1 to the cap m^7GpppN mRNA analog m^7GTP, to a similar extent as the mTOR kinase inhibitor AZD8055. On the contrary, we observed a reduction of the eukaryotic initiation factors (eIF) eIF4G and eIF4A, components of the eIF4F cap-initiation complex. As expected, eIF4E remained unchanged. In long-term treatments, the combination of BYL719 and PDK1 knockdown induced poly(ADP-ribose)polymerase (PARP) cleavage (Figure 1C) and increased caspase 3/7 activity (Figure 1D), surrogate markers of apoptotic activity.

Pharmacological inhibition of PI3Kα resulted in a modest delay in tumor growth in shGFP control xenografts but was sufficient to induce durable tumor shrinkage in tumors with ablated PDK1 (Figure 1E). Analysis of the tumors showed that BYL719 treatment effectively suppressed AKT phosphorylation (S473) in both shGFP and shPDK1 tumors, whereas S6 and 4EBP1 phosphorylation was inhibited only in shPDK1 xenografts (Figures 1F and S2D).

Next, we tested the activity of BYL719 in combination with GSK2334470, a highly selective PDK1 inhibitor (Najafov et al., 2011). We determined the appropriate dose of GSK2334470 to be used in combination with PI3Kα inhibition by analyzing both phosphorylation of the PDK1 target RSK2 (S227) and cell viability upon incubation with increasing concentrations of the PDK1 inhibitor. At 1 μM, pRSK2 (S227) was appreciably reduced (Figure S2E) with no significant changes in cell viability (Figure S2F). Despite the minimal effect on cell viability when used as a single agent, treatment with GSK2334470 was sufficient to sensitize both HCC1954 and JIMT1 cells and the triple-negative BYL719-resistant breast cancer cell line BT20 to PI3Kα inhibition (Figures 1G and S2G–S2H). Again, we observed that only the combination of BYL719 and GSK2334470 resulted in the inhibition of AKT and mTORC1 (Figures 1H, S2I, and S2J). Some residual pS6 was observed in BT20 cells, which might be attributed to the heterogeneity of the cell line or additional mechanisms that regulate S6 phosphorylation.

PDK1 inhibition did not decrease the phosphorylation of AKT at the activation loop (T308) as a result of a compensatory mechanism involving PIP3 and mTORC2, an observation in line with previous reports (Najafov et al., 2012). Analysis of cap-dependent translation complex formation revealed an increase in 4EBP1 and a decrease in eIF4G and eIF4A in m^7GTP pulldowns when both drugs were combined, consistent with mTORC1 inhibition (Figure S2K). Consistent with the knockdown experiments, the combination of BYL719 and GSK2334470 induced apoptosis

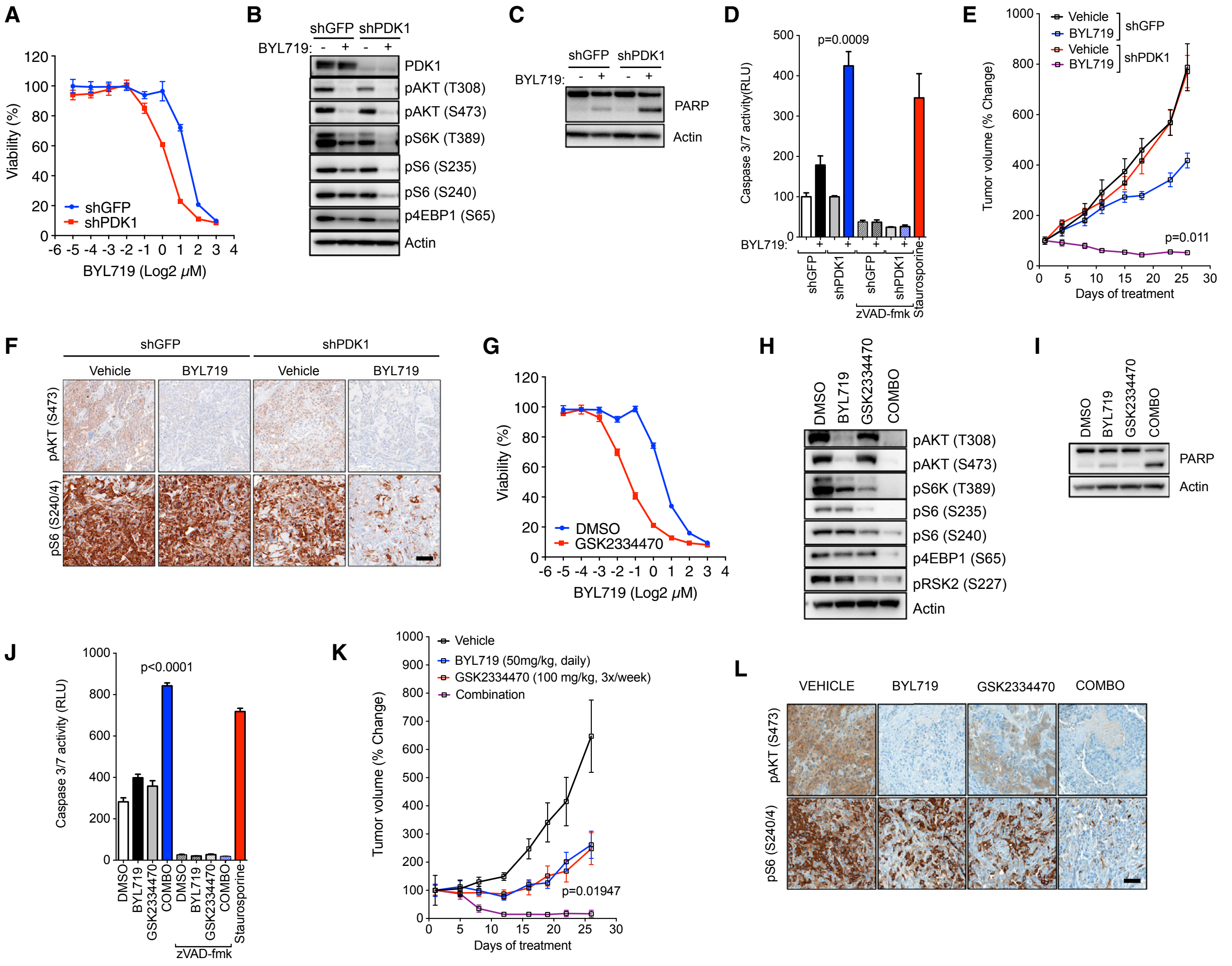

Figure 1. PDK1 Inhibition Sensitizes Resistant Cells to BYL719

(A) Dose-response curves from HCC1954 cells transduced with shGFP and shPDK1 and treated with BYL719 for 6 days.
(B) Western blot comparing cells from (A) treated with BYL719 (1 μM) for 4 hr.
(C) PARP western blot in cells transduced with shGFP and shPDK1 and treated with BYL719 (1 μM) for 24 hr.
(D) Caspase 3/7 DEVDase activity of HCC1954 shGFP and shPDK1 cells treated with BYL719 (1 μM) for 12 hr in the presence or absence of caspase inhibitor zVAD-fmk (20 μM). Staurosporine was used as a positive control (1 μM; 4 hr).
(E) HCC1954 shGFP and shPDK1 xenografts treated with vehicle or BYL719 (n = 10/arm).
(F) IHC analysis of tumors from (E) collected at the end of the experiment after 4 hr of the last treatment. Scale bar, 100 μm.
(G) Dose-response curves from HCC1954 cells treated with BYL719 in the presence or absence of GSK2334470 (1 μM) over 6 days.
(H) Western blot comparing HCC1954 cells treated with BYL719 (1 μM), GSK2334470 (1 μM), or the combination of both agents for 4 hr.
(I) Western blot of PARP in cells treated for 24 hr.
(J) Caspase 3/7 DEVDase activity of lysates from HCC1954 cells treated with BYL719 (1 μM), GSK2334470 (1 μM), or the combination of both agents for 12 hr in the presence or absence of caspase inhibitor zVAD-fmk (20 μM). Staurosporine was used as a positive control (1 μM, 4 hr).
(K) HCC1954 xenografts treated with vehicle, BYL719 (25 mg kg^{-1}), GSK2334470 (100 mg kg^{-1}), or the combination of both agents (n = 10/arm).
(L) IHC analysis of tumors from (K) collected at the end of the experiment after 4 hr of the last treatment. Scale bar, 100 μm.
p Values were calculated using Student's t test. Error bars denote ±SEM. See also Figures S1 and S2.

in HCC1954 cells when measured by PARP cleavage (Figure 1I) and caspase 3/7 activity (Figure 1J).

We then expanded our results in vivo. Although some antitumor activity was observed with treatment with BYL719 or GSK2334470, only the combination of both compounds induced durable tumor shrinkage (Figure 1K) and inhibition of pAKT (S473), pS6 (S240/4), and p4EBP1 (T37/46) (Figures 1L and S2L–S2O).

Taken together, these results indicate that PDK1 inhibition sensitizes cells to PI3Kα blockade via suppression of mTORC1.

The PIF-Binding Pocket of PDK1 Is Required for Sustained mTORC1 Activation upon PI3Kα Inhibition

The activation of AGC kinases requires phosphorylation at two highly conserved regulatory motifs termed the hydrophobic

motif (HM) and the activation loop. Several kinases prime AGC kinases for activation through phosphorylation at the HM. PDK1, which acts as a master regulator of this family of kinases, scaffolds at the phosphorylated HM using the PIF (PDK1-interacting fragment) binding pocket. This interaction enables phosphorylation of the activation loop, thereby fully activating their activity. However, AKT does not require the PIF-binding pocket of PDK1 but instead needs its PH domain to interact with PDK1 at the plasma membrane in a PIP_3-dependent manner (Alessi et al., 1997; Arencibia et al., 2013; Biondi et al., 2001; Collins et al., 2003; McManus et al., 2004). To explore the PDK1 regulatory mechanism required to sustain mTORC1 activity upon PI3Kα inhibition, we used the HCT116 parental and *PDPK1*-null (*PDPK1*$^{-/-}$) isogenic model (Ericson et al., 2010). HCT116 cells harbor the H1047R *PIK3CA*-activating mutation, and the addition of BYL719 decreased AKT phosphorylation but did not decrease mTORC1 signaling, mimicking the phenotype observed in BYL719-resistant breast cancer cell lines. However, in *PDPK1*$^{-/-}$ cells, the addition of BYL719 inhibited mTORC1 (Figure S2P).

We reconstituted HCT116 *PDPK1*$^{-/-}$ cells with the wild-type (WT), kinase-inactive K111N (KD), PIP_3-binding deficient K546E (KE), and PIF-pocket-deficient L155E (LE) mutants (Figure S2Q) to test the contribution of each regulatory mechanism of PDK1 to mTORC1 activation. Reconstitution of PDK1 WT, but not the mutant KD, restored mTORC1 activation in the presence of BYL719. The mutant KE was also able to restore the phenotype. On the other hand, the mutant LE was unable to rescue mTORC1 signaling (Figure S2R). This set of experiments suggests that the maintenance of mTORC1 activity requires both kinase activity and the PIF-binding pocket of PDK1 but is PIP_3-and, consequently, AKT-independent.

Combined Suppression of PI3Kα and PDK1 Activates FOXO-Dependent Transcription

We next performed gene-expression analysis to investigate whether mTORC1 suppression is accompanied by specific transcriptional changes. While the differences in gene expression upon BYL719 or GSK2334470 treatment were modest, the combination of both induced marked changes when compared with the DMSO-treated control cells (Figures 2A and S3A). Gene set enrichment analysis of these data showed enrichment of FOXO3 transcription factor targets in both cell lines (Figures 2B and S3B). Individual genes described to be positively (*CCNG2*, *BCL6*, *IRS2*) or negatively (*CCND1*) regulated by FOXO3 (Webb and Brunet, 2014) were confirmed to be induced or repressed, respectively, upon dual PI3Kα and PDK1 blockade (Figure 2C). These results were further validated by performing qRT-PCR of four well-described FOXO3 targets, *ERBB3*, *TNFSF10*, *BCL6*, and *IRS2* (Webb and Brunet, 2014), following different treatments (Figures 2D and S3C).

Upon growth factor stimulation, FOXO transcription factors are phosphorylated at several residues and retained in the cytosol by the 14-3-3 proteins (Webb and Brunet, 2014). Inhibition of these mitogenic signals induces a rapid dephosphorylation and nuclear translocation of FOXOs that allows expression of downstream target genes involved in apoptosis and/or cell-cycle arrest (Webb and Brunet, 2014). In our cells, we found that treatment with both BYL719 and GSK2334470, but not either single agent, resulted in strong nuclear localization of FOXO3 (Figures 2E and S3D). This was consistent with a decreased phosphorylation of this transcription factor at residue T32 (Figure 2F). Moreover, we observed that only the combination stimulated endogenous FOXO transcriptional activity (Figure 2G) and increased occupancy of FOXO3A at the promoters of two well-known FOXO targets, *IRS2* and *TNFSF10* (Figures 2H and S3E). These results suggest that dual PI3Kα and PDK1 inhibition induces a FOXO-dependent transcriptional activity in BYL719-resistant cells.

SGK1 Is Upregulated in BYL719-Resistant Cell Lines and in Tumors from Patients Refractory to PI3Kα Inhibition

AKT has been shown to phosphorylate FOXO1 and FOXO3 at T24 and T32 residues, respectively (Brunet et al., 1999). However, we observed that despite full inhibition of AKT by PI3Kα inhibition, FOXO3 was not efficiently primed to migrate to the nucleus and exert its transcriptional activity in cells resistant to BYL719 (Figures 2 and S3). Since PDK1 requires downstream AGC kinases as molecular effectors (Pearce et al., 2010), we reasoned that in BYL719-resistant cells a downstream AGC kinase dependent on the PDK1 catalytic activity and docking with the PIF-binding pocket (Figure S2R) regulates both FOXO1/3 phosphorylation and mTORC1 activity, independently of AKT.

Serum and glucocorticoid-induced kinase (SGK) is a family of AGC serine/threonine kinases that comprises three members (SGK1–3) highly homologous to AKT (Kobayashi and Cohen, 1999). SGK1 activation is mediated by mTORC2-dependent phosphorylation at the HM (S422) and subsequent PDK1 phosphorylation at the activation loop (T256) in a PIF-binding pocket-dependent manner (Garcia-Martinez and Alessi, 2008; Pearce et al., 2010). Earlier reports have demonstrated that SGK1 is able to directly phosphorylate FOXO1 at residues T32 and S315 (Brunet et al., 2001) and has been correlated with resistance to AKT inhibition (Sommer et al., 2013). Therefore, we hypothesized that SGK1 plays a critical role downstream of PDK1 in sustaining mTORC1 activity and inducing resistance to PI3Kα inhibition.

We analyzed the basal mRNA expression of 27 breast cancer cell lines, previously characterized as sensitive or resistant to BYL719 (Elkabets et al., 2013), and found that resistant cell lines had significantly higher levels of *SGK1* mRNA compared with sensitive cells (Figures 3A and S4A). This held true when only breast cancer cells harboring *PIK3CA*-activating mutations, which are known to be sensitive to PI3Kα inhibition (Elkabets et al., 2013), were considered in the analysis (Figures 3B and 3C). The mRNA levels of *SGK2* and *SGK3* were similar between sensitive and resistant cell lines (Figure S4B), although JIMT1 cells also express high levels of SGK2. The ratio of phosphorylated N-Myc Downstream Regulated 1 (NDRG1) (T346), a substrate of SGK1 (Murray et al., 2004), versus total NDRG1 was also higher in BYL719-resistant cells (Figures 3C and S4C). Both CAL-148 and CAL-51 cells carry mutations in *PTEN* (Cerami et al., 2012), and their resistance to BYL719 may be due to insufficient inhibition of the PI3K/AKT pathway as a consequence of PI3Kβ activity (Juric et al., 2015). However, BYL719, but not the PI3Kβ inhibitor AZD6482, fully decreases pAKT levels in both CAL-148 and CAL-51 cells (Figure S4D).

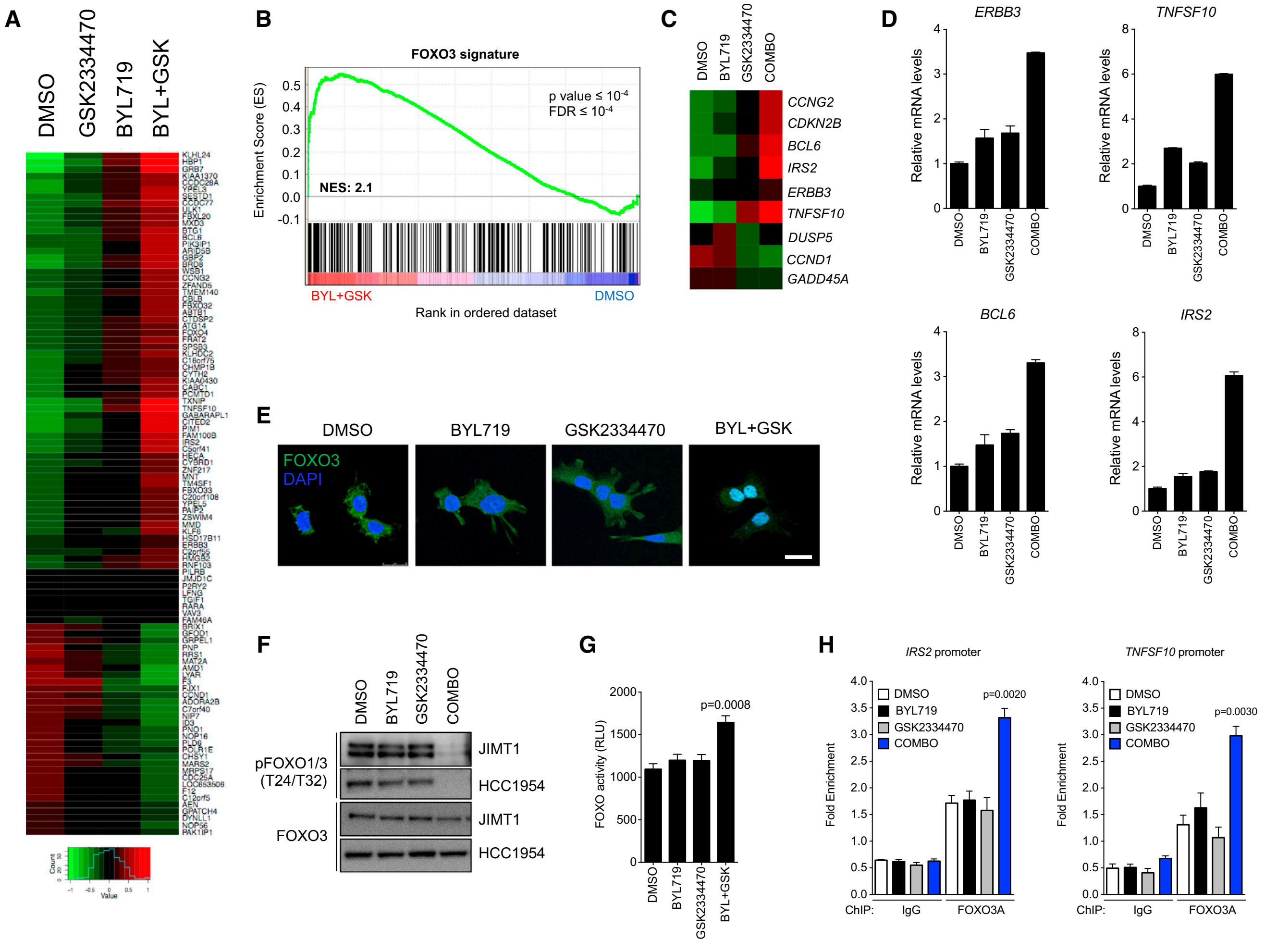

Figure 2. FOXO Activation upon PDK1 and PI3Kα Inhibition

(A) Changes in the top 200 differentially expressed genes in HCC1954 and JIMT1 cells treated with DMSO, BYL719 (1 μM), GSK2334470 (1 μM), or the combination of both agents for 4 hr. Gene upregulation is in red and gene downregulation is in green.

(B) Enrichment plot for the FOXO3 signature in HCC1954 cells. NES, normalized enrichment score.

(C) Heatmap showing changes in expression of FOXO3 targets in HCC1954 and JIMT1 cells.

(D) mRNA expression in HCC1954 cells treated with DMSO, BYL719 (1 μM), GSK2334470 (1 μM), or the combination of both agents for 4 hr.

(E) Representative images of FOXO3 immunofluorescence (green) in HCC1954 cells treated with DMSO, BYL719 (1 μM), GSK2334470 (1 μM), or the combination of both agents for 4 hr. Nuclei are shown in blue (DAPI). Scale bar, 25 μm.

(F) Western blot analysis of FOXO1/3 phosphorylation (T24/T32) in HCC1954 and JIMT1 cells treated with DMSO, BYL719 (1 μM), GSK2334470 (1 μM), or the combination of both agents for 4 hr.

(G) Luciferase reporter assay in HCC1954 cells stably transduced with the FOXO consensus motif reporter construct treated as indicated for 12 hr. RLU, relative light units.

(H) ChIP-qPCR assay of FOXO3A binding at *TNFSF10A* and *IRS2* promoters in HCC1954 cells treated as indicated in (F).

p Values were calculated using Student's t test. Error bars denote ±SEM. See also Figure S3.

Given the lack of reliable results obtained with commercially available antibodies against SGK1, we analyzed the expression of pNDRG1 (T346) in 273 breast invasive carcinomas, comprising 138 triple-negative breast cancer (TNBC), 68 estrogen-/progesterone-receptor-positive, and 67 human epidermal growth factor 2 (HER2)-positive breast cancer patients. High pNDRG1 staining was found in TNBC (21%) and HER2-positive tumors (12%) (Figure 3D), a finding in line with the percentage of breast cancer samples expressing high levels of *SGK1* in the Cancer Genome Atlas cohort (Ciriello et al., 2015).

We then explored whether SGK1 and pNDRG1 expression correlate with clinical outcome to PI3Kα inhibition by analyzing *PIK3CA*-mutant breast cancer samples from 18 patients treated with BYL719 in combination with an aromatase inhibitor (NCT01870505). Three of these tumors expressed high levels of *SGK1* mRNA while the remaining 15 had medium to low levels of *SGK1* mRNA. The three patients with tumors exhibiting high SGK1 expression, which also stained positive for pNDRG1, did not respond to therapy (Figures 3E and 3F). Two patients with tumors expressing medium to low levels of SGK1 stained positive

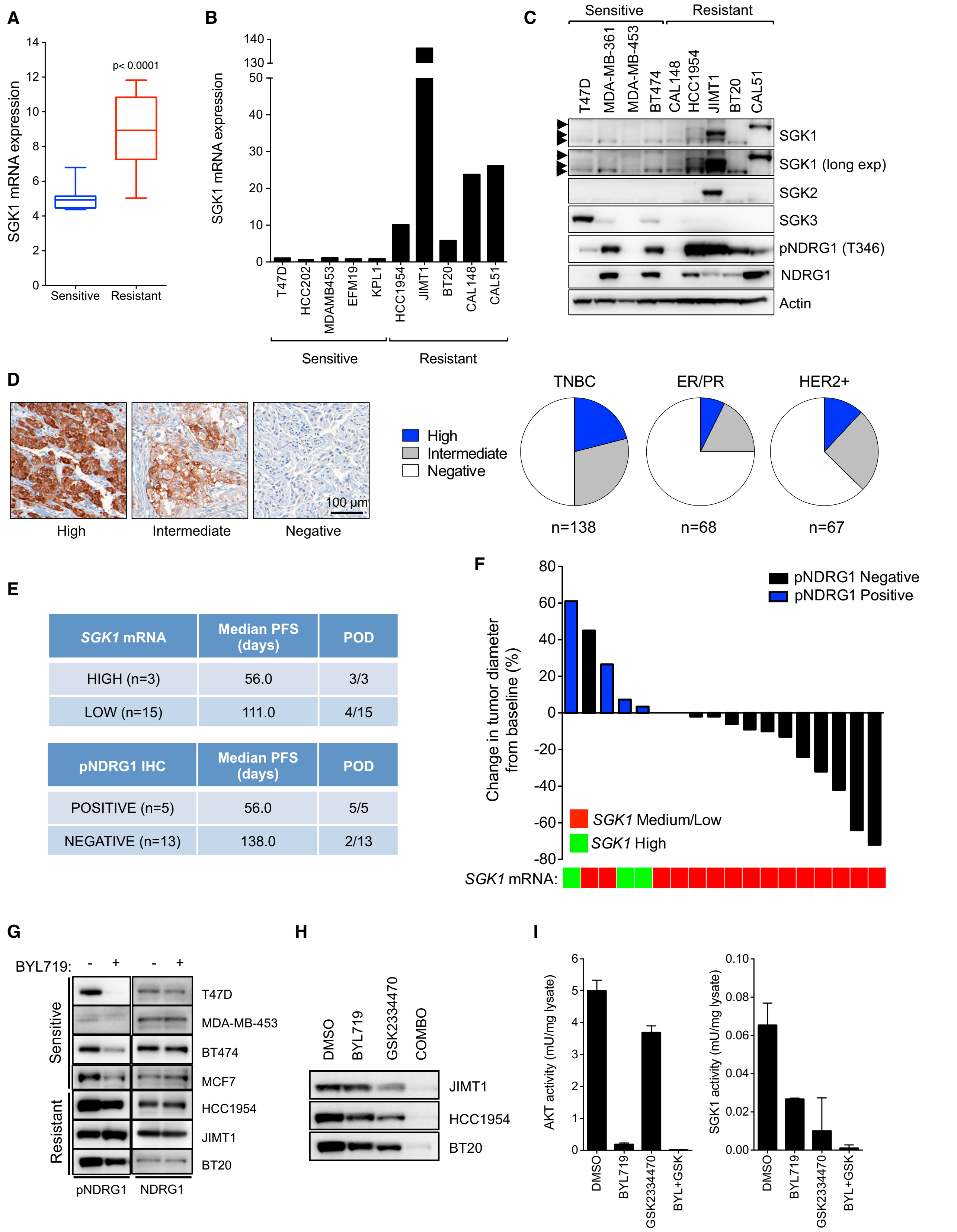

(legend on next page)

for pNDRG1 and rapidly progressed. On the contrary, in the group of patients with pNDRG1-negative tumors, three had partial responses and eight had stable disease by RECIST (response evaluation in solid tumors) criteria (Therasse et al., 2000). This was in agreement with the longer time to disease progression of this subset of patients when compared with the SGK1-high/pNDRG1-positive cohort (Figures 3E and 3F). Although suggestive of a role of SGK1 in mediating intrinsic resistance to PI3Kα inhibitors, these results should be validated in larger cohorts of patients.

We then sought to investigate the mechanism underlying this variability in SGK1 expression. We analyzed the promoter of *SGK1* and realized that in the region between −56 bp and +391 bp of the transcription start site there are 12 CpG sites that are susceptible for DNA methylation. Using bisulfite sequencing we found that three of these CpG sites were differentially methylated between sensitive and resistant cell lines (Figure S4E). We confirmed our results quantitatively using direct pyrosequencing in 11 cell lines (8 sensitive and 3 resistant to PI3Kα inhibition). Sensitive cell lines exhibited high levels of *SGK1* promoter methylation (mean CpG_1 = 65%, CpG_2 = 67%, and CpG_3 = 40%), while resistant cell lines displayed low levels of *SGK1* promoter methylation (mean CpG_1 = 11%, CpG_2 = 13%, and CpG_3 = 16%) (Figure S4F). The degree of promoter DNA methylation inversely correlated with *SGK1* mRNA levels in these cells (Figure S4G). By chromatin immunoprecipitation (ChIP)-qPCR assays, we found high occupancy of RNA polymerase II (Pol II), an enzyme essential for transcription, and phosphorylated (S5) Pol II in both HCC1954 and JIMT1 cells, indicating that *SGK1* transcription is active in these resistant cell lines (Figure S4H). On the contrary, in the sensitive cell lines MDA-MB-453 and T47D we found low occupancy of both Pol II and phosphorylated Pol II (S5) in the *SGK1* promoter (Figure S4H). Treatment with the DNA demethylating agent 5-Aza-2′-deoxycytidine and the histone deacetylase inhibitor panobinostat reduced *SGK1* promoter DNA methylation (data not shown) and increased mRNA levels of *SGK1* in the four sensitive cell lines tested (Figure S4I). Our results indicate that the differential expression of *SGK1* is mediated, at least in part, by epigenetic regulation.

Although pNDRG1 and SGK1 expression correlates in vivo (Murray et al., 2004), AKT can also phosphorylate NDRG1 in the absence of SGK1 in cultured cell lines (Sommer et al., 2013). In support of these observations, cancer cells sensitive to BYL719 displayed decreased NDRG1 phosphorylation at T346 when treated with BYL719 (Figure 3G). In contrast, resistant cell lines treated with BYL719 maintain NDRG1 phosphorylation, underscoring the role of SGK1 in this setting. Central to our work, only the combination of BYL719 and GSK2334470 was able to decrease the phosphorylation of NDRG1 in BYL719-resistant cell lines, confirming that the combination of both drugs is required to effectively inhibit both SGK1 and AKT activity (Figure 3H).

Next, we immunoprecipitated endogenous SGK1 and found that BYL719 treatment was not sufficient to completely abolish the kinase activity of the enzyme, in contrast to GSK2334470 (Figure 3I). On the other hand, immunoprecipitation of endogenous AKT revealed that while BYL719 treatment completely abrogated AKT kinase activity, this is not the case when cells are treated with GSK2334470, as previously observed (Najafov et al., 2012). This is indicative of a signaling compensation between AKT and SGK1 and that only the combination of PI3Kα and PDK1 inhibitors can simultaneously block the activity of the endogenous enzymes in resistant cells. While mTORC2-mediated phosphorylation at the HM is indispensable for SGK1 kinase activity (Kobayashi and Cohen, 1999), several reports indicate that AKT remains active in the absence of HM phosphorylation, as phosphorylation at the activation loop (T308) is sufficient to partially activate the kinase (Guertin et al., 2006; Jacinto et al., 2006; Rodrik-Outmezguine et al., 2011). Treatment of HCC1954 cells with the mTOR catalytic inhibitor AZD8055, which targets both mTORC1 and mTORC2 and completely inhibits SGK1 but not AKT activity, did not reduce the levels of the substrates pFOXO3 (T32) and pNDRG1 (T346), confirming that mTORC2 inhibition is not sufficient to abolish AKT activity in these cells (Figure S4J).

Consistent with previous results, GSK2334470 alone is not capable of inhibiting AKT activity (Figures S4J and 3I). Therefore, in the presence of mTOR inhibition the phosphorylation at the HM is abrogated and this mechanism of AKT activation is no longer supported. Addition of GSK2334470 to resistant cells treated with AZD8055 resulted in a marked decrease in the phosphorylation of both FOXO3 and NDRG1. This translated in decreased cell viability in both HCC1954 and JIMT1 cells,

Figure 3. SGK1 Upregulation in BYL719-Resistant Cell Lines

(A) *SGK1* mRNA levels in breast cancer cell lines sensitive or resistant to BYL719 (n = 27). Box indicates the median and the interquartile range, and whiskers represent minimum and maximum.

(B) *SGK1* mRNA levels in a panel of *PIK3CA*-mutant breast cancer cell lines sensitive or resistant to BYL719.

(C) Western blot analysis of SGK1, SGK2, SGK3, and phosphorylated NDRG1 in a panel of *PIK3CA*-mutant breast cancer cell lines. Arrowheads indicate the SGK1 isoforms.

(D) Representative images of phosphorylated NDRG1 (T346) IHC in breast cancer tumors and quantification of the stainings observed in a cohort of 273 breast cancer cases.

(E) Summary of the median number of days of progression-free survival (PFS) and the number of patients experiencing progression of disease (POD) as best response according to RECIST criteria in association with *SGK1* mRNA levels and positivity to pNDRG1 staining by IHC.

(F) Waterfall plot showing changes in tumor size of the patients included in the study. Heatmap represents the *SGK1* mRNA levels for each tumor sample.

(G) Western blot for NDRG1 and phosphorylated NDRG1 (T346) in BYL719-sensitive and -resistant breast cancer cell lines treated with BYL719 (1 μM) for 4 hr.

(H) Western blot of phosphorylated NDRG1 (T346) in resistant cells treated with DMSO, BYL719 (1 μM), GSK2334470 (1 μM), or the combination of both agents for 4 hr.

(I) Endogenous kinase assay for SGK1 and AKT in HCC1954 cells treated with DMSO, BYL719 (1 μM), GSK2334470 (1 μM), or the combination of both agents for 4 hr.

p Values were calculated using Student's t test. Error bars denote ±SEM. See also Figure S4.

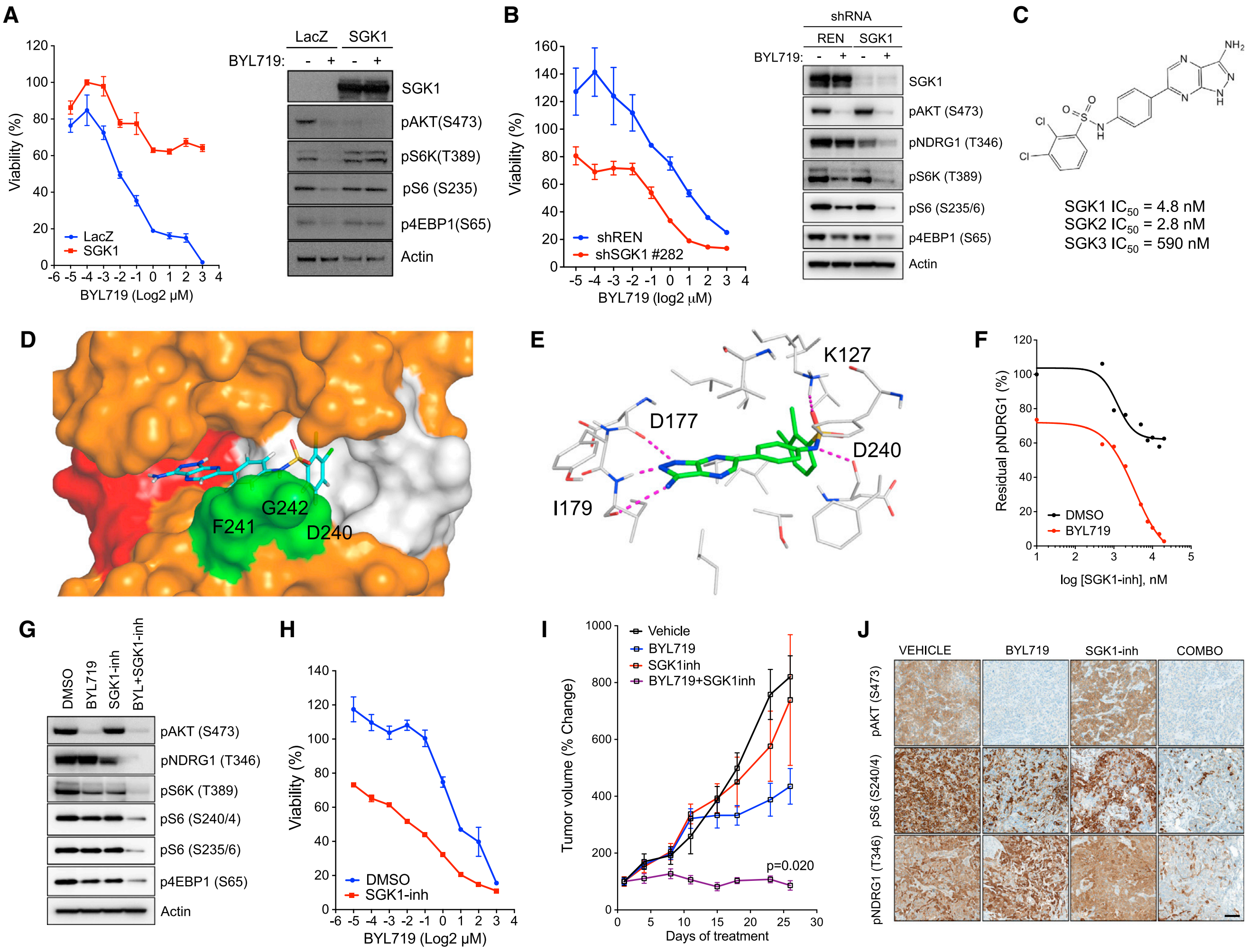

Figure 4. SGK1 Inhibitor Sensitizes Resistant Cells to BYL719

(A) (Left) Dose-response curves from MDA-MB-361 cells transduced with pLenti7.3-LacZ or pLenti7.3-SGK1 (Δ60, S422D) constructs and treated with increasing concentrations of BYL719 for 6 days. (Right) Western blot analysis of LacZ and SGK1 transduced MDA-MB-361 cells treated with BYL719 (1 μM) for 4 hr.

(B) (Left) Growth curves of HCC1954 cells stably expressing doxycycline-inducible control (REN) or SGK1 knockdown treated with increasing concentrations of BYL719 for 6 days. (Right) Western blot analysis of GFP-sorted control (REN) and SGK1 shRNA cells treated with BYL719 (1 μM) for 4 hr.

(C) Chemical structure of SGK1-inh and in vitro SGK1 kinase activity assay in the presence of increasing concentrations of SGK1-inh. IC_{50} for SGK1, SGK2, and SGK3 are indicated.

(D) Docking overview of SGK1-inh in the DFG-out conformation of SGK1. The hinge region is colored in red, the DFG motif in green, the "allosteric" hydrophobic cavity that results from the DFG flip in grey, and the rest of the kinase in orange. The DFG motif amino acids are indicated (D240, F241, and G242).

(E) Detailed residues that mediate the interaction between SGK1-inh and the inactive conformation of SGK1. Hydrogen bonds are shown as purple dotted lines.

(F) Western blot quantification of NDRG1 phosphorylation (T346) in HCC1954 cells treated with increasing concentrations of SGK1-inh for 4 hr in the absence or presence of BYL719 (1 μM).

(G) Western blot analysis of HCC1954 cells treated with BYL719 (1 μM), SGK1-inh (10 μM), or both agents for 4 hr.

(H) Dose-response curves from HCC1954 cells treated with BYL719 for 6 days in the absence or presence of SGK1-inh.

(I) HCC1954 xenograft treated with vehicle, BYL719 (25 mg kg^{-1}), SGK1-inh (50 mg kg^{-1}), or the combination of both agents (n = 10/arm).

(J) IHC analysis of tumors from (K) collected at the end of the experiment 4 hr after the last dosage. Scale bar, 100 μm.

p Values were calculated using Student's t test. Error bars denote ±SEM. See also Figure S5.

phenocopying the effects observed by dual PDK1 and PI3Kα inhibition (Figure S4K). Similar results were found when RICTOR, a key mTORC2 component, was knocked down in the presence of PDK1 inhibition (Figure S4L).

These results demonstrate that both PI3K and PDK1 activities have to be suppressed to inhibit downstream AKT and SGK1 phosphorylation and activity in our resistant models.

SGK1 Mediates Resistance to BYL719

We next assessed the contribution of SGK1 in mediating resistance to PI3Kα inhibition. The overexpression of a constitutively active form of SGK1 in MDA-MB-361 cells, which are sensitive to PI3Kα inhibition, was sufficient to increase cell viability in the presence of BYL719 (Figure 4A). In parental cells, PI3Kα inhibition decreased both AKT phosphorylation and mTORC1

signaling, while cells overexpressing SGK1 maintained mTORC1 signaling in the presence of BYL719. Given that genetic inactivation of SGK1 is toxic (Sommer et al., 2013), we generated doxycycline-inducible shRNA targeting SGK1. Upon SGK1 knockdown we observed a decrease in cell viability that was enhanced in the presence of BYL719 (Figure 4B). Accordingly, SGK1 knockdown decreased pNDRG1 and mTORC1 target levels only when combined with PI3Kα inhibition.

Next, we studied the effects of pharmacological inhibition of SGK1 in our models. The few SGK inhibitors currently available have low activity in cellular models. To overcome this problem, we characterized a recently described SGK inhibitor (SGK1-inh) (Halland et al., 2015). SGK1-inh exhibited an IC_{50} of 4.8 nM at 10 μM ATP using a recombinant SGK1 kinase assay, with appreciable activity also toward SGK2 and SGK3 (IC_{50} of 2.8 nM and 590 nM, respectively) (Figure 4C). The specificity of this compound was tested in vitro at a concentration of 1 μM (200-fold higher than the SGK1 inhibitory dose) against a panel of 140 human kinases showing remarkable selectivity toward SGK1 (Figure S5A). Despite detecting no activity against AKT1, PDK1, PKC, or RSK, we found that at this high concentration S6K was also inhibited, probably due to the high similarity of their catalytic sites. Because S6K is a key downstream substrate of mTORC1, we aimed to further characterize the activity of SGK1-inh toward S6K. Recombinant in vitro kinase assay of S6K demonstrated an IC_{50} of 33 nM, seven times higher than the IC_{50} for SGK1 (Figure S5B). At the cellular level, we performed an S6K kinase assay in 293T cells overexpressing constitutively active S6K (ΔCT T389E) treated with increasing concentrations of SGK1-inh, and found an IC_{50} of ~20 μM (Figure S5C). Next, using two fibroblast cell lines that lack TSC2 (derived from TSC2 knockout mice and a lymphangioleiomyomatosis patient, respectively), we observed that increasing concentrations of SGK1-inh up to 30 μM were not able to reduce S6K signaling in these cellular models, as assessed by the downstream S6K targets pS6 (S235/6), pS6 (S240/4), and pmTOR (S2448) (Figure S5D). This suggests that SGK1-inh does not have activity toward S6K at concentrations below 20–30 μM. We also excluded any potential inhibition of mTORC1 by SGK1-inh by testing this compound against mTOR in a recombinant kinase assay (IC_{50} > 5,000 nM; Figure S5E).

Our computational analyses and preliminary characterization of SGK1-inh suggested that this compound acts as a type II kinase ATP-competitive inhibitor binding preferentially to the inactive conformation of the kinase (Figure 4D). By performing an ATP-competition assay we confirmed that addition of increasing concentrations of ATP decreased the potency of SGK1-inh in a dose-dependent manner (Figure S5F). Docking models using the active conformation of SGK1 show that the sulfonamide moiety with the terminal hydrophobic ring points out from the pocket toward the solvent (Figure S5G), rendering the bound state unstable. In contrast, in the inactive conformation several hydrophobic residues mediate interactions with SGK1-inh within the allosteric DFG-out pocket (mainly V149, L159, V154, and V160; Figure 4E). The pyrazolo(3,4-b)pyrazine head portion of SGK1-inh interacts with the key residues D177 and I179, similar to the interactions with ATP (Figures 4E and S5H). The energetics of SGK1-SGK1-inh binding are more favorable than SGK1-ATP, as assessed by binding free energy calculations (Figure S5I). The electrostatic components of these interactions are similar between ATP and SGK1-inh, and the majority of the binding energy arises from more favorable packing (van der Waals) interactions made between SGK1-inh and the kinase (Figure S5I). Most of the favorable interactions that take place between SGK1 and SGK1-inh are with amino acids found within the SGK1 active site (Figure S5J, upper panel). In silico alanine scanning of the key residues resulted in loss of binding free energies, confirming the importance of these amino acids in the protein-ligand interactions (Figure S5J, lower panel).

Based on the ability to inhibit NDRG1 phosphorylation in the presence of BYL719 in our models (Figure 4F), we estimated that the appropriate concentration of SGK1-inh to fully inhibit endogenous SGK1 is 10 μM. This relatively high concentration (still lower than the concentration needed to affect S6K activity) may be explained by the fact that these sulfonamide derivatives exhibit poor permeability (133×10^{-7} cm s^{-1} in Caco2 cell permeability assays) (Halland et al., 2015). Treatment of HCC1954 and JIMT1 cells with the combination of BYL719 and SGK1-inh not only abrogated pNDRG1 (T346) but also mTORC1 signaling (Figures 4G and S5K). Using m^7GTP pull-downs we also found that combined PI3Kα and SGK1 inhibition induces a decreased cap-dependent translation, as seen by the increased 4EBP1 and decreased eIF4A and eIF4G binding to the m^7GTP beads (Figure S5L). This translated to superior inhibition of cell viability of BYL719-resistant cell lines treated with the combination of BYL719 and SGK1-inh (Figures 4H, S5M, and S5N). We then assessed the potential antitumor activity of SGK1-inh in xenografts. We observed that only the combination of BYL719 and SGK1-inh reduced tumor burden (Figure 4I) and phosphorylation of S6, 4EBP1, and NDRG1 (Figures 4J and S5O).

These results show that targeting SGK1 pharmacologically is feasible, and demonstrate that dual inhibition of AKT and SGK1 is required to achieve full suppression of mTORC1 and proliferation.

SGK1 Interacts with and Phosphorylates TSC2

Due to its similarity with AKT, we reasoned that SGK1 could modulate mTORC1 activity by interacting with a component of the TSC/RHEB/mTORC1 axis. Immunoprecipitation of TSC1, TSC2, RHEB, and mTOR in 293T cells revealed that SGK1 physically interacts with both mTOR and TSC2 proteins (Figures 5A and S6A). While the interaction between SGK1 and mTOR has previously been described, as mTORC2 is responsible for the HM phosphorylation of SGK1 (Garcia-Martinez and Alessi, 2008), to our knowledge the interaction between SGK1 and TSC2 was previously unreported. This result was corroborated in a live cell context by performing fluorescence resonance energy transfer (FRET) experiments using EGFP-tagged TSC2 and EYFP-tagged SGK1 in HeLa cells (Figure 5B). We further confirmed the interaction between endogenous SGK1 and TSC2 by co-immunoprecipitation (Figure 5C). Moreover, we determined the proportion of endogenous SGK1 that is associated with the TSC complex by performing sucrose gradient experiments in JIMT1 lysates. The TSC complex fractionated at high-density fractions (fraction 5), as assessed by the presence of the three components TSC1, TSC2, and TBC1D7 (Figure S6B)

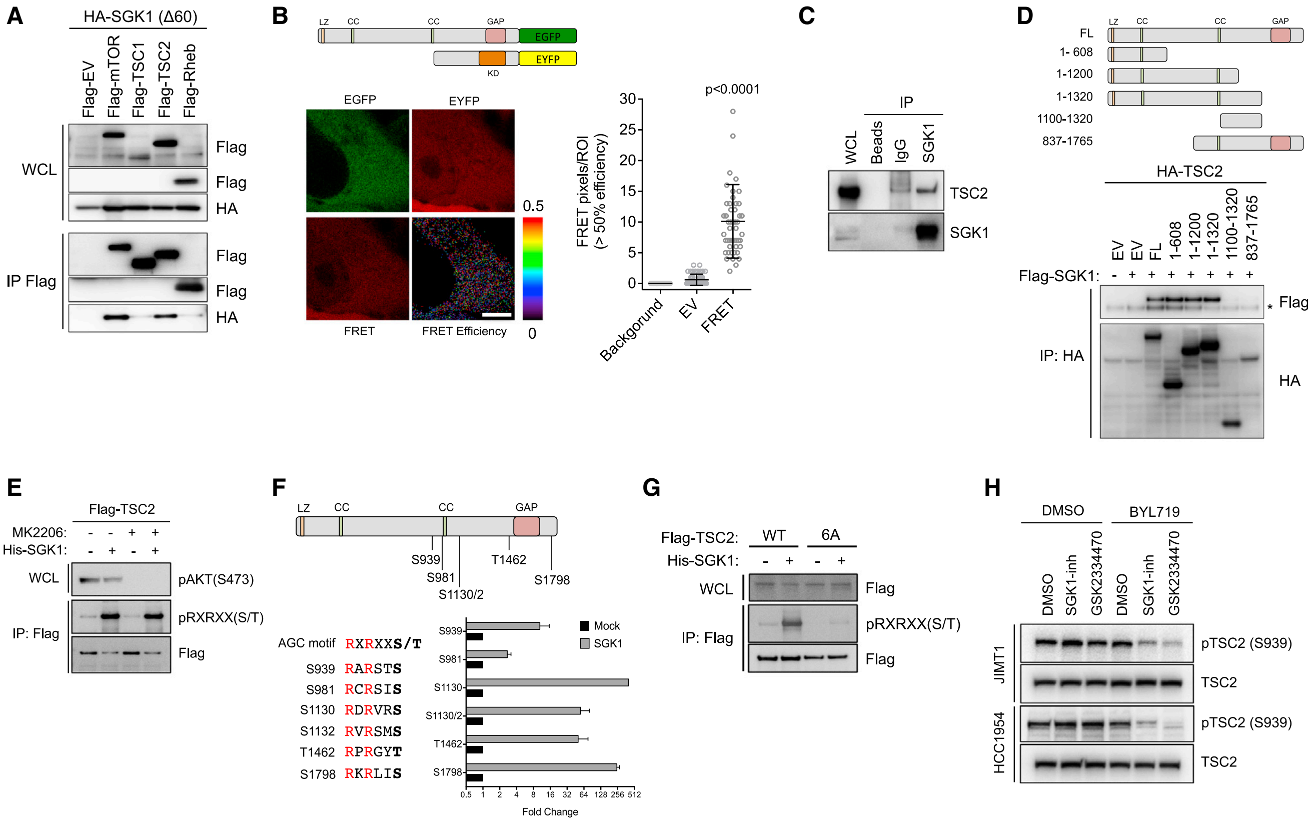

Figure 5. SGK1 Interacts with and Phosphorylates TSC2

(A) Co-immunoprecipitation assay in 293T cells ectopically express the indicated proteins.

(B) Representative efficiency images from the FRET experiment performed in HeLa cells, with the constructs indicated above. Scale bar, 5 μm. Quantification of FRET efficiency dots is shown on the right. ROI, region of interest.

(C) Co-immunoprecipitation (IP) of endogenous SGK1 and TSC2 in JIMT1 cells. IgG, immunoglobulin G.

(D) Co-immunoprecipitation assay in 293T cells between FLAG-SGK1 and truncation mutants of HA-TSC2. Asterisk indicates IgG. Truncation TSC2 mutants are shown schematically at the top. Domains: LZ, leucine zipper; CC, coiled coil; GAP, GTPase activation protein.

(E) In vitro kinase assay using recombinant His-SGK1 and immunoprecipitated FLAG-TSC2 from 293T cells as a substrate (2 μM MK2206, 1 hr).

(F) Quantification of the phosphorylated site identified using liquid chromatography-MS/MS in the absence or presence of recombinant SGK1. Schematic view and amino acid sequence of the predicted SGK1 phosphorylation sites in TSC2 are shown at the top.

(G) In vitro kinase assay using recombinant His-SGK1 and immunoprecipitated and dephosphorylated FLAG-TSC2 WT or 6A as a substrate.

(H) Western blot of phosphorylated TSC2 (S939) in HCC1954 and JIMT1 cells treated with DMSO, BYL719 (1 μM), GSK2334470 (1 μM), SGK1-inh (10 μM), or the combination of both agents for 4 hr.

p Values were calculated using Student's t test. Error bars denote ±SEM. See also Figure S6.

(Dibble et al., 2012). Although most of the SGK1 fractionated at low-molarity fractions, approximately 20% of the kinase eluted at fractions similar to those of the TSC complex. Considering SGK1 as a monomer (or perhaps a dimer [Zhao et al., 2007]), only the association with a larger complex such as the TSC complex can explain the elution at these high sucrose gradients.

Co-immunoprecipitation assays using five different fragments of TSC2 demonstrated that SGK1 binds to the N-terminal region of TSC2 (Figure 5D), which contains a leucine zipper (LZ) domain important for protein-protein interactions and the interaction with TSC1 (Li et al., 2004).

SGK1 has high similarity to AKT in the kinase domain and thus shares many substrates that contain the AGC kinase consensus motif RXRXX(S/T), where R is arginine, X is any amino acid, and (S/T) is a phosphorylatable serine or threonine (Alessi et al., 2009). The use of a degenerated phosphospecific motif antibody allows detection of these phosphosites and has previously been shown to be a reliable surrogate for phospho-TSC2 detection (Manning et al., 2002). When we analyzed the TSC2 protein sequence we found seven putative sites of phosphorylation: S939, S981, T993, S1130, S1132, T1462, and S1798. All of these sites were conserved across several species (Figure S6C). We then established an in vitro kinase assay using recombinant active SGK1 and TSC2 immunoprecipitated from 293T cells as a substrate. To deplete endogenous phosphorylated TSC2, we pre-treated 293T cells with the AKT inhibitor MK2206. The addition of recombinant SGK1 kinase increased the phosphorylation of the RXRXX(S/T) sites of TSC2, even when cells were pre-treated with AKT inhibitor (Figure 5E). Using mass spectrometry (MS), we found increased phosphorylation in all of these sites, except for T993 (Figure 5F). Mutation of these six sites into the non-phosphorylatable amino acid alanine (TSC2 6A) completely

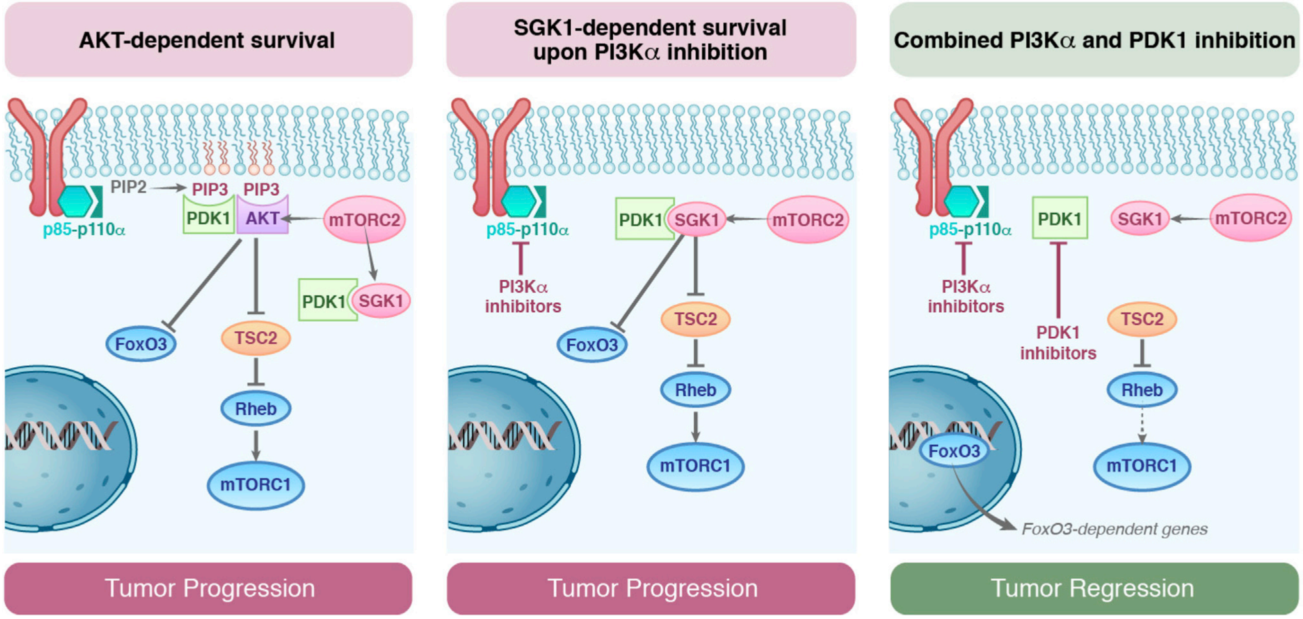

Figure 6. Proposed Model of PI3Kα Resistance in SGK1-Expressing Cells

PIK3CA-mutant breast tumors depend on the PI3K pathway, which mainly signals through AKT. AKT phosphorylates and inhibits FOXO3 and TSC2, promoting mTORC1 activity and tumor progression (left panel). In the presence of PI3Kα inhibitors, PIP_3 levels in the plasma membrane are negligible and AKT cannot be activated. High SGK1 cells become resistant to PI3Kα inhibitors, as SGK1 is not fully inhibited in the presence of these therapies, supporting FOXO3 and TSC2 phosphorylation, which promotes mTORC1 activity and tumor progression (middle panel). When SGK1 expressing cells are treated with PI3Kα and PDK1 inhibitors, both AKT and SGK1 are inhibited, inducing tumor regression as a result of FOXO3 activation and mTORC1 inhibition (right panel).

abrogated the ability of SGK1 to phosphorylate TSC2 in vitro (Figure 5G).

It is well accepted that phosphorylation of these sites increases downstream RHEB-GTP loading and mTORC1 signaling, as a result of a translocation from the lysosome to the cytoplasm (Inoki et al., 2003a; Menon et al., 2014). The phosphorylation and inhibition of TSC2 is phenocopied by the loss of expression of the protein itself, as demonstrated by the induction of mTORC1 activity and consequent resistance to BYL719 in the T47D PI3Kα inhibitor-sensitive cell line depleted of TSC2 (Figures S6D and S6E). To confirm that our biochemical findings are consistent with the proposed mechanism of resistance to BYL719, we treated HCC1954 and JIMT1 cells with BYL719, GSK2334470, SGK1-inh, and the combination of these agents, and found that the phosphorylation of endogenous TSC2 decreases only upon dual PI3Kα and PDK1 or SGK1 suppression (Figure 5H). These results demonstrate that SGK1 can sustain mTORC1 activity in BYL719-resistant cells by phosphorylating and inhibiting the mTORC1-negative regulator TSC2.

We then asked whether kinases other than AKT or SGK1 are involved in the phosphorylation of TSC2 and sustained activation of mTORC1 upon PI3Kα inhibition (Figure S6F). Extracellular Signal-regulated Kinase (ERK) and the downstream AGC kinase RSK phosphorylate TSC2, activating downstream mTORC1 effectors (Ma et al., 2005; Roux et al., 2004). However, we did not detect changes in TSC2 phosphorylation at S939 (or mTORC1 downstream signaling) when HCC1954 cells were treated with the MEK inhibitors PD0325901 and GSK1120212, and downstream ERK and RSK were fully inhibited (Figure S6G). AMP-dependent protein kinase (AMPK), which is activated in conditions of energy stress, phosphorylates TSC2 at S1345 and induces the inhibition of mTORC1 (Inoki et al., 2003b). Treatment of HCC1954 cells with the stress-inducing agent 2-deoxyglucose and the AMPK activator A769662 were unable to rescue the sustained phosphorylation of S6 in this resistant model (Figure S6H). In line with the AMPK regulation of mTORC1 signaling, GSK3 kinase has also been reported to phosphorylate TSC2 using the AMPK-specific site S1345 as a priming event, in a process downstream of WNT signaling (Inoki et al., 2006). However, incubation of HCC1954 cells with the recombinant WNT antagonist DKK-1 did not reduce the sustained S6 phosphorylation (Figure S6I).

Altogether, these results suggest that in our resistant models SGK1 is the main kinase involved in the phosphorylation of TSC2 and sustained mTORC1 activation.

DISCUSSION

In this work, we show that inhibition of the constitutively active kinase PDK1 overcomes resistance to PI3Kα inhibitors in *PIK3CA*-mutant breast cancer cells insensitive to PI3Kα inhibition. We discovered that upon PI3Kα inhibition, and thus low levels of PIP_3 and full suppression of AKT, SGK1 contributes to the maintenance of residual mTORC1 activity and cell survival through direct phosphorylation and inhibition of TSC2. Suppression of either PDK1 or SGK1 prevents mTORC1 activation and sensitizes resistant cells to PI3Kα blockade, underscoring the causative role of this signaling route in inducing the resistance phenotype (Figure 6).

Summarizing our current knowledge, resistance to PI3Kα inhibitors in *PIK3CA*-mutant malignancies may occur as a result of either PI3K-dependent or -independent mechanisms. An example of a PI3K-dependent acquired resistance mechanism has recently been shown by the observation that loss of PTEN results in activation of PI3K p110β, thus forfeiting PI3Kα signaling (Juric et al., 2015). Similarly, reactivation of PI3K p110β signaling has also been revealed to be a mechanism of adaptive resistance in PI3Kα-driven cells (Costa et al., 2015). In terms of PI3K-independent mechanisms, we now propose that mTORC1 sustained activity is, at least in part, mediated by PDK1-SGK1 signaling. In this context, AKT activity would be dispensable for cell survival, in accordance with previous reports showing that AKT activity is not always required for the downstream PI3K signaling (Gasser et al., 2014; Vasudevan et al., 2009).

The role of SGK1 in mediating mTORC1 activation upon PI3Kα inhibition can be explained by the differential regulation of AKT and SGK1 upon pharmacological stress. Although both kinases share the same upstream regulators, mTORC2 and PDK1, AKT contains a PH domain that is required for the PI3K-dependent plasma membrane translocation and subsequent activation. In contrast, SGK1 does not require plasma membrane localization, which could partially explain why it remains active in the absence of PIP_3. In our resistant cell lines treated with PI3Kα inhibitor, we observe a substantial but incomplete decrease in SGK1 activity. This can be partially explained by the fact that PIP_3 controls mTORC2 (Gan et al., 2011) in a mechanism that seems to require mSIN1 (Liu et al., 2015). However, other PIP_3-independent pools of mTORC2 that are not regulated by growth factors (Frias et al., 2006) might be responsible for residual SGK1 activity. While PDK1 is a constitutively active kinase and can be present in both cytoplasm and membrane (upon PIP_3 synthesis), the subcellular localization of mTORC2 is ambiguous (Cybulski and Hall, 2009). Therefore, it is plausible that different pools of mTORC2 can be found within the cell.

Pharmacological inhibition of PDK1 has been reported to have a profound effect on the activity of several AGC kinases such as RSK, S6K, PKC, and SGK (Najafov et al., 2011). However, higher doses of PDK1 inhibitors are required to achieve the same inhibitory effects on AKT. In fact, in the presence of 1 μM GSK2334470 AKT can be efficiently activated by PDK1 through different PIP_3-dependent or PIF-binding pocket-dependent mechanisms (Najafov et al., 2012). In our experiments using endogenous immunoprecipitated SGK1 and AKT, we show that this is indeed the case. In the presence of BYL719, SGK1 but not AKT remains active; conversely, upon GSK2334470 treatment, SGK1 but not AKT is fully inhibited. Single activity of any of these kinases seems to be sufficient to propagate downstream pro-survival signaling through mTORC1 activation and FOXO3 repression. This is also confirmed by the fact that the combination of both agents efficiently inhibits FOXO3 and mTORC1, eliciting a powerful antitumor effect. In this setting, rather than inhibition of AKT, NDRG1 phosphorylation (a substrate of both AKT and SGK1) should be used as readout of pathway inhibition (Kobayashi et al., 1999).

The role of SGK1 in regulating signaling downstream of mTORC2 is intriguing but not entirely unexpected. From the evolutionary point of view, the SGK1 ortholog in *Drosophila melanogaster* is dAkt, which shares similarity with human SGK1 (63%) and AKT1 (67%). Thus, it is tempting to speculate that due to the high overlap of their substrates, *Drosophila* dAkt plays the role of both AKT and SGK1. In fact, Ypk2 and Gad8, the SGK1 orthologs in budding and fission yeast, respectively, are the main TORC2 downstream effectors (Cybulski and Hall, 2009; Kamada et al., 2005; Matsuo et al., 2003). Similarly, in *Caenorhabditis elegans*, sgk-1 appears to be essential for TORC2 signaling, lifespan, and growth (Jones et al., 2009; Soukas et al., 2009).

In summary, our findings show that SGK1 mediates resistance to PI3Kα inhibitors through the activation of mTORC1, which can be reverted by PDK1 blockade. This study highlights the importance of understanding the underlying mechanisms of protein kinase regulation in uncovering critical nodes for pharmacological intervention and improving the therapeutic options for oncogene-driven cancers.

EXPERIMENTAL PROCEDURES

RNAi Screening

JIMT1 and HCC1954 cells were seeded and reverse transfected using Dharmafect-1 with the kinome and phosphatome Ambion Silencer Select v4.0 libraries. Cells were treated with DMSO or 1 μM BYL719, and 7 days after transfection cell viability was assessed. Full details can be found in Supplemental Experimental Procedures.

Immunoblot Detection

Protein lysates were extracted and separated using SDS-PAGE gels according to standard methods. Membranes were probed using specific antibodies. PDK1, pAKT (S473), pAKT (T308), pS6K (T389), pS6 (S240/4), pS6 (235/6), p4EBP1 (S65), PARP, Actin, pRSK (S227), cleaved caspase 3, pFOXO1/3 (T24/T32), SGK1, SGK2, SGK3, pNDRG1 (T346), NDRG1, FLAG, HA, and phospho-RXRXX(S/T) were from Cell Signaling Technology.

Animal Studies

Animals were housed, maintained, and treated at Memorial Sloan Kettering Cancer Center (MSKCC) in accordance with Institutional Animal Care and Use Committee (protocol number 12-10-019). 5×10^6 cells in 1:1 PBS/Matrigel (Corning) were injected subcutaneously into athymic *Foxn1^{nu}* nude mice. When a volume of ~150 mm^3 was reached mice were randomized and treated, and tumors were measured twice per week for 1 month. Full details can be found in Supplemental Experimental Procedures.

Patient Samples

The MSKCC Institutional Review Board approved the study. Pre-treatment formalin-fixed paraffin-embedded blocks from patients treated with the PI3Kα inhibitor BYL719 enrolled in the clinical trial NCT01870505 conducted at MSKCC were used for immunohistochemistry (IHC). Informed consent was obtained from all subjects. Full details of tissue microarray construction are provided in Supplemental Experimental Procedures.

ACCESSION NUMBERS

The microarray data have been deposited in the Gene Expression Omnibus database under accession number GEO: GSE69189.

SUPPLEMENTAL INFORMATION

Supplemental Information includes Supplemental Experimental Procedures, six figures, and one table and can be found with this article online at http://dx.doi.org/10.1016/j.ccell.2016.06.004.

AUTHOR CONTRIBUTIONS

P.C., D.R.A., J.B., and M.S. designed the research. P.C. and H.E. performed the in vitro and in vivo experiments. R.B. performed endogenous kinase

assays. E.T. performed ChIP experiments. S.K. and C.S.V. performed the docking and MS. F.J.C. performed methylation analysis. E.B. scored and quantified IHC experiments. P.R., S. C., and M.D. provided patient samples. P.C. prepared the figures. P.C., M.S., and J.B. wrote the manuscript.

ACKNOWLEDGMENTS

We are grateful to M. Nazare and N. Halland who provided the SGK1 inhibitor. We thank H. Djaballah, D. Shum, and B. Bhinder for help with the siRNA screening, R. Soni and R. Hendrickson for help with mass spectrometry, V. Boyko for help with FRET, C. Jones for microarray analysis, M. Asher for help with IHC, and X. Le and L. Garraway for help with *SGK1* expression analysis. This work has been supported by NIH grants P30 CA008748, R01CA190642-01A1, the Breast Cancer Research Foundation, the Geoffrey Beene Cancer Research Center, the A*CRC (A*STAR) and the BMSI (A*STAR) Singapore. D.R.A. and R.B. are supported by the Medical Research Council (MC_UU_12016/2). F.J.C. and E.T. are Terri Brodeur Foundation fellows.

Received: July 24, 2015
Revised: January 27, 2016
Accepted: June 9, 2016
Published: July 21, 2016

REFERENCES

Alessi, D.R., James, S.R., Downes, C.P., Holmes, A.B., Gaffney, P.R., Reese, C.B., and Cohen, P. (1997). Characterization of a 3-phosphoinositide-dependent protein kinase which phosphorylates and activates protein kinase Balpha. Curr. Biol. *7*, 261–269.

Alessi, D.R., Pearce, L.R., and Garcia-Martinez, J.M. (2009). New insights into mTOR signaling: mTORC2 and beyond. Sci. Signal. *2*, pe27.

Arencibia, J.M., Pastor-Flores, D., Bauer, A.F., Schulze, J.O., and Biondi, R.M. (2013). AGC protein kinases: from structural mechanism of regulation to allosteric drug development for the treatment of human diseases. Biochim. Biophys. Acta *1834*, 1302–1321.

Biondi, R.M., Kieloch, A., Currie, R.A., Deak, M., and Alessi, D.R. (2001). The PIF-binding pocket in PDK1 is essential for activation of S6K and SGK, but not PKB. EMBO J. *20*, 4380–4390.

Brunet, A., Bonni, A., Zigmond, M.J., Lin, M.Z., Juo, P., Hu, L.S., Anderson, M.J., Arden, K.C., Blenis, J., and Greenberg, M.E. (1999). Akt promotes cell survival by phosphorylating and inhibiting a Forkhead transcription factor. Cell *96*, 857–868.

Brunet, A., Park, J., Tran, H., Hu, L.S., Hemmings, B.A., and Greenberg, M.E. (2001). Protein kinase SGK mediates survival signals by phosphorylating the forkhead transcription factor FKHRL1 (FOXO3a). Mol. Cell Biol. *21*, 952–965.

Cerami, E., Gao, J., Dogrusoz, U., Gross, B.E., Sumer, S.O., Aksoy, B.A., Jacobsen, A., Byrne, C.J., Heuer, M.L., Larsson, E., et al. (2012). The cBio cancer genomics portal: an open platform for exploring multidimensional cancer genomics data. Cancer Discov. *2*, 401–404.

Ciriello, G., Gatza, M.L., Beck, A.H., Wilkerson, M.D., Rhie, S.K., Pastore, A., Zhang, H., McLellan, M., Yau, C., Kandoth, C., et al. (2015). Comprehensive molecular portraits of invasive lobular breast cancer. Cell *163*, 506–519.

Collins, B.J., Deak, M., Arthur, J.S., Armit, L.J., and Alessi, D.R. (2003). In vivo role of the PIF-binding docking site of PDK1 defined by knock-in mutation. EMBO J. *22*, 4202–4211.

Costa, C., Ebi, H., Martini, M., Beausoleil, S.A., Faber, A.C., Jakubik, C.T., Huang, A., Wang, Y., Nishtala, M., Hall, B., et al. (2015). Measurement of PIP3 levels reveals an unexpected role for p110beta in early adaptive responses to p110alpha-specific inhibitors in luminal breast cancer. Cancer Cell *27*, 97–108.

Currie, R.A., Walker, K.S., Gray, A., Deak, M., Casamayor, A., Downes, C.P., Cohen, P., Alessi, D.R., and Lucocq, J. (1999). Role of phosphatidylinositol 3,4,5-trisphosphate in regulating the activity and localization of 3-phosphoinositide-dependent protein kinase-1. Biochem. J. *337*, 575–583.

Cybulski, N., and Hall, M.N. (2009). TOR complex 2: a signaling pathway of its own. Trends Biochem. Sci. *34*, 620–627.

Dibble, C.C., Elis, W., Menon, S., Qin, W., Klekota, J., Asara, J.M., Finan, P.M., Kwiatkowski, D.J., Murphy, L.O., and Manning, B.D. (2012). TBC1D7 is a third subunit of the TSC1-TSC2 complex upstream of mTORC1. Mol. Cell *47*, 535–546.

Elkabets, M., Vora, S., Juric, D., Morse, N., Mino-Kenudson, M., Muranen, T., Tao, J., Campos, A.B., Rodon, J., Ibrahim, Y.H., et al. (2013). mTORC1 inhibition is required for sensitivity to PI3K p110alpha inhibitors in PIK3CA-mutant breast cancer. Sci. Transl. Med. *5*, 196ra199.

Engelman, J.A. (2009). Targeting PI3K signalling in cancer: opportunities, challenges and limitations. Nat. Rev. Cancer *9*, 550–562.

Ericson, K., Gan, C., Cheong, I., Rago, C., Samuels, Y., Velculescu, V.E., Kinzler, K.W., Huso, D.L., Vogelstein, B., and Papadopoulos, N. (2010). Genetic inactivation of AKT1, AKT2, and PDPK1 in human colorectal cancer cells clarifies their roles in tumor growth regulation. Proc. Natl. Acad. Sci. USA *107*, 2598–2603.

Frias, M.A., Thoreen, C.C., Jaffe, J.D., Schroder, W., Sculley, T., Carr, S.A., and Sabatini, D.M. (2006). mSin1 is necessary for Akt/PKB phosphorylation, and its isoforms define three distinct mTORC2s. Curr. Biol. *16*, 1865–1870.

Fruman, D.A., and Rommel, C. (2014). PI3K and cancer: lessons, challenges and opportunities. Nat. Rev. Drug Discov. *13*, 140–156.

Gan, X., Wang, J., Su, B., and Wu, D. (2011). Evidence for direct activation of mTORC2 kinase activity by phosphatidylinositol 3,4,5-trisphosphate. J. Biol. Chem. *286*, 10998–11002.

Garcia-Martinez, J.M., and Alessi, D.R. (2008). mTOR complex 2 (mTORC2) controls hydrophobic motif phosphorylation and activation of serum- and glucocorticoid-induced protein kinase 1 (SGK1). Biochem. J. *416*, 375–385.

Gasser, J.A., Inuzuka, H., Lau, A.W., Wei, W., Beroukhim, R., and Toker, A. (2014). SGK3 mediates INPP4B-dependent PI3K signaling in breast cancer. Mol. Cell *56*, 595–607.

Guertin, D.A., Stevens, D.M., Thoreen, C.C., Burds, A.A., Kalaany, N.Y., Moffat, J., Brown, M., Fitzgerald, K.J., and Sabatini, D.M. (2006). Ablation in mice of the mTORC components raptor, rictor, or mLST8 reveals that mTORC2 is required for signaling to Akt-FOXO and PKCalpha, but not S6K1. Dev. Cell *11*, 859–871.

Halland, N., Schmidt, F., Weiss, T., Saas, J., Li, Z., Czech, J., Dreyer, M., Hofmeister, A., Mertsch, K., Dietz, U., et al. (2015). Discovery of N-[4-(1H-Pyrazolo[3,4-b]pyrazin-6-yl)-phenyl]-sulfonamides as highly active and selective SGK1 inhibitors. ACS Med. Chem. Lett. *6*, 73–78.

Inoki, K., Li, Y., Zhu, T., Wu, J., and Guan, K.L. (2002). TSC2 is phosphorylated and inhibited by Akt and suppresses mTOR signalling. Nat. Cell Biol. *4*, 648–657.

Inoki, K., Li, Y., Xu, T., and Guan, K.L. (2003a). Rheb GTPase is a direct target of TSC2 GAP activity and regulates mTOR signaling. Genes Dev. *17*, 1829–1834.

Inoki, K., Zhu, T., and Guan, K.L. (2003b). TSC2 mediates cellular energy response to control cell growth and survival. Cell *115*, 577–590.

Inoki, K., Ouyang, H., Zhu, T., Lindvall, C., Wang, Y., Zhang, X., Yang, Q., Bennett, C., Harada, Y., Stankunas, K., et al. (2006). TSC2 integrates Wnt and energy signals via a coordinated phosphorylation by AMPK and GSK3 to regulate cell growth. Cell *126*, 955–968.

Jacinto, E., Facchinetti, V., Liu, D., Soto, N., Wei, S., Jung, S.Y., Huang, Q., Qin, J., and Su, B. (2006). SIN1/MIP1 maintains rictor-mTOR complex integrity and regulates Akt phosphorylation and substrate specificity. Cell *127*, 125–137.

Jones, K.T., Greer, E.R., Pearce, D., and Ashrafi, K. (2009). Rictor/TORC2 regulates *Caenorhabditis elegans* fat storage, body size, and development through sgk-1. PLoS Biol. *7*, e60.

Juric, D., Rodon, J., Gonzalez-Angulo, A.M., Burris, H.A., Bendell, J., Berlin, J.D., Middleton, M.R., Bootle, D., Boehm, M., Schmitt, A., et al. (2012). BYL719, a next generation PI3K alpha specific inhibitor: preliminary safety, PK, and efficacy results from the first-in-human study. Cancer Res. *72*, CT-01.

Juric, D., Krop, I., Ramanathan, R.K., Xiao, J., Sanabria, S., Wilson, T.R., Choi, Y., Parmar, H., Hsu, J., Baselga, J., and Von Hoff, D.D. (2013). GDC-0032, a beta isoform-sparing PI3K inhibitor: results of a first-in-human phase Ia dose escalation study. Cancer Res. *73*, LB-64.

Juric, D., Castel, P., Griffith, M., Griffith, O.L., Won, H.H., Ellis, H., Ebbesen, S.H., Ainscough, B.J., Ramu, A., Iyer, G., et al. (2015). Convergent loss of PTEN leads to clinical resistance to a PI(3)Kalpha inhibitor. Nature *518*, 240–244.

Kamada, Y., Fujioka, Y., Suzuki, N.N., Inagaki, F., Wullschleger, S., Loewith, R., Hall, M.N., and Ohsumi, Y. (2005). Tor2 directly phosphorylates the AGC kinase Ypk2 to regulate actin polarization. Mol. Cell. Biol. *25*, 7239–7248.

Kobayashi, T., and Cohen, P. (1999). Activation of serum- and glucocorticoid-regulated protein kinase by agonists that activate phosphatidylinositide 3-kinase is mediated by 3-phosphoinositide-dependent protein kinase-1 (PDK1) and PDK2. Biochem. J. *339*, 319–328.

Kobayashi, T., Deak, M., Morrice, N., and Cohen, P. (1999). Characterization of the structure and regulation of two novel isoforms of serum- and glucocorticoid-induced protein kinase. Biochem. J. *344*, 189–197.

Li, Y., Corradetti, M.N., Inoki, K., and Guan, K.L. (2004). TSC2: filling the GAP in the mTOR signaling pathway. Trends Biochem. Sci. *29*, 32–38.

Liu, P., Gan, W., Chin, Y.R., Ogura, K., Guo, J., Zhang, J., Wang, B., Blenis, J., Cantley, L.C., Toker, A., et al. (2015). PtdIns(3,4,5)P3-dependent activation of the mTORC2 kinase complex. Cancer Discov. *5*, 1194–1209.

Ma, L., Chen, Z., Erdjument-Bromage, H., Tempst, P., and Pandolfi, P.P. (2005). Phosphorylation and functional inactivation of TSC2 by Erk implications for tuberous sclerosis and cancer pathogenesis. Cell *121*, 179–193.

Manning, B.D., and Cantley, L.C. (2007). AKT/PKB signaling: navigating downstream. Cell *129*, 1261–1274.

Manning, B.D., Tee, A.R., Logsdon, M.N., Blenis, J., and Cantley, L.C. (2002). Identification of the tuberous sclerosis complex-2 tumor suppressor gene product tuberin as a target of the phosphoinositide 3-kinase/akt pathway. Mol. Cell *10*, 151–162.

Matsuo, T., Kubo, Y., Watanabe, Y., and Yamamoto, M. (2003). Schizosaccharomyces pombe AGC family kinase Gad8p forms a conserved signaling module with TOR and PDK1-like kinases. EMBO J. *22*, 3073–3083.

McManus, E.J., Collins, B.J., Ashby, P.R., Prescott, A.R., Murray-Tait, V., Armit, L.J., Arthur, J.S., and Alessi, D.R. (2004). The in vivo role of PtdIns(3,4,5)P3 binding to PDK1 PH domain defined by knockin mutation. EMBO J. *23*, 2071–2082.

Menon, S., Dibble, C.C., Talbott, G., Hoxhaj, G., Valvezan, A.J., Takahashi, H., Cantley, L.C., and Manning, B.D. (2014). Spatial control of the TSC complex integrates insulin and nutrient regulation of mTORC1 at the lysosome. Cell *156*, 771–785.

Murray, J.T., Campbell, D.G., Morrice, N., Auld, G.C., Shpiro, N., Marquez, R., Peggie, M., Bain, J., Bloomberg, G.B., Grahammer, F., et al. (2004). Exploitation of KESTREL to identify NDRG family members as physiological substrates for SGK1 and GSK3. Biochem. J. *384*, 477–488.

Najafov, A., Sommer, E.M., Axten, J.M., Deyoung, M.P., and Alessi, D.R. (2011). Characterization of GSK2334470, a novel and highly specific inhibitor of PDK1. Biochem. J. *433*, 357–369.

Najafov, A., Shpiro, N., and Alessi, D.R. (2012). Akt is efficiently activated by PIF-pocket- and PtdIns(3,4,5)P3-dependent mechanisms leading to resistance to PDK1 inhibitors. Biochem. J. *448*, 285–295.

Pearce, L.R., Komander, D., and Alessi, D.R. (2010). The nuts and bolts of AGC protein kinases. Nat. Rev. Mol. Cell Biol. *11*, 9–22.

Potter, C.J., Pedraza, L.G., and Xu, T. (2002). Akt regulates growth by directly phosphorylating Tsc2. Nat. Cell Biol. *4*, 658–665.

Rodrik-Outmezguine, V.S., Chandarlapaty, S., Pagano, N.C., Poulikakos, P.I., Scaltriti, M., Moskatel, E., Baselga, J., Guichard, S., and Rosen, N. (2011). mTOR kinase inhibition causes feedback-dependent biphasic regulation of AKT signaling. Cancer Discov. *1*, 248–259.

Roux, P.P., Ballif, B.A., Anjum, R., Gygi, S.P., and Blenis, J. (2004). Tumor-promoting phorbol esters and activated Ras inactivate the tuberous sclerosis tumor suppressor complex via p90 ribosomal S6 kinase. Proc. Natl. Acad. Sci. USA *101*, 13489–13494.

Sancak, Y., Thoreen, C.C., Peterson, T.R., Lindquist, R.A., Kang, S.A., Spooner, E., Carr, S.A., and Sabatini, D.M. (2007). PRAS40 is an insulin-regulated inhibitor of the mTORC1 protein kinase. Mol. Cell *25*, 903–915.

Sarbassov, D.D., Guertin, D.A., Ali, S.M., and Sabatini, D.M. (2005). Phosphorylation and regulation of Akt/PKB by the rictor-mTOR complex. Science *307*, 1098–1101.

Silvera, D., Formenti, S.C., and Schneider, R.J. (2010). Translational control in cancer. Nat. Rev. Cancer *10*, 254–266.

Sommer, E.M., Dry, H., Cross, D., Guichard, S., Davies, B.R., and Alessi, D.R. (2013). Elevated SGK1 predicts resistance of breast cancer cells to Akt inhibitors. Biochem. J. *452*, 499–508.

Soukas, A.A., Kane, E.A., Carr, C.E., Melo, J.A., and Ruvkun, G. (2009). Rictor/TORC2 regulates fat metabolism, feeding, growth, and life span in *Caenorhabditis elegans*. Genes Dev. *23*, 496–511.

Therasse, P., Arbuck, S.G., Eisenhauer, E.A., Wanders, J., Kaplan, R.S., Rubinstein, L., Verweij, J., Van Glabbeke, M., van Oosterom, A.T., Christian, M.C., et al. (2000). New guidelines to evaluate the response to treatment in solid tumors. European Organization for Research and Treatment of Cancer, National Cancer Institute of the United States, National Cancer Institute of Canada. J. Natl. Cancer Inst. *92*, 205–216.

Thorpe, L.M., Yuzugullu, H., and Zhao, J.J. (2015). PI3K in cancer: divergent roles of isoforms, modes of activation and therapeutic targeting. Nat. Rev. Cancer *15*, 7–24.

Vasudevan, K.M., Barbie, D.A., Davies, M.A., Rabinovsky, R., McNear, C.J., Kim, J.J., Hennessy, B.T., Tseng, H., Pochanard, P., Kim, S.Y., et al. (2009). AKT-independent signaling downstream of oncogenic PIK3CA mutations in human cancer. Cancer Cell *16*, 21–32.

Webb, A.E., and Brunet, A. (2014). FOXO transcription factors: key regulators of cellular quality control. Trends Biochem. Sci. *39*, 159–169.

Zhao, B., Lehr, R., Smallwood, A.M., Ho, T.F., Maley, K., Randall, T., Head, M.S., Koretke, K.K., and Schnackenberg, C.G. (2007). Crystal structure of the kinase domain of serum and glucocorticoid-regulated kinase 1 in complex with AMP PNP. Protein Sci. *16*, 2761–2769.

Cancer Cell, Volume *30*

Supplemental Information

PDK1-SGK1 Signaling Sustains AKT-Independent mTORC1 Activation and Confers Resistance to PI3Kα Inhibition

Pau Castel, Haley Ellis, Ruzica Bago, Eneda Toska, Pedram Razavi, F. Javier Carmona, Srinivasaraghavan Kannan, Chandra S. Verma, Maura Dickler, Sarat Chandarlapaty, Edi Brogi, Dario R. Alessi, José Baselga, and Maurizio Scaltriti

Supplemental Data

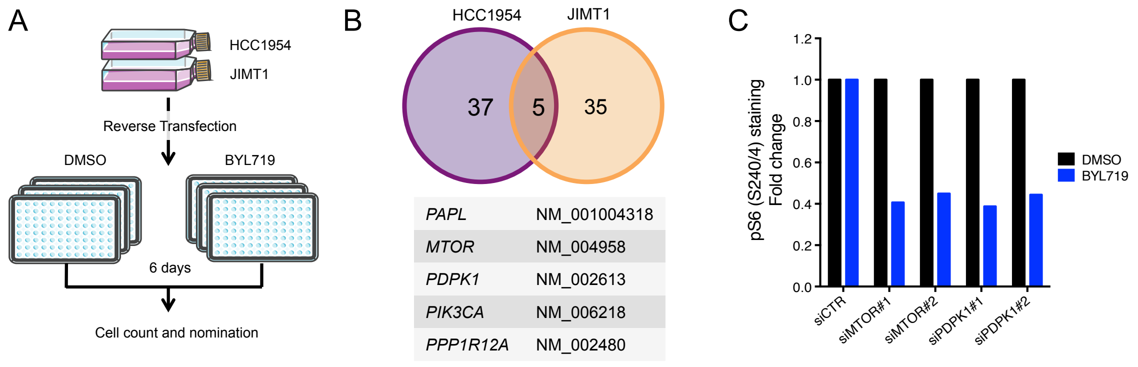

Figure S1. Relative to Figure 1

(A) Overview of the large screening carried out in this work using libraries against the human kinome and phosphatome. **(B)** Venn diagram indicating the number of genes found to sensitize to BYL719 treatment (1 μM) in each cell line and in common. The table contains the gene name and NCBI mRNA accession number targeted by the siRNA found to sensitize both cell lines to BYL719. **(C)** Quantification of pS6 (S240/4) staining in the siCTR, siMTOR, and siPDK1 transfected cells in the presence of DMSO or BYL719 (1 μM) in JIMT1 cells. Quantification of the green fluorescence from the whole well is indicated as a fold change of the control untreated cells.

Table S1. Relative to Figure 1. Genes identified in the siRNA screening

HCC1954 cell line

Gene Symbol	RefSeq ID	Gene Description	H score (Drug)	Validated viability	pS6 (S240/4) staining
AKAP8	NM_005858	A kinase (PRKA) anchor protein 8	67		
CAMKK2	NM_006549	calcium/calmodulin-dependent protein kinase kinase 2, beta	67		
CHEK1	NM_001274	CHK1 checkpoint homolog	67		
DUSP10	NM_007207	dual specificity phosphatase 10	67		
ERBB3	NM_001005915	v-erb-b2 erythroblastic leukemia viral oncogene homolog 3	67		
FBP1	NM_000507	fructose-1,6-bisphosphatase 1	67		
FER	NM_005246	fer (fps/fes related) tyrosine kinase	67		
PAPL	NM_001004318	purple acid phosphatase long form	67	NO	NO
FLT1	NM_002019	fms-related tyrosine kinase 1 (vascular endothelial growth factor)	67		
MTOR	NM_004958	FK506 binding protein 12-rapamycin associated protein 1	67	**YES**	**YES**
HDHD1A	NM_012080	haloacid dehalogenase-like hydrolase domain containing 1A	67		
INPPL1	NM_001567	inositol polyphosphate phosphatase-like 1	100		
IRAK4	NM_016123	interleukin-1 receptor-associated kinase 4	67		
JAK1	NM_002227	Janus kinase 1	67		
MAP2K3	NM_002756	mitogen-activated protein kinase kinase 3	100		
MASTL	NM_032844	microtubule associated serine/threonine kinase-like	67		
MPP5	NM_022474	membrane protein, palmitoylated 5	67		
MPP7	NM_173496	membrane protein, palmitoylated 7	67		
MTMR1	NM_003828	myotubularin related protein 1	100		
MTMR3	NM_021090	myotubularin related protein 3	67		
MTMR6	NM_004685	myotubularin related protein 6	67		
MTMR8	NM_017677	myotubularin related protein 8	67		
NAP1L1	NM_004537	nucleosome assembly protein 1-like 1	100		
NAP1L4	NM_005969	nucleosome assembly protein 1-like 4	67		
NME4	NM_005009	non-metastatic cells 4	67		
PDGFRA	NM_006206	platelet-derived growth factor receptor, alpha polypeptide	67		
PDPK1	NM_002613	3-phosphoinositide dependent protein kinase-1	100	**YES**	**YES**
PIK3CA	NM_006218	phosphoinositide-3-kinase	100	NO	NO
PIP5KL1	NM_173492	phosphatidylinositol-4-phosphate 5-kinase-like 1	67		
PLK3	NM_004073	polo-like kinase 3	100		
PPAP2B	NM_003713	phosphatidic acid phosphatase type 2B	67		
PPP1R12A	NM_002480	protein phosphatase 1, regulatory (inhibitor) subunit 12A	67	NO	NO
PPP1R12B	NM_032104	protein phosphatase 1, regulatory (inhibitor) subunit 12B	100		
PPP1R8	NM_002713	protein phosphatase 1, regulatory (inhibitor) subunit 8	67		
PPP6C	NM_002721	protein phosphatase 6, catalytic subunit	67		
PTP4A1	NM_003463	protein tyrosine phosphatase type IVA	67		
PTPN13	NM_006264	protein tyrosine phosphatase, non-receptor type 13	67		
PXK	NM_017771	PX domain containing serine/threonine kinase	67		
RBKS	NM_022128	ribokinas	67		
SNRK	NM_017719	SNF related kinase	67		
TNK1	NM_003985	tyrosine kinase, non-receptor	67		
WNK2	NM_006648	WNK lysine deficient protein kinase 2	67		

JIMT1 cell line

Gene Symbol	RefSeq ID	Gene Description	H score (Drug)	Validated viability	pS6 (S240/4) staining
ACVRL1	NM_000020	activin A receptor type II-like 1	67		
AKT1	NM_001014431	v-akt murine thymoma viral oncogene homolog 1	67		
ALPI	NM_001631	alkaline phosphatase, intestinal	67		
ALPL	NM_000478	alkaline phosphatase, liver/bone/kidney	67		
ARAF	NM_001654	v-raf murine sarcoma 3611 viral oncogene homolog	67		
CDC42BPA	NM_003607	CDC42 binding protein kinase alpha	67		
CDK2	NM_001798	cyclin-dependent kinase 2	67		
CDK4	NM_000075	cyclin-dependent kinase 4	100		
CDK9	NM_001261	cyclin-dependent kinase 9 (CDK9), mRNA	67		
CSNK1A1	NM_001025105	casein kinase 1, alpha 1	67		
CSNK1A1L	NM_145203	casein kinase 1, alpha 1-like	67		
CSNK1G2	NM_001319	casein kinase 1, gamma 2	67		
ENTPD2	NM_001246	ectonucleoside triphosphate diphosphohydrolase 2	67		
EPHA3	NM_005233	EPH receptor A3 (EPHA3), transcript variant 1, mRNA	67		
EPHA5	NM_004439	EPH receptor A5	67		
EPHB2	NM_004442	EPH receptor B2	67		
PAPL	NM_001004318	purple acid phosphatase long form	67	NO	NO
MTOR	NM_004958	FK506 binding protein 12-rapamycin associated protein 1	67	**YES**	**YES**
GK5	NM_001039547	glycerol kinase 5	67		
IGFN1	NM_178275	immunoglobulin-like and fibronectin type III domain containing 1	67		
ITGB1BP3	NM_170678	integrin beta 1 binding protein 3	67		
LRRK2	NM_198578	leucine-rich repeat kinase 2	67		
LTK	NM_002344	leukocyte receptor tyrosine kinase	67		
MAP2K2	NM_030662	mitogen-activated protein kinase kinase	67		
MST1R	NM_002447	macrophage stimulating 1 receptor	67		
NEK3	NM_002498	NIMA (never in mitosis gene a)-related kinase 3	67		
NEK7	NM_133494	NIMA (never in mitosis gene a)-related kinase 7	67		
PDK2	NM_002611	pyruvate dehydrogenase kinase, isozyme 2	67		
PDPK1	NM_002613	3-phosphoinositide dependent protein kinase-1	67	**YES**	**YES**
PIK3CA	NM_006218	phosphoinositide-3-kinase, catalytic, alpha polypeptide	67, 100	NO	NO
PPP1CA	NM_001008709	protein phosphatase 1, catalytic subunit, alpha isoform	67		
PPP1R12A	NM_002480	protein phosphatase 1, regulatory (inhibitor) subunit 12A	67	NO	NO
PPP1R3F	NM_033215	protein phosphatase 1, regulatory (inhibitor) subunit 3F	67		
PPP2R5D	NM_006245	protein phosphatase 2, regulatory subunit B', delta isoform	67		
PRKD2	NM_001079880	protein kinase D2	67		
PTPRF	NM_002840	protein tyrosine phosphatase, receptor type, F	67		
PTPRT	NM_007050	protein tyrosine phosphatase, receptor type, T	67		
TESK1	NM_006285	testis-specific kinase 1	67		
TRIB1	NM_025195	tribbles homolog 1	67		
WNK1	NM_018979	WNK lysine deficient protein kinase 1	67		

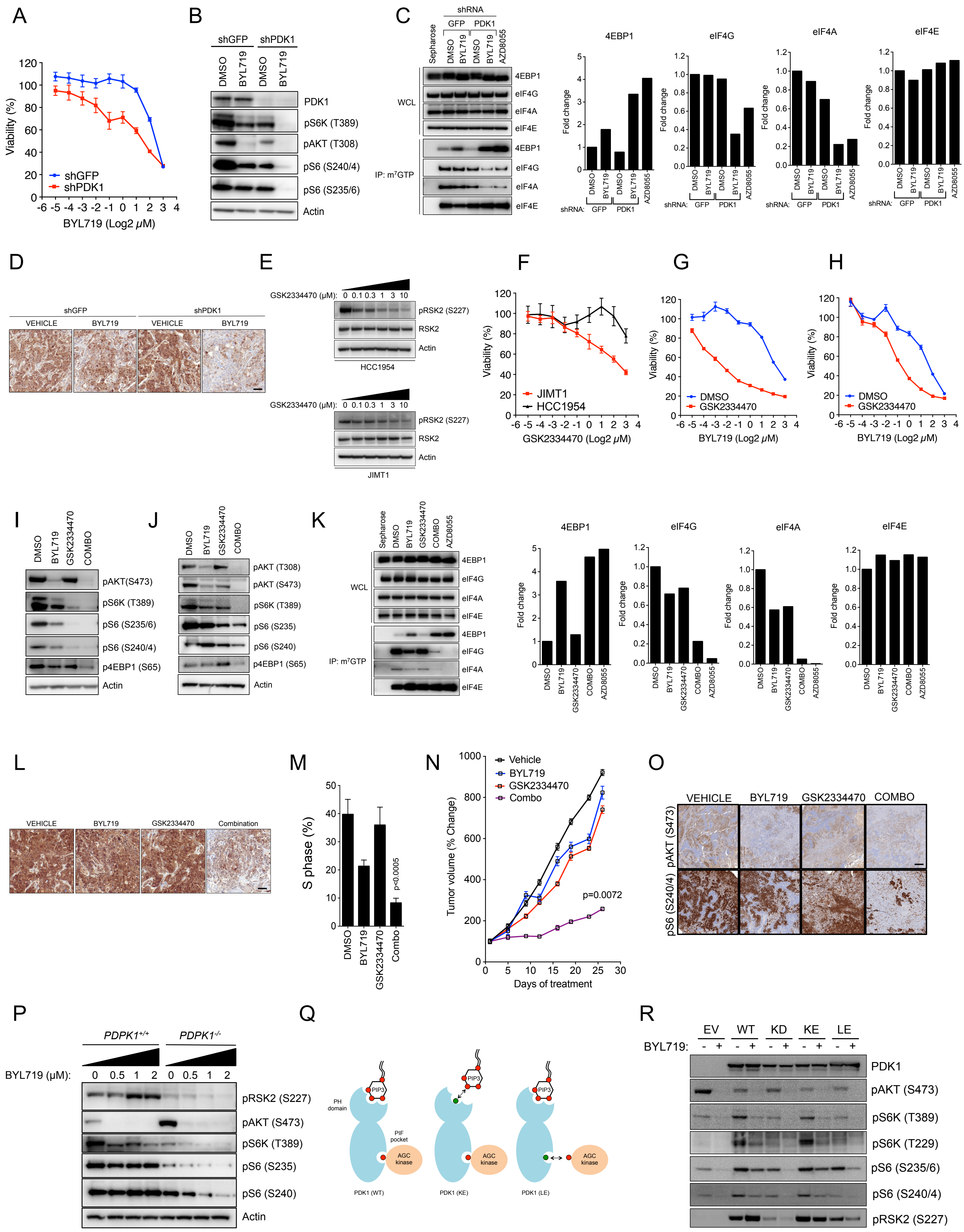

A
B
C
D
E
F
G
H
I
J
K
L
M
N
O
P
Q
R
shGFP
shPDK1
BYL719 (Log2 μM)
Viability (%)
PDK1
pS6K (T389)
pAKT (T308)
pS6 (S240/4)
pS6 (S235/6)
Actin
shRNA
Sepharose
WCL
IP: m7GTP
4EBP1
eIF4G
eIF4A
eIF4E
Fold change
VEHICLE
BYL719
GSK2334470 (μM):
pRSK2 (S227)
RSK2
HCC1954
JIMT1
GSK2334470 (Log2 μM)
DMSO
GSK2334470
COMBO
AZD8055
pAKT(S473)
p4EBP1 (S65)
Combination
S phase (%)
p<0.0005
Tumor volume (% Change)
Days of treatment
Vehicle
Combo
p=0.0072
pAKT (S473)
pS6 (S240/4)
PDPK1+/+
PDPK1-/-
BYL719 (μM):
PH domain
PIF pocket
AGC kinase
PIP3
PDK1 (WT)
PDK1 (KE)
PDK1 (LE)
EV
WT
KD
KE
LE
BYL719:
pS6K (T229)

Figure S2. Relative to Figure 1

(A) Western blot comparing JIMT1 shGFP and shPDK1 cells treated with BYL719 (1 μM) for 4 hr. **(B)** Dose-response curves from JIMT1 shGFP and shPDK1 cells treated with BYL719 for 6 days. **(C)** m^7GTP pull down assay for HCC1954 shGFP and shPDK1 cells treated with BYL719 (1 μM) for 4 hr. Quantification of the m^7GTP-precipitated proteins is indicated in fold change. AZD8055 is used as a control at 1 μM. **(D)** p4EBP1 (T37/46) IHC from the tumors harvested from Figure 1E. Scale bar: 100 μM. **(E)** Western blot analysis of phosphorylated RSK2 (S227) in HCC1954 and JIMT1 cells treated with increasing concentrations of GSK2334470 for 8 hr. **(F)** Dose-response curves from HCC1954 and JIMT1 resistant cell lines treated with GSK2334470 for 6 days. **(G)** Dose-response curves from JIMT1 cells treated with BYL719 in the presence or absence of GSK2334470 (1 μM) during 6 days. **(H)** Same as (G) using BT20 TNBC cells. **(I)** Western blot comparing JIMT1 cells treated with BYL719 (1 μM), GSK2334470 (1 μM), or the combination of both agents for 4 hr. **(J)** Same as (I) using BT20 TNBC cells. **(K)** m^7GTP pull down assay for HCC1954 cells treated with BYL719 (1 μM), GSK2334470 (1 μM), or the combination of both agents for 4 hr. Quantification of the m^7GTP-precipitated proteins is indicated in fold change. AZD8055 is used as a control at 1 μM. **(L)** p4EBP1 (T37/46) IHC from the tumors harvested from Figure 1K. Scale bar: 100 μM. **(M)** S-phase quantification in JIMT1 cells treated with BYL719 (1 μM), GSK2334470 (1 μM), or the combination of both agents for 24 hr and stained with Propidium iodide for cell cycle analysis. **(N)** JIMT1 in vivo xenograft treated with Vehicle, BYL719, GSK2334470, or the combination of both agents (n=10/arm). **(O)** IHC analysis of tumors from (N) collected at the end of the experiment 4 hr after the last dosage. Scale bar: 100 μM. **(P)** Western blot analysis of HCT116 *PDPK1*+/+ and *PDPK1*-/- isogenic cell lines treated with increasing concentrations of BYL719 for 4 hr. **(Q)** Schematic representation of the effects of the PIP3-binding and PIF-binding pocket deficient mutants used in (R). Red circles indicate phosphate groups and green circles indicate hydrophilic-charged aminoacid E. Arrows indicate electric charge repulsion. **(R)** Western blot of HCT116 *PDPK1*-/- cells transfected with different pCCL-PDK1 mutants. EV (empty vector), WT (wild type), KD (kinase death; K111N), KE (PIP3-binding deficient; K465E), LE (PIF-binding pocket deficient; L155E). Cells were treated with BYL719 (1 μM) for 4 hr before collection.

p values are calculated using Student's t-test. Error bars are ± SEM.

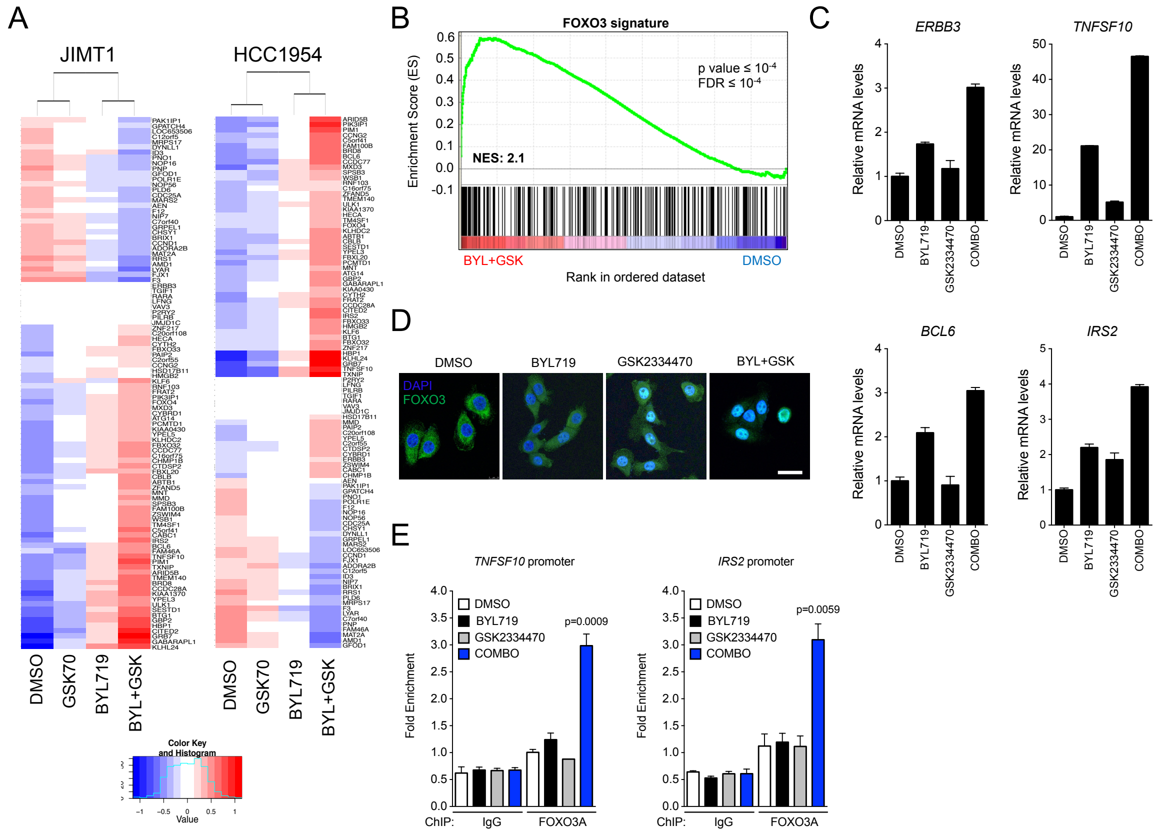

Figure S3. Relative to Figure 2

(A) Differentially expressed genes in JIMT1 (left) and HCC1954 (right) cells treated with BYL719 (1 μM), GSK2334470 (1 μM), or the combination of both agents for 4 hr. Gene expression up-regulation is indicated in red, while blue represents gene expression down-regulation. **(B)** Enrichment plot for the FOXO3 signature of GSEA in JIMT1 cells. NES: Normalized Enrichment Score. **(C)** *ERBB3*, *TNFSF10*, *BCL6*, and *IRS2* mRNA expression in JIMT1 cells treated with DMSO, BYL719 (1 μM), GSK2334470 (1 μM), or the combination of both agents for 4 hr. **(D)** FOXO3A immunofluorescence (green) in JIMT1 cells treated with DMSO, BYL719 (1 μM), GSK2334470 (1 μM), or the combination of both agents for 4 hr. Nuclei are shown in blue (DAPI). Scale bar: 25 μM. **(E)** ChIP-qPCR assay of FOXO3A binding at the *TNFSF10A* and *IRS2* promoters in JIMT1 cells treated as indicated in (C).

p values are calculated using Student's t-test. Error bars are ± SEM.

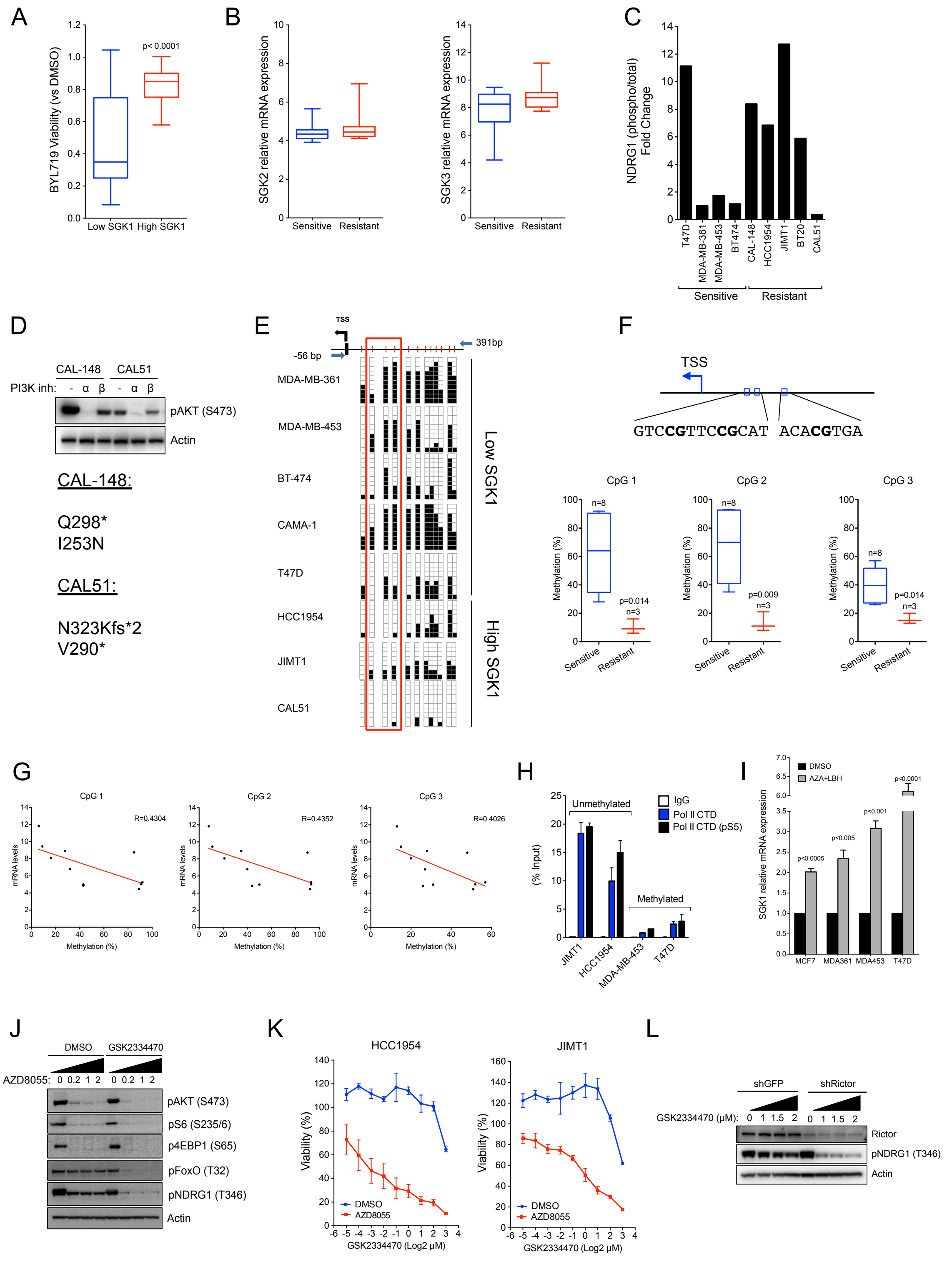
A
BYL719 Viability (vs DMSO)
p< 0.0001
Low SGK1
High SGK1
B
SGK2 relative mRNA expression
SGK3 relative mRNA expression
Sensitive
Resistant
C
NDRG1 (phospho/total) Fold Change
T47D
MDA-MB-361
MDA-MB-453
BT474
CAL-148
HCC1954
JIMT1
BT20
CAL51
Sensitive
Resistant
D
CAL-148
CAL51
PI3K inh:
- α β - α β
pAKT (S473)
Actin
CAL-148:
Q298*
I253N
CAL51:
N323Kfs*2
V290*
E
TSS
-56 bp
391bp
MDA-MB-361
MDA-MB-453
BT-474
CAMA-1
T47D
HCC1954
JIMT1
CAL51
Low SGK1
High SGK1
F
TSS
GTCCGTTCCGCAT ACACGTGA
CpG 1
CpG 2
CpG 3
Methylation (%)
n=8
n=3
p=0.014
p=0.009
p=0.014
Sensitive
Resistant
G
CpG 1
CpG 2
CpG 3
R=0.4304
R=0.4352
R=0.4026
mRNA levels
Methylation (%)
H
Unmethylated
Methylated
IgG
Pol II CTD
Pol II CTD (pS5)
(% Input)
JIMT1
HCC1954
MDA-MB-453
T47D
I
DMSO
AZA+LBH
SGK1 relative mRNA expression
p<0.0005
p<0.005
p<0.001
p<0.0001
MCF7
MDA361
MDA453
T47D
J
DMSO
GSK2334470
AZD8055:
0 0.2 1 2 0 0.2 1 2
pAKT (S473)
pS6 (S235/6)
p4EBP1 (S65)
pFoxO (T32)
pNDRG1 (T346)
Actin
K
HCC1954
JIMT1
Viability (%)
DMSO
AZD8055
GSK2334470 (Log2 μM)
L
shGFP
shRictor
GSK2334470 (μM):
0 1 1.5 2 0 1 1.5 2
Rictor
pNDRG1 (T346)
Actin

Figure S4. Relative to Figure 3

(A) Cell viability of breast cancer cell lines treated with BYL719 (2 μM) and classified according to the SGK1 mRNA expression in high (>median expression) and low (<median expression) (n=27). Box indicates the median and the interquartile range, while whiskers represent minimum and maximum. **(B)** SGK2 and SGK3 mRNA levels in breast cancer cell lines sensitive or resistant to BYL719 (n=27). Box indicates the median and the interquartile range, while whiskers represent minimum and maximum. **(C)** Quantification of pNDRG1 (T346) basal levels in *PIK3CA*-mutant breast cancer cell lines classified according to their sensitivity to BYL719. **(D)** pAKT (S473) Western blot in CAL-148 and CAL51 cells treated with BYL719 (1 μM) and AZD6482 (1 μM) during 4 hr. Mutations identified in *PTEN* are shown below. **(E)** Bisulfite sequencing of the promoter region of *SGK1* in a cohort of eight breast cancer cell lines classified according to their sensitivity to BYL719. In red, the three CpG sites identified to be differentially methylated. TSS: Transcription Start Site. **(F)** Schematic representation of the three CpG sites (bold) identified to be differentially methylated in the promoter of *SGK1*. Below, pyrosequencing quantification of the methylated CpG sites in eleven breast cancer cell lines classified according to their sensitivity to BYL719. Box indicates the median and the interquartile range, while whiskers represent minimum and maximum. **(G)** Correlation between the SGK1 mRNA levels and the percentage of CpG promoter methylation in the cells indicated in (F). R indicates the R-square goodness of fit, and all correlations had a significant p value <0.05. **(H)** ChIP-qPCR assay of RNA Polymerase II (Pol II) and the phosphorylated S5 of RNA Polymerase II (Pol II pS5) for *SGK1* promoter in unmethylated (resistant) and methylated (sensitive) cell lines. Primers for the *SGK1* promoter were design in order to amplify the region containing the three CpG islands identified in this study. CTD: C-terminal domain. **(I)** RT-qPCR analysis of SGK1 mRNA levels in methylated sensitive cell lines treated for 72 hr with the demethylating agent 5-aza-2'-deoxycytidine(5 μM) and the histone deacetylase inhibitor panobinostat (LBH) (50 nM). **(J)** Western blot analysis of HCC1954 cells treated with increasing concentrations of the mTOR catalytic inhibitor AZD8055 (μM) in the presence or absence of GSK2334470 (1 μM) for 4 hr. **(K)** Dose-response curves of HCC1954 and JIMT1 cell lines treated with increasing concentrations of GSK2334470 in the presence or absence of the mTOR catalytic inhibitor AZD8055 (1 μM) for 6 days. **(L)** Western blot analysis of JIMT1 cells stably expressing shRNA against GFP and RICTOR mRNA and treated with increasing concentrations of GSK2334470.

p values are calculated using Student's t-test. Error bars are ± SEM.

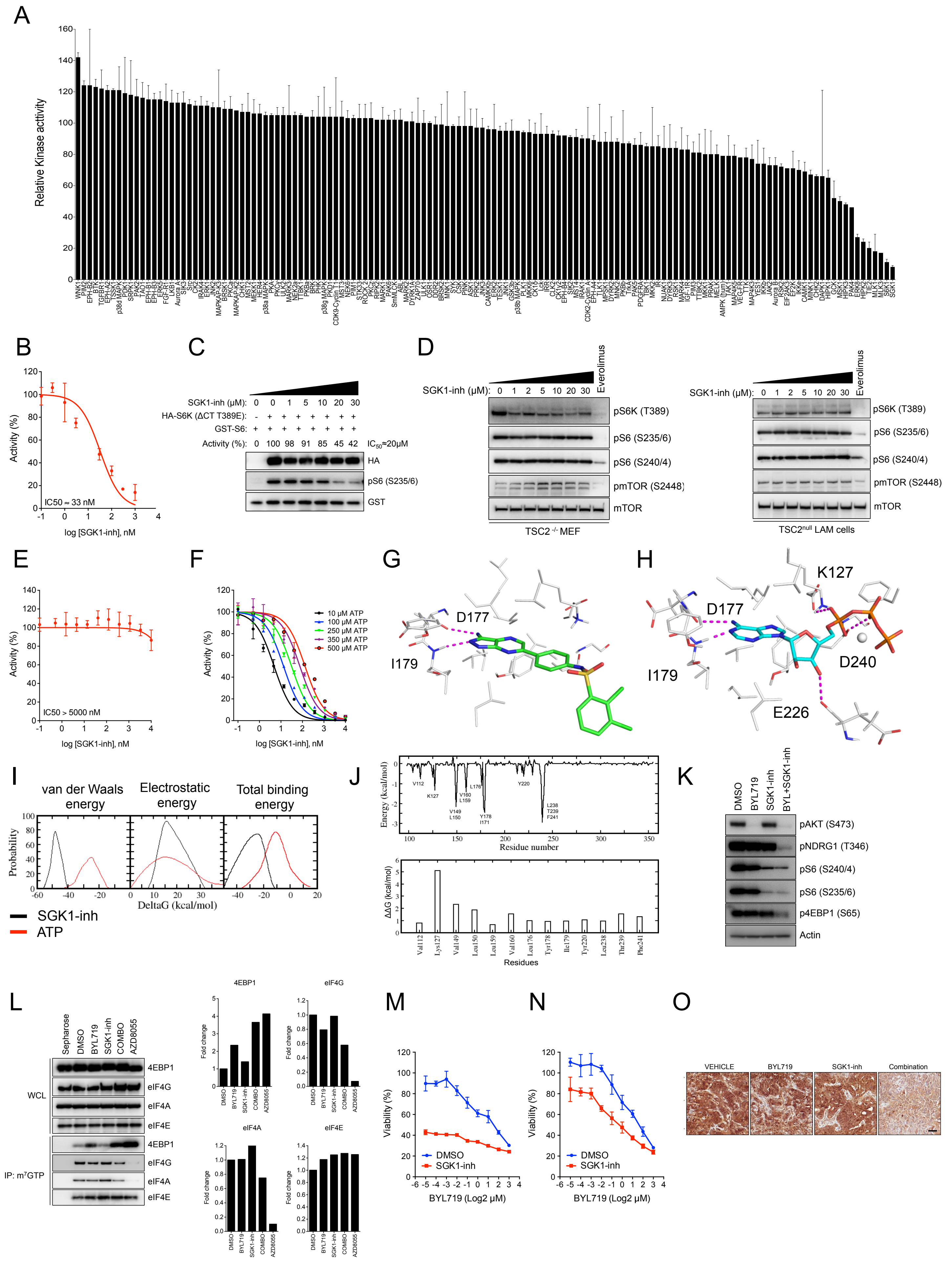

A
Relative Kinase activity
B
Activity (%)
IC50 ≈ 33 nM
log [SGK1-inh], nM
C
SGK1-inh (μM): 0 0 1 5 10 20 30
HA-S6K (ΔCT T389E): - + + + + + +
GST-S6: + + + + + + +
Activity (%): 0 100 98 91 85 45 42
IC50≈20μM
HA
pS6 (S235/6)
GST
D
SGK1-inh (μM): 0 1 2 5 10 20 30
Everolimus
pS6K (T389)
pS6 (S235/6)
pS6 (S240/4)
pmTOR (S2448)
mTOR
TSC2 -/- MEF
TSC2null LAM cells
E
Activity (%)
IC50 > 5000 nM
log [SGK1-inh], nM
F
10 μM ATP
100 μM ATP
250 μM ATP
350 μM ATP
500 μM ATP
Activity (%)
log [SGK1-inh], nM
G
D177
I179
H
K127
D177
I179
D240
E226
I
van der Waals energy
Electrostatic energy
Total binding energy
Probability
DeltaG (kcal/mol)
SGK1-inh
ATP
J
Energy (kcal/mol)
V112
K127
V149
L150
V160
L159
L176
Y178
I171
Y220
L238
T239
F241
Residue number
ΔΔG (kcal/mol)
Val112
Lys127
Val149
Leu150
Leu159
Val160
Leu176
Tyr178
Ile179
Tyr220
Leu238
Thr239
Phe241
Residues
K
DMSO
BYL719
SGK1-inh
BYL+SGK1-inh
pAKT (S473)
pNDRG1 (T346)
pS6 (S240/4)
pS6 (S235/6)
p4EBP1 (S65)
Actin
L
Sepharose
DMSO
BYL719
SGK1-inh
COMBO
AZD8055
WCL
IP: m7GTP
4EBP1
eIF4G
eIF4A
eIF4E
Fold change
M
Viability (%)
DMSO
SGK1-inh
BYL719 (Log2 μM)
N
Viability (%)
DMSO
SGK1-inh
BYL719 (Log2 μM)
O
VEHICLE
BYL719
SGK1-inh
Combination

Figure S5. Relative to Figure 4

(A) Selectivity screening results of SGK1-inh at 1 μM against a library containing 140 kinases representative of the human kinome. Values are available at the Kinase Inhibitor Database of the MRC Protein Phosphorylation and Ubiquitylation Unit of the University of Dundee (http://www.kinasescreen.mrc.ac.uk/kinase-inhibitors). **(B)** In vitro S6K1 kinase assay using recombinant KKRNRTLTK peptide as a substrate in the presence of increasing concentrations of SGK1-inh. IC_{50} value is indicated. **(C)** In vitro S6K1 kinase assay using constitutively active S6K kinase immunoprecipitated from 293T cells expressing HA-S6K (ΔCT T389E) and treated with increasing concentrations of SGK1-inh. Recombinant GST-S6 was used as a substrate and phosphorylated S6 (S235/6) antibody was used for the detection of phosphorylated substrate by Western blot. IC_{50} value is indicated. **(D)** Western blot analysis of S6K targets in *TSC2* knockout mouse embryonic fibroblasts (MEF) and fibroblasts derived from a *TSC2*^{null} Lymphangioleiomyomatosis (LAM) patient treated with increasing concentrations of SGK1-inh for 4 hr. Everolimus was used as a positive control at 200 nM. **(E)** In vitro mTOR kinase assay using recombinant 4EBP1 as a substrate in the presence of increasing concentrations of SGK1-inh. IC_{50} value is indicated. **(F)** SGK1-inh IC_{50} determination in an ATP competition assay using increasing concentration of ATP. **(G)** Residues involved in the interaction between SGK1 (active conformation) and SGK1-inh. Hydrogen bonds are shown as purple dotted lines. **(H)** Residues involved in the interaction between ATP and the active conformation of SGK1. Hydrogen bonds are shown as purple dotted lines and Mg^{+2} as a grey sphere. **(I)** Distribution of free energies (ΔG) of the conformations sampled during MD simulations of SGK1 bound to SGK1-inh (black) or ATP (red). Distribution of van der Waals' interactions and electrostatic solvation contribution for the total binding energy are shown. **(J)** Upper panel. Decomposition of binding free energy on per-residue basis for SGK1 (DFG-out conformation) and SGK1-inh complex. Lower panel. Alanine scanning results for the selected residues are shown. Results are expressed in the change of free energy when the indicated residues are mutated to alanine ΔΔG. **(K)** Western blot analysis of JIMT1 cells treated with BYL719 (1 μM), SGK1-inh (10 μM), or the combination of both agents for 4 hr. **(L)** m^7GTP pull down assay for HCC1954 cells treated with BYL719 (1 μM), SGK1-inh (10 μM), or the combination of both agents for 4 hr. Quantification of the m^7GTP-precipitated proteins is indicated in fold change. AZD8055 is used as a control at 1 μM. **(M)** Dose-response curves from JIMT1 cells treated with increasing concentrations of BYL719 in the presence or absence of SGK1-inh (2 μM) for 6 days. **(N)** Dose response curves from JIMT1 cells treated with increasing concentrations of BYL719 in the presence or absence of SGK1-inh (2 μM) for 6 days. **(O)** p4EBP1 (T37/46) IHC from the tumors harvested from Figure 4L. Scale bar: 100 μM.

Error bars are ± SEM.

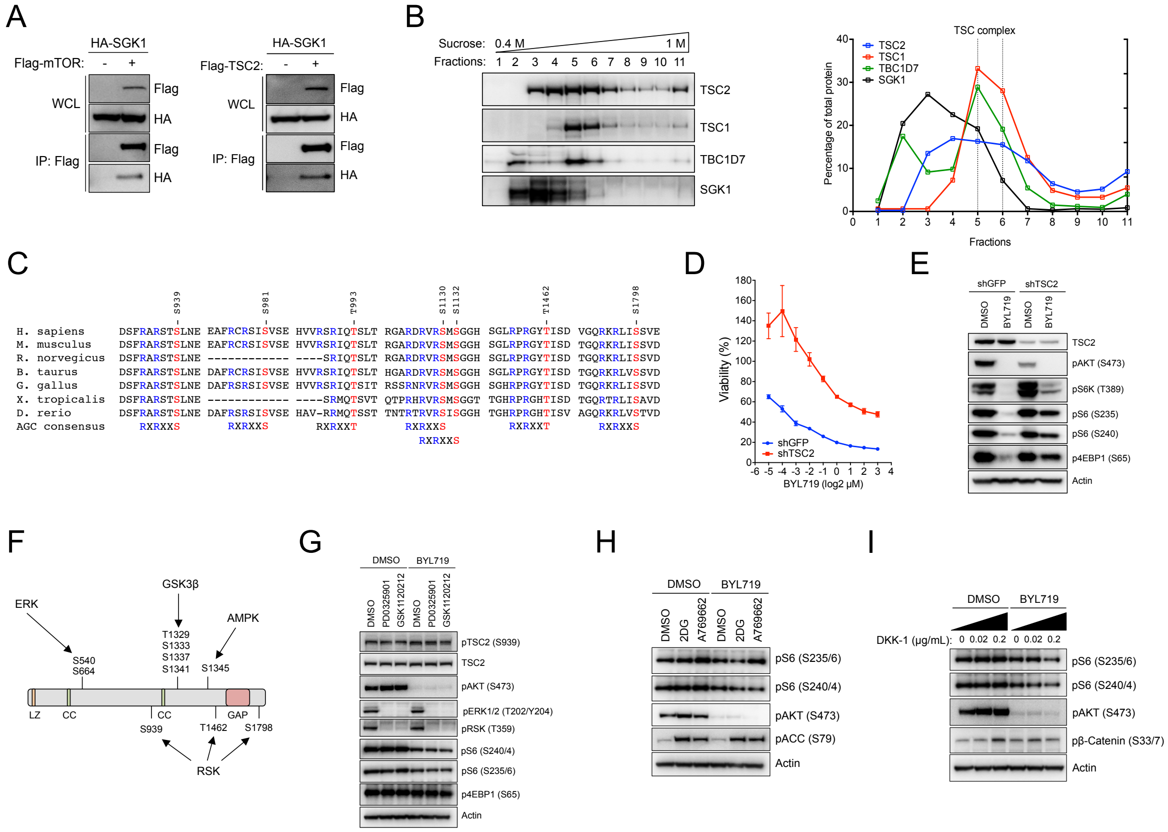

Figure S6. Relative to Figure 5

(A) Co-immunoprecipitation assay using Flag-mTOR (left) or Flag-TSC2 (right) and HA-SGK1 in 293T cells. **(B)** Western blot analysis of sucrose gradient fractions collected upon ultracentrifugation. Columns were packed in densities ranging from 0.4 to 1 M of sucrose, and a small aliquot of each fraction was analyzed by Western blot. Densitometry quantification of the sucrose gradient results are represented and dotted lines indicate the fractions in which the TSC complex is highly enriched, as assessed by the immunodetection of the three components TSC1, TSC2, and TBC1D7. **(C)** Alignment of the sequence of TSC2 comprising the AGC phosphorylation motifs RXRXX(S/T). R-1 and R-3 are highlighted in blue and phosphorylatable S or T in red. Alignment was performed with ClustalW2 using the protein sequence from mouse (*Mus musculus*), rat (*Rattus norvegicus*), cattle (*Bos taurus*), chicken (*Gallus gallus domesticus*), frog (*Xenopus tropicalis*), and zebrafish (*Danio rerio*). **(D)** Dose-response curves from T47D cells transduced with lentivirus expressing shGFP and shTSC2 and treated with increasing concentrations of BYL719 for 6 days. **(E)** Western blot from T47D shGFP and shTSC2 cells treated with BYL719 (1 μM) for 4 hr. **(F)** Representative signaling integration of other kinases involved in the phosphorylation of TSC2. Residues previously identified to be phosphorylated by the indicated kinases are shown. Domains are indicated: LZ (leucine zipper); CC (coiled coil); GAP (GTP-ase activation protein). **(G)** Western blot analysis of HCC1954 cells treated with the MEK inhibitors PD0325901 (1 μM) and GSK1120212 (50 nM) in the presence or absence of BYL719 (1 μM) for 4 hr. **(H)** Western blot analysis of HCC1954 cells treated with the AMPK inductors 2-deoxyglucose (50 mM) and A769662 (300 μM) in the presence or absence of BYL719 (1 μM) for 4 hr. Phosphorylation of the previously described substrate Acetyl-CoA Carboxylase (ACC) S79 is shown as control for AMPK activation. **(I)** Western blot analysis of HCC1954 cells treated with increasing concentrations of the WNT antagonist Dickkopf WNT signaling pathway inhibitor 1 (DKK-1) for 30 min in the presence or absence of BYL719 (1 μM) for 4 hr. Phosphorylation of the previously described substrate β-catenin S33/7 is shown as control for GSK3β activation.

Error bars are ± SEM.

Supplemental Experimental Procedures

RNAi screening

The synthetic lethal RNAi screening was carried out at the High-Throughput Screening Core Facility of MSKCC. The kinome and phosphatome Ambion Silencer Select v4.0 libraries were purchased from Life Technologies and contain 2130 unique siRNAs targeting each of the 710 human kinase genes and 894 unique siRNAs targeting each of the 298 human phosphatase genes. Diluted siRNA were transferred into assay plates at a final concentration of 50 nM. As a reference, we used Silencer Select Negative Control #1 siRNA (4390843) as a negative control and PLK1 siRNA (s449) as the positive control.
JIMT1 and HCC1954 cells were seeded and were reverse transfected using Dharmafect-1 at 0.05 μL/well. Next, cells were treated with DMSO or BYL719 1 μM and 7 days after transfection, cell viability was assessed using Alamar blue and Nuclei Count using Hoechst staining and quantified using LEADseeker (GE Healthcare) and INCA2000 (GE Healthcare), respectively.
For the hit nomination, the BDA method was used as previously described (Bhinder and Djaballah, 2012). Briefly, this method comprises 5 steps to analyze and score active siRNA duplexes and genes: (1) active duplex identification, (2) active gene identification, (3) off-target effects filtering, (4) re-scoring, and (5) biological classifications. To identify modulators of BYL719 resistance, active genes were nominated from the active siRNA duplexes using a hit rate per gene (H score) of ≥60. H score is defined as follows:

$$\text{H score} = \frac{\text{number of active siRNA duplex}}{\text{total number of siRNA duplexes}} \times 100$$

Using this approach, 5 genes were identified and the two most active duplexes of each gene were purchased and screened for cell viability and pS6 staining in the presence of BYL719 1 μM. siRNA were from Ambion: *PIK3CA* (s10520, s10522), *MTOR* (s602, s603), *PDPK1* (s10274, s10275), *PAPL* (s52890, s52892), and *PP1R12A* (s935, s937). Confirmation screening was carried out as described above. For pS6 (S240/4) staining, cells were reverse-transfected and after 72 hr, they were treated for 4 hr with BYL719 at 1 μM. Next, cells were fixed with 4% Paraformaldehyde in PBS and stained using pS6 (S240/4) antibody from Cell Signaling (2215), followed by Alexa Fluor 488 secondary antibody. Fluorescence was quantified using INCA2000 (GE Healthcare).
Final nomination was performed using the H score described above and genes that sensitized cells to BYL719 and decreased pS6 (S240/4) were selected.

Plasmids and site-directed mutagenesis

The Myc-tagged constructs pCCL-PDK1 WT, KD (K111N), K465E, and L155E were a gift from Dr. Primo and Dr. Gagliardi (University of Turin). pLPCX-HA-SGK1(Δ60) was obtained from Dr. Conzen (The University of Chicago) and was used as a template to subclone the cDNA and generate pdEYFP-SGK1(Δ60) and pLenti7.3-V5-SGK1(Δ60,S422D). The kinase-inactive K127A and constitutively active S422D mutant were generated using PCR-based site-directed mutagenesis.
Plasmids expressing Flag-tagged mTOR (26603), TSC1 (8995), TSC2 (8996), RHEB (15888), and HA-S6K (ΔCT, T389E) (8993) were obtained from Addgene. pcDNA3-Flag-TSC2 WT and 5A (S939A, S981A, S1130A, S1132A, T1462A) were a gift from Dr. Manning and were used as a template for the generation of pcDNA3-Flag-TSC2 6A (5A, S1798A) and pcDNA3-Flag-TSC2 6E, respectively. Plasmids encoding for the TSC2 truncation mutants were provided by Dr. Xiong (University of North Carolina at Chapel Hill) and pEGFP-TSC2 was from Dr. Krymskaya (University of Pennsylvania). *PDPK1* targeting shRNA pLKO-based vector used in this study was TRCN0000039782, although other clones were also tested. *RICTOR*-targeting shRNA plasmid was from Addgene (1853).
Lentiviral doxycycline-inducible mirE-embedded shRNAs were XhoI/EcoRI cloned into the LT3GEPIR vector previously described (Fellmann et al., 2013). Briefly, this all-in-one vector contains the puromycin resistance and the reverse transactivator (rtTA3) under the control of the constitutive phosphoglycerate kinase (PGK) promoter. The shRNA and the fluorescent marker GFP are expressed under the control of the Tet-responsive element promoter (T3G). Control shRNA was a hairpin designed against the *Renilla reniformis* luciferase. *SGK1* shRNA was chosen experimentally based on five different hairpins. The sequence targeting the exon 5 provided the most robust results.

REN shRNA:

5’-TGCTGTTGACAGTGAGCGCAGGAATTATAATGCTTATCTATAGTGAAGCCACAGATGTATAGATAA GCATTATAATTCCTATGCCTACTGCCTCGGA-3’

SGK1#282 shRNA:

5’-TGCTGTTGACAGTGAGCGCAGAAGTGTTCTATGCAGTCAATAGTGAAGCCACAGATGTATTGACTG CATAGAACACTTCTTTGCCTACTGCCTCGGA-3’

All constructs were validated by Sanger sequencing.

Cells and lentiviral production

All cell lines were obtained from ATCC except for JIMT1 (AddexBio), used at low passages, and maintained at 37°C in a 5% CO_2 atmosphere in the recommended culture media. HCT116 *PDPK1*$^{-/-}$ and *PDPK1*$^{+/+}$ cells were a gift from Dr. Mills (MD Anderson) and were originally generated by Dr. Vogelstein’s laboratory (Johns Hopkins University) (Ericson et al., 2010).
For lentiviral production, 293T cells were seeded in 10-cm plates, transfected with pCMV-VSVG, pCMV-dR8.2, and the plasmid of interest using FuGene HD (Promega). Viruses were collected 72 hr post-transfection, filtered through a 0.45 µm filter (Millipore), and recipient cells were infected twice using viral supernatants supplemented with 8 µg/µL of polybrene (Sigma). Transduced cells were selected using puromycin (2 µg/mL) or Fluorescence Activated Cell Sorting (FACS) for the pCCL and pLenti7.3 vectors, which contain EGFP as a selectable marker.

Reagents, cell viability and apoptosis

BYL719 and MK2206 were obtained from the Stand Up to Cancer (SU2C) pharmacy. GSK2334470 and Staurosporine were purchased at Selleckchem. SGK1-inh was a gift from M. Nazare and N. Halland. All drugs were dissolved in DMSO for in vitro experiments.
Cell viability was measures using the MTT assay. Briefly, 5000 cells were seeded in 96 well plates, treated for 6 days, and assayed using 0.25% MTT (Sigma) and 50 mM sodium succinate (Sigma) solutions for 3 hr. Formazan crystals were dissolved with DMSO and absorbance was measured at 570 nm of wavelength.
For Caspase 3/7 activity, the Caspase-Glo® 3/7 Assay kit from Promega was used following manufacturer’s instructions. The caspase inhibitor zVAD-fmk was used to inhibit apoptosis in cells and was also obtained from Promega.

Immunoblot, immunoprecipitation, and kinase assay

For western blot analysis, proteins were extracted in RIPA buffer supplemented with protease and phosphatase inhibitors (Roche). Protein lysates were separated using SDS-PAGE gels and transferred to a PVDF membrane. Then, membranes were probed using specific antibodies. PDK1, pAKT (S473), pAKT (T308), pS6K (T389), pS6 (S240/4), pS6 (235/6), p4EBP1 (S65), PARP, Actin, pRSK (S227), cleaved Caspase 3, pFOXO1/3 (T24/T32), SGK1, SGK2, SGK3, pNDRG1 (T346), NDRG1, Flag, HA, TSC2, pAcetyl-CoA Carboxylase (Ser79), pbetaCatenin (S33/7), pERK1/2 (T202/Y203), pRSK (T359), pTSC2 (S939), TBDC1D7, TSC1, and phospho-RXRXX(S/T) were from Cell Signaling Technology (CST). For S6K T229 phosphorylation detection we used pPKC (pan) (γT514) (9379) from CST, as previously reported (Garcia-Martinez and Alessi, 2008). The SGK1 and AKT antibodies for endogenous immunoprecipitation were raised in sheep by the Division of Signal Transduction Therapy (DSTT) at the University of Dundee and affinity-purified against the indicated antigens: anti-AKT1 (S695B, third bleed; raised against residues 466–480 of human Akt1: RPHFPQFSYSASGTA), anti-SGK1 antibody (S062D, third bleed, raised against recombinant SGK1 protein (DU35257). For endogenous co-immunoprecipitation of SGK1 and TSC2, we employed the S062D sheep antibody in 10 mg of JIMT1 lysate and TSC2 was recognized using the CST rabbit antibody with a secondary conformational specific antibody (Clean Blot from Thermo).
For immunoprecipitation assays, 293T cells were transiently transfected with appropriate plasmids and 24 hr post-transfection, cells were washed in cold PBS and lysed using NP-40 buffer (150 mM NaCl, 10 mM Tris pH 8, 1% NP-40, 10% glycerol). Lysates were rotated at 4°C for 4 hr with EZview™ Red ANTI-FLAG® M2 or ANTI-HA agarose beads (Sigma) and washed three times using NP-40 buffer. For in vitro kinase assay, immunoprecipitated

Flag-TSC2 was used as a substrate in a reaction with recombinant His-SGK1 (Δ60) (MRC-PPU Reagents) and ATP (Signalchem) in kinase assay buffer containing 25 mM MOPS pH 7.2, 12.5 mM β-glycerophosphate, 25 mM $MgCl_2$, 5 mM EGTA, 2 mM EDTA and 0.25 mM DTT at 30°C for 30 minutes. In vitro kinase activity of endogenous SGK1 and AKT was assayed by measuring [γ-^{32}P] ATP incorporation into Crosstide substrate peptide [GRPRTSSFAEGKK]. SGK1 and AKT were immunoprecipitated from HCC1954 cell line 4 hr after treatment. Immunoprecipitates were washed once with lysis buffer containing 500 mM NaCl, once with lysis buffer, and twice with Buffer A (50 mM Tris pH 7.5, 0.1 mM EGTA). Reactions were carried out in 40 μL total volume containing 0.1 mM [γ-^{32}P] ATP (400-1000 cpm/pmol), 10 mM magnesium acetate, and 30 μM Crosstide peptide. Reactions were terminated by adding 10 μL 0.1 mM EDTA. 40 μL of the reaction mix was spotted on P81 paper, which was immediately immersed into 50 mM ortophosphoric acid and washed several times. Papers were rinsed in acetone and air dried. Radioactivity was quantified by Cerenkov counting. One unit of enzyme activity was defined as the amount of enzyme that catalyzes incorporation of 1 nmol of [γ-^{32}P] ATP into the substrate over one minute.

m^7GTP pull downs

2 million cells were seeded in 10 cm plates and treated accordingly 12 hr after seeding. Lysates were prepared using m^7GTP pull down buffer (50 mM Hepes, pH 7.4, 75 mM NaCl, 10 mM MgCl2, 1 mM DTT, 8 mM EGTA, 10 mM β-glycerophosphate, 0.5 mM Na_3VO_4, 0.5% Triton X-100) supplemented with protease and phosphatase inhibitors. Lysates were centrifuged at 13000 rpm for 10 min and supernatants were rotated for 2 h at 4°C with 7-methyl-GTP-Sepharose or control Sepharose beads (Jena Bioscience). Beads were washed three times with m^7GTP pull down buffer, resuspended in Laemmli buffer, and associated proteins were detected by Western blot.

Mass spectrometry

Kinase assay reactions were performed in biological triplicates and resolved using SDS-polyacrylamide gel electrophoresis, stained with SimplyBlue SafeStain (Life Technologies, Thermo Fisher Scientific), and the band corresponding to Flag-TSC2 was excised and digested with trypsin as described by (Shevchenko et al., 2006). The tryptic peptides were resuspended in buffer A containing 3% formic acid and analyzed by microcapillary liquid chromatography with tandem mass spectrometry using a NanoAcquity LC (Waters) with a 100 μm-inner-diameter x 10 cm-length C18 column (1.7μm BEH130, Waters) configured with a 180 μm x 2 cm trap column coupled to a Q-Exactive mass spectrometer (Thermo Fisher Scientific) scanning 380-1800 m/z at 70,000 resolution with AGC set at 3×10^6. Peptides were eluted with a linear gradient of 2-30% acetonitrile (0.1% formic acid) in water over 90 min at a flow rate of 300 nL/min. Key parameters for the data dependent MS were top 10 DDA, AGC 5e4, and ms/ms resolution of 17,000. Data were analyzed using MaxQuant (Max Planck Institute of Biochemistry, Germany; version 1.5.1.0) at default settings with a few modifications. The default was used for first search tolerance and main search tolerance: 20 ppm and 6 ppm, respectively. MaxQuant was set up to search the reference human proteome database. Maxquant performed the search using trypsin digestion with up to 2 missed cleavages. Peptide, Site and Protein FDR were all set to 1% with a minimum of 1 peptide needed for identification but 2 peptides needed to calculate a protein ratio. LFQ quantitation was confirmed by manual integration of the MS1 data for the phosphorylation sites of interest. Raw data as well as original MaxQuant results files can be provided upon request.

Microarray, qPCR, and ChIP-qPCR

RNA was isolated from cells using the QIAGEN RNeasy kit. For microarray analysis, biotinylated cRNA was prepared according to the standard Illumina protocol. After fragmentation, cRNA was hybridized with Illumina GX HT12 Human Array. Slides were washed and stained in the Illumina instrument following manufacturer's protocol. Slides were scanned using Illumina Bead Array Reader. Data were analyzed using GenomeStudio software. No normalization and background correction are performed first, then quantile normalization and background correction are done.

For mRNA expression analysis, cDNA was prepared using the Bio-Rad cDNA synthesis kit. cDNA was amplified by quantitative PCR using SYBR Select Master Mix (Applied Biosystems) in the ViiA 7 Real-Time PCR system. All reactions were carried out in triplicate. Primers used for mRNA expression were:

ERBB3: Fw-CTGATCACCGGCCTCAAT; Rv-GGAAGACATTGAGCTTCTCTGG
IRS2: Fw-TTCTTGTCCCACCACTTGAA; Rv-CTGACATGTGACATCCTGGTG
TNFSF10: Fw-CCTCAGAGAGTAGCAGCTCACA; Rv-CAGAGCCTTTTCATTCTTGGA

BCL6: Fw-CTGCAGATGGAGCATGTTGT; Rv-TCTTCACGAGGAGGCTTGAT
Actin: Fw-CGTCTTCCCCTCCATCGT; Rv-GAAGGTGTGGTGCCAGATTT

ChIP assays were performed as described previously (Toska et al., 2012). Briefly, cells were treated with 1% formaldehyde for 15 min at room temperature and quenched with ice-cold 125 nM glycine for 5 min. Lysed cells were sonicated on ice to yield 200-800 bp DNA fragments. Chromatin was incubated overnight at 4°C with 2 µg of anti-FOXO3A antibody (Santa Cruz Biotechnology; sc-11351), anti-RNA polymerase II CTD (YSPTSPS) antibody (Abcam; ab817), anti-RNA polymerase II CTD (phospho S5) (Abcam; ab5131) or nonspecific IgG. Immunocomplexes were precipitated by incubation overnight with protein G-conjugated beads. Immunoprecipitates were washed and crosslinks were reversed by heating to 65°C for 6 hr and then treated with proteinase K for 1 hr at 55°C. Chromatin was purified using QiaQuick PCR clean-up columns. ChIP primers used in this study were:
Control: Fw-CAGCTCAGTGCTGTTGGTGG; Rv-ACCATCCAACCCTGGAGATC
IRS2 promoter: Fw-GACAATCAAAGTCCTTCCCAAA; Rv-CCTTTTGACCTGTGCTGTTGT
TNFSF10 promoter, Fw-AAAGAAAATCCCTCCCCTCTT; Rv-CACTCACCTCAAGCCCATTT
SGK1 promoter, Fw- GGGAGGGAGAGGTCAGGAAT; Rv-TCGCTTGTTACCTCCTCACG

Animal studies and IHC

Animals were maintained and treated in accordance with Institutional Guidelines of Memorial Sloan Kettering Cancer Center (Protocol number 12-10-019). 5 x 10^6 cells in 1:1 PBS/Matrigel (Corning) were injected subcutaneously into six-week-old female athymic *Foxn1^{nu}* nude mice. When tumors reached a volume of ~150mm^3, mice were randomized, treated, and tumors were measured twice a week during a month. At least 10 tumors per group were used in all the studies. Treatments were as follows: BYL719 (25 mg × kg^{-1} in 0.5% carboxymethylcellulose (Sigma), daily p.o.); GSK2334470 (100 mg × kg^{-1} in 10% of 1:1 Kolliphor® EL/EtOH (Sigma), three times/week, i.p.); SGK1-inh (50 mg × kg^{-1} in 40% of 3:1 Glycofurol/Kolliphor® RH 40 mixture (Sigma) in 0.9% saline, daily p.o.). Tumors were harvested at the end of the experiment 3 hr after the last dosage, fixed in 4% formaldehyde in PBS, and paraffin-embedded. IHC was performed on a Ventana Discovery XT processor platform using standard protocols and the following antibodies from Cell Signaling Technology: pAKT(S473) (4060),1:70; pS6 (S240/4) (5364),1:500; pNDRG1 (T346) (5482), 1:200; p4EBP1 (T37/46) (2855), 1:300. Primary staining was followed by 60 minutes incubation with biotinylated goat anti-rabbit IgG (Vector labs) 1:200. Blocker D, Streptavidin- HRP and DAB detection kit (Ventana Medical Systems) were used according to the manufacturer instructions.

Docking and molecular dynamics simulations

The structure of SGK1 kinase is only available in its inactive form, with missing structural information such as the coordinates of the αC helix. We constructed the 3D structures of SGK1 kinase both in its active and inactive forms using comparative modeling methods based on homology. The templates used were the available crystal structure of SGK1 kinase in the inactive state (PDB: 2R5T) (Zhao et al., 2007), high-resolution crystal structure of the kinase domain of AKT (55% homology) in its active (PDB: 1O6K) (Yang et al., 2002a) and inactive (PDB: 1GZN) (Yang et al., 2002b) states. The program Modeller (version 9.12) (Sali and Blundell, 1993) was used for the generation of homology models. Several models were generated and the models with the best physicochemical properties were further refined using all atom molecular dynamics (MD) simulations.
The 3D structures of SGK1-inh and ATP were built using the Maestro module and minimized using the Macromodel module, employing the OPLS-2005 force field, in the program Schrodinger 9.0. The minimized SGK1 inhibitor and ATP were docked into the binding pockets of SGK1 kinase models with Glide (Friesner et al., 2004) using standard docking protocols (Kannan et al., 2015). Refinement of the docked models of SGK1-inhibitor and SGK1-ATP complexes were carried out using MD simulations under the Sander module of the program Amber14. The partial charges and force field parameters for SGK1 inhibitor and ATP were generated using the Antechamber module in Amber. All atom versions of the Amber 03 force field (ff03) (Duan et al., 2003) and the general Amber force field (GAFF) (Wang et al., 2004) were used for the protein and the inhibitors respectively. All the simulations were carried out at 300 K using standard protocols (Kannan et al., 2015). Three independent MD simulations (assigning different initial velocities) were carried out on each equilibrated SGK1-ATP and SGK1-inhibitor structure for 100 ns each, with conformations saved every 10 ps. Simulation trajectories were visualized using VMD (Humphrey et al., 1996) and figures were generated using Pymol.
The binding free energies (enthalpic components), energy decompositions (to identify "hot spot" residues) and

computational alanine scans (of the "hot spot" residues) were calculated using the MMPBSA (Molecular Mechanics Poisson–Boltzmann Surface Area) methodology (Kannan et al., 2015).

DNA methylation quantification

For DNA methylation analyses, bisulfite conversion of 500 ng of genomic DNA was performed using the EZ DNA Methylation Gold kit (Zymo Research, Orange, CA, USA) following the manufacturer's indications. For bisulfite sequencing, specific primers were designed to amplify the annotated promoter region using the MethylExpress program (Applied Biosystems) (Fw-AATTTTAGAATTTGGAAGAGGA and Rv-ACAACCTTAAATTAAACCCAAA), and a minimum of eight single clones was interrogated for each cell line. In order to quantify the absolute levels of DNA methylation on CpG sites in the proximity of the transcription start site of SGK1 we carried out pyrosequencing on bisulfite-treated DNA using specific primers designed with the PyroMark Assay Design Software (Qiagen, version 2.0.01.15) (Fw- GAGGGAGAGGTTAGGAATGT, Rv-CCCTCCCTTCRCTTATTACCTCCTCAC and Seq- TTTTGAAGTAATTTTTGAGAATATT). Pyrosequencing reactions and quantification of DNA methylation values were performed in a PyroMark Q96 System version 2.0.6 (Qiagen) including appropriate controls. As previously described, *SGK1* DNA methylation levels were categorized into three groups. DNA methylation values in the first group (<33%) were defined as low DNA methylation, and high DNA methylation was assigned to values on the two top groups (>33%).

FRET

For FRET experiments, HeLa cells were seeded in chambered coverglass and transfected with 0.5 μg of EGFP Donor plasmid, 0.5 μg of EYFP Acceptor plasmid, or both constructs. 16 hr post-transfection cells were imaged with a Leica TCS SP8 microscope using the established parameters for Donor (Ex: 458 nm laser at 15%; Em: 466-501 nm) and Acceptor (Ex: 528 nm laser at 3%; Em: 555-600 nm).
FRET efficiency was calculated using the following equation as described in (van Rheenen et al., 2004):

$$E_{\text{FRET}} = \frac{FRET - EGFP \times \beta - EYFP \times (\gamma - \alpha \times \beta)}{EYFP \times (1 - \beta \times \delta)}$$

Where FRET, EGFP and EYFP refers to the FRET, Donor and Acceptor channels respectively. The corrections factors were α=0.01; β=0.37; γ=0.31; δ=0.02, where α corrects for acceptor cross-excitation crosstalk (α=Donor/Acceptor), β corrects for donor crosstalk (β=FRET/Donor), γ corrects for acceptor cross-excitation (γ=FRET/Acceptor), and δ corrects for FRET crosstalk (δ=Donor/FRET). Mock-transfected cells were used to calculate the background threshold level (background intensity mean + 4 Standard deviation).

TMA and patients

Formalin-fixed paraffin-embedded (FFPE) tissue blocks from primary invasive breast carcinomas were used to construct the TMA reported in this study. A certified pathologist (E.B.) microscopically examined hematoxylin and eosin-stained sections of all the tumors and selected representative areas, excluding foci of ductal carcinoma in situ and tumor necrosis. All carcinomas were represented in the TMAs in triplicate 0.6-mm cores. An Automatic Tissue MicroArrayer (ATA-27, Beecher Instruments Inc) was used to construct TMAs from a total of 273 breast invasive carcinomas. This comprised clinically and pathologically confirmed triple-negative breast cancer patients (138), ER/PR receptor-positive breast cancer patients (68), and HER2-positive cancer patients (67). Tumor were considered ER/PR receptor-positive if >10% of neoplastic cells showed nuclear positivity. Cases with HER-2 staining intensity of 3+ were considered positive, whereas those with 2+ staining intensity of HER2 were further evaluated by ERBB2 FISH using the PathVision HER2 probe Kit (Abbott Laboratories), and scored as positive if the HER2/Cep17 ratio was 2.2 or greater. 5-μm thick TMA sections were stained for pNDRG1 (T346) following the protocol described above. Based on the observed staining across the different samples, cases were scored as High expression when pNDRG1 staining intensity of 2+ was found >20% of the neoplastic cells. Intermediate staining represented tumors that had 1+ staining intensity in >10% of the neoplastic cells.
For the study of patients treated with the PI3Kα inhibitor BYL719, pre-treatment biopsy FFPE blocks from patients enrolled in the clinical trial NCT01870505 conducted at MSKCC were used for IHC as described above. For the selection of the patients, *PIK3CA* and other tumor genomic drivers were analyzed using MSK-IMPACT (Cheng et al., 2015). Only patients that did not exhibit toxicity during the trial, harbored hot-spot mutations in *PIK3CA*, and

did not harbor mutations in *PTEN* or *KRAS* (known to cause resistance to PI3Kα inhibitors) were selected for the biomarker study. *SGK1* mRNA levels were determined using next-generation sequencing (NGS) and the expression results were presented as raw Reads Per Kilobase of transcript per Million mapped reads (RPKM). Mean and standard deviation (SD) was calculated across all the samples with available NGS data and overexpression of *SGK1* was called for the samples with mRNA levels greater than mean+1SD. For 6 samples, RNA quality and quantity was not optimal for NGS. In these cases, *SGK1* mRNA levels were determined using RT-qPCR as described above. RNA from low expressing (T47D) and high expressing (HCC1954) cell lines were used as positive control and absolute mRNA levels were quantified. Primers used for the detection of *SGK1* were:
Fw: GACAGGACTGTGGACTGGTG; Rv: TTTCAGCTGTGTTTCGGCTA
The MSKCC Institutional Review Board approved the study and informed consent was obtained from all subjects.

Supplemental References

Bhinder, B., and Djaballah, H. (2012). A simple method for analyzing actives in random RNAi screens: introducing the "H Score" for hit nomination & gene prioritization. Comb Chem High Throughput Screen *15*, 686-704.

Cheng, D. T., Mitchell, T. N., Zehir, A., Shah, R. H., Benayed, R., Syed, A., Chandramohan, R., Liu, Z. Y., Won, H. H., Scott, S. N.*, et al.* (2015). Memorial Sloan Kettering-Integrated Mutation Profiling of Actionable Cancer Targets (MSK-IMPACT): A Hybridization Capture-Based Next-Generation Sequencing Clinical Assay for Solid Tumor Molecular Oncology. J Mol Diagn *17*, 251-264.

Duan, Y., Wu, C., Chowdhury, S., Lee, M. C., Xiong, G., Zhang, W., Yang, R., Cieplak, P., Luo, R., Lee, T.*, et al.* (2003). A point-charge force field for molecular mechanics simulations of proteins based on condensed-phase quantum mechanical calculations. J Comput Chem *24*, 1999-2012.

Fellmann, C., Hoffmann, T., Sridhar, V., Hopfgartner, B., Muhar, M., Roth, M., Lai, D.Y., Barbosa, I.A.M., Kwon, J.S., Guan, Y., Sinha, N., Zuber, J. (2013). An Optimized microRNA Backbone for Effective Single-Copy RNAi. Cell Reports *5 (6)*,1704–1713.

Friesner, R. A., Banks, J. L., Murphy, R. B., Halgren, T. A., Klicic, J. J., Mainz, D. T., Repasky, M. P., Knoll, E. H., Shelley, M., Perry, J. K.*, et al.* (2004). Glide: a new approach for rapid, accurate docking and scoring. 1. Method and assessment of docking accuracy. J Med Chem *47*, 1739-1749.
Garcia-Martinez, J. M., and Alessi, D. R. (2008). mTOR complex 2 (mTORC2) controls hydrophobic motif phosphorylation and activation of serum- and glucocorticoid-induced protein kinase 1 (SGK1). Biochem J *416*, 375-385.

Humphrey, W., Dalke, A., and Schulten, K. (1996). VMD: visual molecular dynamics. J Mol Graph *14*, 33-38, 27-38.

Kannan, S., Poulsen, A., Yang, H. Y., Ho, M., Ang, S. H., Eldwin, T. S., Jeyaraj, D. A., Chennamaneni, L. R., Liu, B., Hill, J.*, et al.* (2015). Probing the binding mechanism of Mnk inhibitors by docking and molecular dynamics simulations. Biochemistry *54*, 32-46.

Sali, A., and Blundell, T. L. (1993). Comparative protein modelling by satisfaction of spatial restraints. J Mol Biol *234*, 779-815.

Shevchenko, A., Tomas, H., Havlis, J., Olsen, J. V., and Mann, M. (2006). In-gel digestion for mass spectrometric characterization of proteins and proteomes. Nat Protoc *1*, 2856-2860.

Toska, E., Campbell, H. A., Shandilya, J., Goodfellow, S. J., Shore, P., Medler, K. F., and Roberts, S. G. (2012). Repression of transcription by WT1-BASP1 requires the myristoylation of BASP1 and the PIP2-dependent recruitment of histone deacetylase. Cell Rep *2*, 462-469.

van Rheenen, J., Langeslag, M., and Jalink, K. (2004). Correcting confocal acquisition to optimize imaging of fluorescence resonance energy transfer by sensitized emission. Biophys J *86*, 2517-2529.

Wang, J., Wolf, R. M., Caldwell, J. W., Kollman, P. A., and Case, D. A. (2004). Development and testing of a general amber force field. J Comput Chem *25*, 1157-1174.

Yang, J., Cron, P., Good, V. M., Thompson, V., Hemmings, B. A., and Barford, D. (2002a). Crystal structure of an activated Akt/protein kinase B ternary complex with GSK3-peptide and AMP-PNP. Nat Struct Biol *9*, 940-944.

Yang, J., Cron, P., Thompson, V., Good, V. M., Hess, D., Hemmings, B. A., and Barford, D. (2002b). Molecular mechanism for the regulation of protein kinase B/Akt by hydrophobic motif phosphorylation. Mol Cell *9*, 1227-1240.

Zhao, B., Lehr, R., Smallwood, A. M., Ho, T. F., Maley, K., Randall, T., Head, M. S., Koretke, K. K., and Schnackenberg, C. G. (2007). Crystal structure of the kinase domain of serum and glucocorticoid-regulated kinase 1 in complex with AMP PNP. Protein Sci *16*, 2761-2769.
